# An easy direct arylation of 5-pyrazolones

**DOI:** 10.3762/bjoc.9.240

**Published:** 2013-10-08

**Authors:** Hao Gong, Yiwen Yang, Zechao Wang, Chunxiang Kuang

**Affiliations:** 1 Department of Chemistry, Tongji University, Siping Road 1239, Shanghai 200092, China; 2 College of Biological, Chemical Sciences and Engineering, Jiaxing University, Jiaxing 314001,China; 3 Key Laboratory of Yangtze River Water Environment, Ministry of Education, Shanghai 200092, China

**Keywords:** arylation, aryl halide, C–H bond activation, Pd(OAc)_2_, pyrazolone

## Abstract

A mild, efficient and catalytic ligand-free method for the direct arylation of 5-pyrazolones by Pd-catalyzed C–H bond activation is reported. The process smoothly proceeds and yields are moderate to excellent.

## Introduction

5-Pyrazolones are attracting considerable research interest because of their unique chemical properties and their structures that facilitate their application as biological and pharmaceutical intermediates and products [1–3]. Over the years, many of the biological activities of pyrazolones such as their antipyretic, analgesic [4–5], anti-inflammatory [6–7], antitumor [8–9], antiviral, antibacterial [10], and herbicidal [11] properties have been discovered and investigated. Pyrazolones are also potent inhibitors of telomerase, cyclooxygenase isoenzymes, platelet tromboxane synthesis, and prostanoid synthesis in humans [12–13]. Recently, pharmacologists have developed a novel class-II c-met inhibitor, whose structural unit is a pyrazolone ring [14]. The great medicinal significance and broad applications of pyrazolones prompted us to synthesize a new series of heterocyclic compounds containing the pyrazolone moiety.

The reaction of pyrazolones with arylboronic acids is an attractive approach for the synthesis of arylpyrazolone [15–16]. However, it often needs pre-formation of halo-pyrazolones. Transition metal-catalyzed direct arylation of (hetero)arenes has emerged over the past few years as a rapidly growing field of syntheses [17–26]. The direct arylation of pyrazolones by using aryl halides offers a cleaner and more efficient method of meeting such goals and rare examples of such transformations have been described [15].

**Scheme 1:**
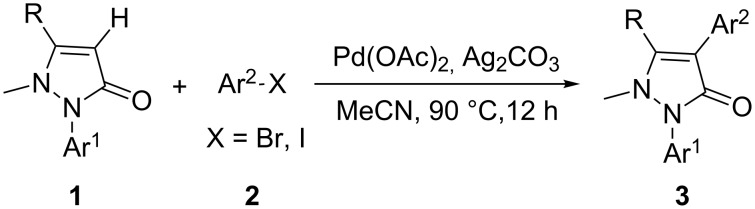
Direct arylation of 5-pyrazolones.

In this paper, we report a convenient and catalytic ligand-free synthesis of a series of 4-aryl-5-pyrazolones **3** from 5-pyrazolones **1** and aryl halides **2** (Scheme 1). The direct arylation of 5-pyrazolones by Pd-catalyzed C–H bond activation was utilized.

## Results and Discussion

We commenced this study by performing the direct arylation of phenazone (**1a**) in the presence of 2 equiv of iodobenzene (**2a**), 10 mol % of Pd(OAc)_2_ as a catalyst in acetonitrile in a sealed tube. The results are shown in Table 1. Gratifyingly, a 45% yield of the desired product **3a** was achieved after stirring for 12 h at 90 °C. Encouraged by this preliminary result, we continued to optimize reaction conditions to further improve the chemical yield.

**Table 1:** Optimization of the synthesis of **3a**^a^.

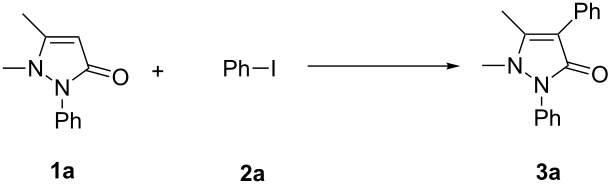					

entry	additive (2 equiv)	catalyst (0.1 equiv)	solvent	*T* (°C)	yield of **3a**^b^

1	none	Pd(OAc)_2_	CH_3_CN	90	45
2	K_2_CO_3_	Pd(OAc)_2_	CH_3_CN	90	43
3	Cs_2_CO_3_	Pd(OAc)_2_	CH_3_CN	90	35
4	Na_2_CO_3_	Pd(OAc)_2_	CH_3_CN	90	27
5	DBU	Pd(OAc)_2_	CH_3_CN	90	0
6	K_3_PO_4_	Pd(OAc)_2_	CH_3_CN	90	49
7	Ph_3_P (0.25 equiv)	Pd(OAc)_2_	CH_3_CN	90	42
8	none	Pd(OAc)_2_ (0.05 equiv)	CH_3_CN	90	40
9	none	Pd(OAc)_2_ (0.02 equiv)	CH_3_CN	90	32
10	none	Pd(OAc)_2_	THF	90	traces
11	none	Pd(OAc)_2_	DCE	90	31
12	none	Pd(OAc)_2_	dioxane	90	0
13	none	Pd(OAc)_2_	benzene	90	22
14	none	Pd(OAc)_2_	CH_3_CN	25	0
15	none	Pd(OAc)_2_	CH_3_CN	60	31
16	none	Pd(OAc)_2_	CH_3_CN	120	35
17	O_2_(1atm)	Pd(OAc)_2_	CH_3_CN	90	55
18	K_2_S_2_O_8_	Pd(OAc)_2_	CH_3_CN	90	5
19	benzoquinone	Pd(OAc)_2_	CH_3_CN	90	0
20	Cu(OAc)_2_	Pd(OAc)_2_	CH_3_CN	90	25
**21**	**Ag_2_CO_3_**	**Pd(OAc)_2_**	**CH_3_CN**	**90**	**80**
22	none	FeCl_3_ (0.3 equiv)	CH_3_CN	90	0
23	none	Cu(OAc)_2_ (0.2 equiv)	CH_3_CN	90	0
24	none	none	CH_3_CN	90	0

^a^Reaction conditions: 1.0 equiv of **1a** and 2.0 equiv of **2a** were stirred for 12 h. ^b^Isolated yield.

When **1a** reacted with **2a** in the presence of K_2_CO_3_ as a base in acetonitrile (90 °C, 12 h), the desired product **3a** was generated in 43% yield (Table 1, entry 2). Changing K_2_CO_3_ to Cs_2_CO_3_, Na_2_CO_3_ and DBU (1,8-diazabicyclo(5.4.0)undec-7-ene), decreased the yield to 35%, 27% and 0%, respectively (Table 1, entry 3–5). Changing K_2_CO_3_ to K_3_PO_4_, the yield was increased to 49% (Table 1, entry 6). When Ph_3_P as a catalytic ligand was added to the reaction, the yield decreased to 42% (Table 1, entry 7). Reducing the dosage of Pd(OAc)_2_ to 0.05 equiv and 0.02 equiv, respectively, decreased the yield to 40% and 32% (Table 1, entries 8–9). Several solvents were examined under the conditions of entry 1. When the solvent was changed to THF, DCE, dioxane, and benzene, the yields decreased to trace, 31%, 0% and 22%, respectively (Table 1, entries 10–13). Other reaction parameters such as temperature and oxidants were also screened. When the reaction temperatures were 25 °C, 60 °C, and 120 °C, the yields decreased to 0%, 31% and 35%, respectively (Table 1, entries 14–16). When the reaction was under oxygen (1 atm) in a sealed tube and oxygen was used as an oxidant, product **3a** was obtained in 55% yield (Table 1, entry 17). Changing the oxidant to K_2_S_2_O_8_, benzoquinone and Cu(OAc)_2_ decreased the yield to 5%, 0% and 25%, respectively (Table 1, entries 18–20). When Ag_2_CO_3_ was added to the reaction, the yield increased to 80% (Table 1, entry 21). Different catalysts were also examined. When Cu(OAc)_2_ or FeCl_3_ was used as a catalyst, or no catalyst was used in the reaction, product **3a** was not obtained (Table 1, entries 22–24). Ultimately, the optimal reaction conditions were determined to be 0.1 equiv Pd(OAc)_2_ catalyst, 2.0 equiv Ag_2_CO_3_, acetonitrile, 90 °C, air atmosphere, 1:2 molar ratio of **1a** to **2a**, and 12 h reaction time.

Under the optimized conditions (Table 1, entry 10), the scope of aryl halides was examined and the results are summarized in Table 2. The reactions of aryl halides **2** with phenyl moieties carrying either an electron-donating group such as methyl (**2d** and **2i**), ethyloxy (**2e**) or an electron-withdrawing substituent such as methoxycarbonyl (**2c** and **2g**), trifluoromethyl (**2f**) or formyl (**2h**) proceeded smoothly with moderate to good yields (Table 2, entries 3–10). When the phenyl moiety of the aryl halides **2** carried an electron-donating group, higher yields were obtained (Table 2, entries 4, 5, 9). On the other hand, an electron-withdrawing group on the phenyl moiety of the aryl halides (**2c**, **2f**, **2g** and **2h**) provided 4-aryl-5-pyrazolones **3** in relatively low yields (Table 2, entries 3, 6–8). Entries 1 and 2 show that the yield of products was lower when using aryl bromide than when using aryl iodide, and 2-bromopyridine also provided **3i** in moderate yield (Table 2, entry 10).

**Table 2:** Synthesis of 4-aryl- 5-pyrazolones **3**.

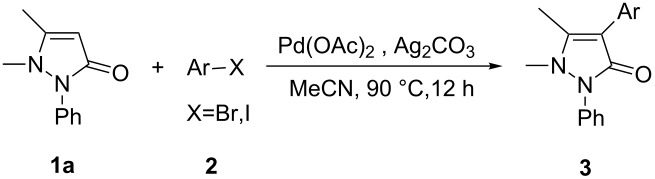			

entry	Ar–X	product	yield of **3** (%)^a^

1	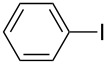 **2a**	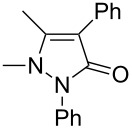 **3a**	80
2	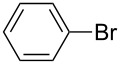 **2b**	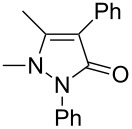 **3a**	67
3	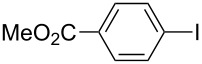 **2c**	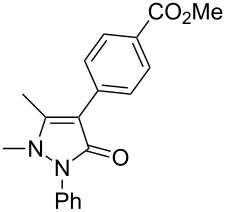 **3b**	71
4	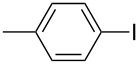 **2d**	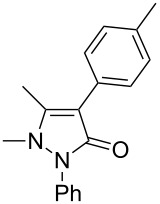 **3c**	81
5	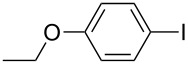 **2e**	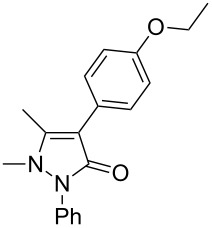 **3d**	84
6	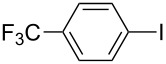 **2f**	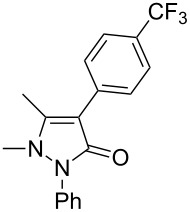 **3e**	71
7	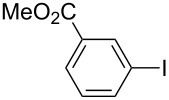 **2g**	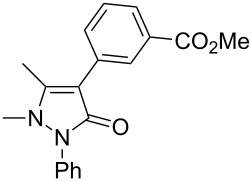 **3f**	78
8	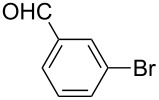 **2h**	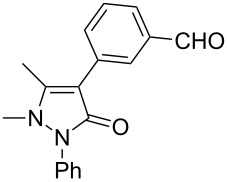 **3g**	70
9	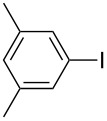 **2i**	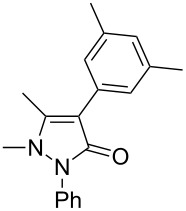 **3h**	82
10	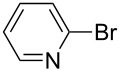 **2j**	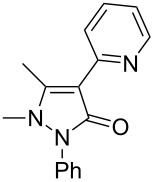 **3i**	64

^a^Isolated yield.

Next, we investigated the scope of 5-pyrazolone **1** substrates. Table 3 shows that in most cases, the desired pyrazolones **3** were generated smoothly in moderate to good yields. When the phenyl moiety of pyrazolones **1** carried an electron-donating substituent such as methoxy (**1b**) and methyl (**1c**), the reactions provided pyrazolones **3** in high yields (Table 3, entries 1, 2). On the other hand, when pyrazolones **1** carried an electron-withdrawing substituent such as nitro (**1f**) and halogens (**1g**, **1i** and **1k**) in the aromatic portion, relatively low yields were obtained (Table 3, entries 5, 6, 8, 10). Compared with 5-pyrazolones containing a butyl or a phenyl substituent on the 3-position of the heterocycle (**1d** and **1e)**, the methyl (**1a**) on the same position resulted in a higher yield (Table 3, entries 3 and 4). The cause might be the steric hindrance of phenyl or butyl. The same trend could be seen from **1g** to **1l** (cf. **3o**, **3q** and **3s** with **3p**, **3r** and **3t**) (Table 3, entries 6–11).

**Table 3:** Synthesis of 4-phenyl-5-pyrazolones **3**.

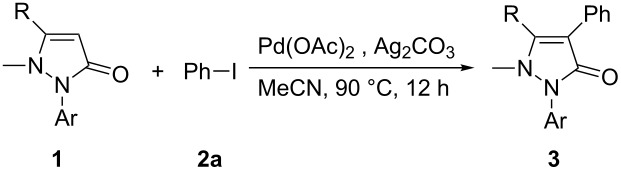			

entry	5-pyrazolone	product	yield of **3** (%)^a^

1	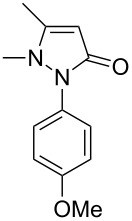 **1b**	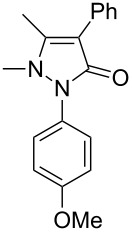 **3j**	87
2	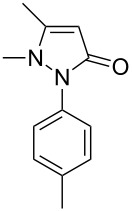 **1c**	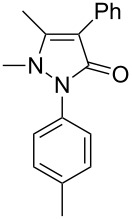 **3k**	83
3	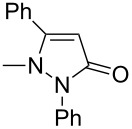 **1d**	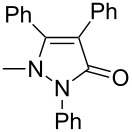 **3l**	53
4	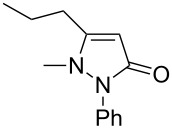 **1e**	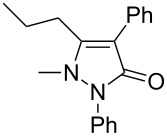 **3m**	66
5	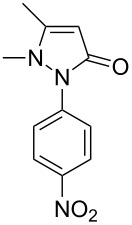 **1f**	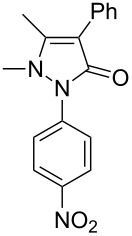 **3n**	51
6	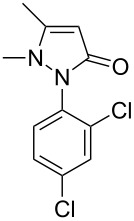 **1g**	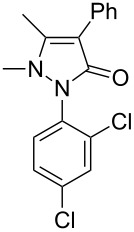 **3o**	69
7	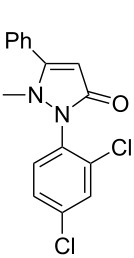 **1h**	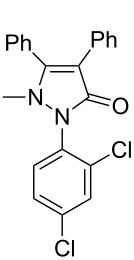 **3p**	41
8	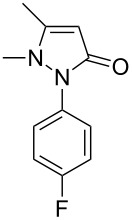 **1i**	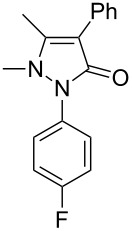 **3q**	62
9	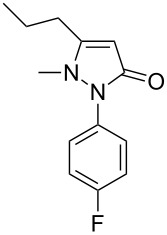 **1j**	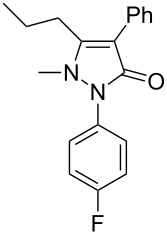 **3r**	47
10	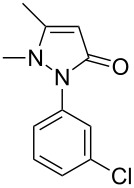 **1k**	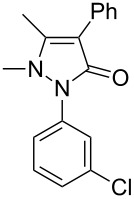 **3s**	71
11	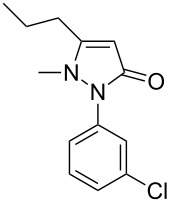 **1l**	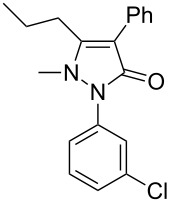 **3t**	59

^a^Isolated yield.

## Conclusion

In summary, we developed a mild, simple and efficient method for the direct arylation of 5-pyrazolones by Pd-catalyzed C–H bond activation. This approach resulted in the construction of 4-aryl-5-pyrazolones, which are important heterocyclic compounds used in medicinal and biological research. The investigations on the reaction mechanism are still in progress.

## Supporting Information

File 1 Experimental details and characterization data for all compounds.
