# Supplementary material for: An easy direct arylation of 5-pyrazolones
Source: Beilstein J Org Chem. 2013 Oct 8;9:2033–9. doi: 10.3762/bjoc.9.240 (PMC3817516; doi:10.3762/bjoc.9.240)
Supplement: File 1 — Experimental details and characterization data for all compounds. [file Beilstein_J_Org_Chem-09-2033-s001.pdf]

Supporting Information

for

An easy direct arylation of 5-pyrazolones

Hao Gong[1], Yiwen Yang[1,2], Zechao Wang[1] and Chunxiang Kuang*[1,3]

Address: [1]Department of Chemistry, Tongji University, Siping Road 1239, Shanghai 200092, China, [2]College of Biological, Chemical Sciences and Engineering, Jiaxing University, Jiaxing 314001,China and [3]Key Laboratory of Yangtze River Water Environment, Ministry of Education, Shanghai 200092, China

Email: Chunxiang Kuang* - kuangcx@tongji.edu.cn.

* Corresponding author

Experimental details and characterization data for all compounds

Contents

1) Experimental procedures……………………………………………2

2) Experimental details and characterization data for 5-pyrazolones **1**.….2

3) Experimental details and characterization data for products **3**……..…5

4) ^{1}H NMR and ^{13}C NMR spectra for all compounds …………………13

1. Experimental procedures

All commercially available reagents and solvents were obtained from the commercial providers and used without further purification. Melting points were recorded using a WRS-2A melting point apparatus and were uncorrected. IR spectra were obtained on a Nexus FT-IR spectrophotometer. ^{1}H NMR and ^{13}C NMR spectra were recorded using a Bruker Avance 400 MHz spectrometer. Chemical shifts were reported relative to tetramethylsilane (0.00 ppm) for ^{1}H and $CDCl_3$ (77.0 ppm) for ^{13}C. High resolution mass spectra were determined using a Finnigan-NAT GC/MS/DS 8430 spectrometer. Flash column chromatography was performed on 300–400 mesh silica gel. 5-Pyrazolones **1** (except **1a**) were prepared according to literature procedures [1]. 5-Pyrazolone **1a** is a commercially available reagent.

2. Experimental details and characterization data for 5-pyrazolones 1

1) Experimental details and characterization data for 1b–l

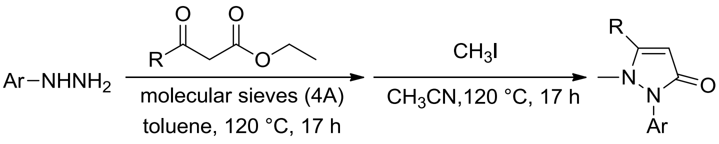

A reaction mixture containing aryl hydrazine (2 mmol), ethyl acetoacetate (2 mmol), molecular sieves (4 Å) and toluene (4 mL) was stirred in the dark in a sealed tube maintained at 120 °C in an oil bath. After 17 h, the reaction mixture was cooled to room temperature. To this mixture, acetonitrile (2 mL) and iodomethane (6 mmol) were added successively and stirred in the dark in the sealed tube maintained at 120 °C for additional 17 h, After the reaction was completed, the mixture was cooled to room temperature. The solvent was then evaporated in vacuo. The resulting residue was purified by flash column chromatography (ethyl acetate) to yield **1b–l**.

Spectral data of 1b–l

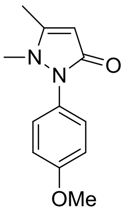

1b

2-(4-Methoxyphenyl)-1,5-dimethyl-1*H*-pyrazol-3(2*H*)-one(1b) [1]**:**

yellow solid, mp:124.7-125.8°C. ^{1}H NMR (400 MHz, $CDCl_3$): δ = 7.29 (d, J = 8.8 Hz, 2H), 6.99 (d, J = 8.8 Hz, 2H), 5.40 (s, 1H), 3.85 (s, 3H), 3.07 (s, 3H), 2.24 (s, 3H); ^{13}C NMR (100 MHz, $CDCl_3$): δ = 166.5, 158.8, 154.9, 127.9, 127.0 (2C), 114.6 (2C), 97.9, 55.6, 34.8, 12.9; IR (KBr): 3002, 2823, 1629, 1565, 1552, 1530, 1483, 1452, 1384, 1275 cm^{-1}; HR-MS (ESI): m/z calcd for $C_{12}H_{14}N_2O_2$: 218.1126 $[M]^+$; found: 218.1135.

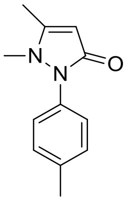

1c

1,2-Dihydro-2,3-dimethyl-1-*p*-tolylpyrazol-5-one (1c):
yellow solid, mp: 147.8-148.6 °C. ^{1}H NMR (400 MHz, $CDCl_3$): δ = 7.25(s, 4H), 5.38 (s, 1H), 3.06 (s, 3H), 2.38 (s, 3H), 2.23 (s, 3H); ^{13}C NMR (100 MHz, $CDCl_3$): δ = 166.4, 155.7, 136.9, 132.6, 129.8 (2C) , 124.7 (2C) , 98.4, 35.1, 21.1, 12.9; IR (KBr): 3060, 2911, 1630, 1565, 1547, 1513, 1483, 1384 cm^{-1}; HR-MS (ESI): *m/z* calcd for $C_{12}H_{14}N_2O$: 202.1132 $[M]^+$; found: 202.1128.

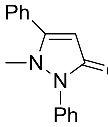

1d

1,2-Dihydro-2-methyl-1,3-diphenylpyrazol-5-one (1d):
yellow solid, mp: 157.2-158.5°C. ^{1}H NMR (400 MHz, $CDCl_3$): δ = 7.60-7.45 (m, 9H), 7.29 (t, *J* = 7.2 Hz, 1H), 5.82 (s, 1H), 3.02 (s, 3H); ^{13}C NMR (100 MHz, $CDCl_3$): δ = 165.6, 161.6, 135.2, 130.6, 129.3, 129.2 (2C), 129.1 (2C), 128.4 (2C), 123.7 (2C), 126.5, 100.2, 39.1; IR (KBr): 3087, 2943, 2878, 1630, 1565, 1552, 1513, 1484 cm^{-1}; HR-MS (ESI): *m/z* calcd for $C_{16}H_{14}N_2O$: 250.1107 $[M]^+$; found: 250.1103.

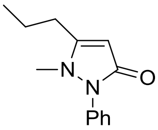

1e

1-Methyl-2-phenyl-5-propyl-1H-pyrazol-3(2H)-one (1e):
yellow solid, mp: 62.9-63.7 °C. ^{1}H NMR (400 MHz, $CDCl_3$): δ = 7.43 (t, *J* = 7.8 Hz, 2H), 7.35 (d, *J* = 7.5 Hz, 2H), 7.26 (t, *J* = 7.3 Hz, 1H), 5.39 (s, 1H), 3.03 (s, 3H), 2.47 (t, *J* = 7.6 Hz, 2H), 1.76 -1.64 (m, 2H), 1.04 (t, *J* = 7.4 Hz, 3H); ^{13}C NMR (100 MHz, $CDCl_3$): δ = 166.5, 160.9, 135.2 , 129.2 (2C), 126.61, 124.3 (2C), 97.6, 35.6, 28.9, 20.8, 13.8. IR (KBr): 3085, 2958, 2931, 1658, 1594, 1569, 1454, 1396, 1312, 906, 833 cm^{-1}; HR-MS (ESI): *m/z* calcd for $C_{13}H_{16}N_2O$: 216.1261$[M]^+$; found: 216.1275.

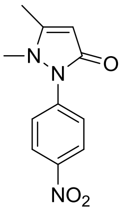

1f

1,2-Dihydro-2,3-dimethyl-1-(4-nitrophenyl)pyrazol-5-one (1f) [2]**:**
tan solid, mp:164.8-165.6 °C.^{1}H NMR (400 MHz, $CDCl_3$): δ = 8.30 (d, *J* = 9.2 Hz, 2H), 7.59 (d, *J* = 8.8 Hz, 2H), 5.45 (s, 1H), 3.09 (s, 3H), 2.28 (s, 3H); ^{13}C NMR (100 MHz, $CDCl_3$): δ = 166.4, 160.7, 144.7, 141.1, 124.7 (2C) , 122.2 (2C) , 100.0, 36.7,13.3; IR (KBr): 3010, 2944, 2893, 1630, 1563, 1530, 1513, 1481, 1352, 1324 cm^{-1}; HR-MS (ESI): *m/z* calcd for $C_{11}H_{11}N_3O_3$: 233.0816 $[M]^+$; found: 233.0822.

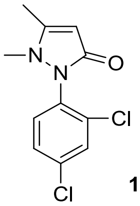

1g

1-(2,4-Dichlorophenyl)-1,2-dihydro-2,3-dimethylpyrazol-5-one (1g):
yellow solid, mp:139.2-140.1°C. ^{1}H NMR (400 MHz, $CDCl_3$): δ = 7.50 (s, 1H), 7.33 (d, *J* = 8.0Hz, 1H), 7.26 (d, *J* = 8.4 Hz, 1H), 5.28 (s, 1H), 3.02 (s, 3H), 2.20 (s, 3H); ^{13}C NMR (100 MHz, $CDCl_3$): δ = 166.5, 155.1, 135.8, 134.9, 131.6, 131.5, 130.7, 128.1, 96.8, 34.2, 12.9; IR (KBr): 3042, 2930, 2839, 1672, 1599, 1552, 1530, 1481, 1468, 1332, 621 cm^{-1}; HR-MS (ESI): *m*/*z* calcd for $C_{11}H_{10}{}^{35}Cl_2N_2O$: 256.0224 $[M]^+$; found: 256.0215.

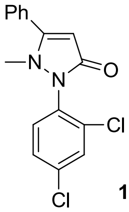

1h

1-(2,4-Dichlorophenyl)-1,2-dihydro-2-methyl-3-phenylpyrazol-5-one (1h):
brown solid, mp: 119.6- 120.5°C. ^{1}H NMR (400 MHz, $CDCl_3$): δ =7.55 (s, 1H), 7.54-7.46 (m, 5H), 7.41 (d, *J* = 8.4 Hz, 1H), 7.37 (d, *J* = 8.4 Hz, 1H), 5.73 (s, 1H), 2.98 (s, 3H); ^{13}C NMR (100 MHz, $CDCl_3$): δ = 165.8, 160.8, 135.7, 134.4, 131.5, 131.2, 130.7, 129.1 (2C), 130.6, 129.0, 128.4 (2C) , 128.1, 98.5, 37.6; IR (KBr): 3081, 2969, 1666, 1582, 1550, 1530, 1469, 1384, 841,735 cm^{-1}; HR-MS (ESI): *m*/*z* calcd for $C_{16}H_{12}{}^{35}Cl_2N_2O$: 318.0321 $[M]^+$; found: 318.0316.

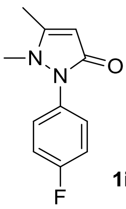

1i

2-(4-Fluorophenyl)-1,5-dimethyl-1*H*-pyrazol-3(2*H*)-one (1i) [1]**:**
brown solid, mp: 94.3-95.1°C. ^{1}H NMR (400 MHz, $CDCl_3$): δ = 7.31-7.27 (m, 2H), 7.11 (t, *J* = 8.6 Hz, 2H), 5.29 (s, 1H), 3.08 (s, 3H), 2.25 (s, 3H); ^{13}C NMR (100 MHz, $CDCl_3$) δ = 166.2, 161.4 (d, *J* = 245.7 Hz, 1C), 156.1, 130.8 (d, *J* = 2.8 Hz, 1C), 126.8 (d, *J* = 8.4 Hz, 2C), 116.2 (d, *J* = 22.8 Hz, 2C), 97.7, 35.2, 13.1; IR (KBr): 3111, 3047, 2999, 1651, 1506, 1424, 1348, 836, 821, 770 cm^{-1}; HR-MS (ESI): *m*/*z* calcd for $C_{11}H_{11}{}^{19}FN_2O$: 206.0864 $[M]^+$; found: 206.0854

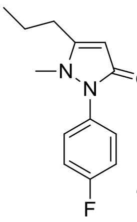

1j

2-(4-Fluorophenyl)-1-methyl-5-propyl-1*H*-pyrazol-3(2*H*)-one (1j) :

tan solid, mp: 95.8-96.7°C. ^{1}H NMR (400 MHz, $CDCl_3$): δ = 7.35-7.30 (m, 2H), 7.12 (t, *J* = 8.6 Hz, 2H), 5.37 (s, 1H), 3.02 (s, 3H), 2.46 (t, *J* = 7.6 Hz, 2H), 1.76 – 1.64 (m, 2H), 1.03 (t, *J* = 7.4 Hz, 3H); ^{13}C NMR (100 MHz, $CDCl_3$): δ = 166.6, 161.13, 161.12 (d, *J* = 245.1 Hz, 1C), 131.2 (d, *J* = 2.8 Hz, 1C), 126.3 (d, *J* = 8.5 Hz, 2C), 116.1 (d, *J* = 22.7 Hz, 2C), 97.5, 35.5, 28.8, 20.8, 13.7; IR (KBr): 3050, 2962, 1661, 1602, 1505, 1422, 1333, 962, 836, 811, 780 cm^{-1}; HR-MS (ESI): *m*/*z* calcd for $C_{13}H_{15}{}^{19}FN_2O$: 234.1157[M]$^+$; found: 234.1165.

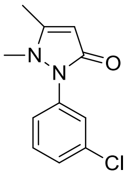

1k

1-(3-Chlorophenyl)-1,2-dihydro-2,3-dimethylpyrazol-5-one (1k) [1]:

gray solid, mp:104.3-105.9°C. ^{1}H NMR (400 MHz, $CDCl_3$): δ = 7.40 (s, 1H), 7.39 (t, *J* = 7.8 Hz, 1H), 7.33 (d, *J* = 8.0 Hz, 1H), 7.26 (d, *J* = 7.6 Hz, 1H), 5.42 (s, 1H), 3.07 (s, 3H), 2.26 (s, 3H); ^{13}C NMR (100 MHz, $CDCl_3$): δ = 166.5, 158.1, 136.6, 134.8, 130.1, 126.4, 123.6, 121.9, 99.2, 35.9, 13.1; IR (KBr): 3025, 2971, 1630, 1582, 1548, 1513, 1483, 1451, 1384, 769 cm^{-1}; HR-MS (ESI): *m*/*z* calcd for $C_{11}H_{11}{}^{35}ClN_2O$: 222.0612 [M]$^+$; found: 222.0608.

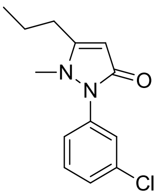

1l

2-(3-Chlorophenyl)-1-methyl-5-propyl-1*H*-pyrazol-3(2*H*)-one (1l):

yellow solid, mp: 63.4-64.1°C. ^{1}H NMR (400 MHz, $CDCl_3$): δ= 7.37 (t, *J* = 7.8 Hz, 2H), 7.29 (d, *J* = 8.4 Hz, 1H), 7.23 (d, *J* = 7.5 Hz, 1H), 5.39 (s, 1H), 3.04 (s, 3H), 2.47 (t, *J* = 7.6 Hz, 2H), 1.76-1.65 (m, 2H), 1.04 (t, *J* = 7.3 Hz, 3H); ^{13}C NMR (100 MHz, $CDCl_3$): δ = 166.6, 162.5, 136.5, 134.7, 130.1, 126.4, 123.7, 121.9, 98.1, 36.0, 28.9, 20.8, 13.8; IR (KBr): 3072, 2987, 1655, 1573, 1531, 1465, 1432, 1314, 972, 894, 778 cm^{-1}; HR-MS (ESI): *m*/*z* calcd for $C_{13}H_{15}{}^{35}ClN_2O$: 250.0865 [M]$^+$; found: 250.0857.

3. Experimental details and characterization data for products 3

A mixture of 5-pyrazolones **1** (0.3 mmol), aryl halids (0.6 mmol), $Pd(OAc)_2$ (0.03 mmol) and Ag_2CO_3 (0.6 mmol) in 2 mL MeCN was placed in a sealed tube. The tube was heated at 90 °C for 12 h using an oil bath. After the reaction was completed (as monitored by TLC), the mixture was cooled to room temperature, and then concentrated under reduced pressure to afford a crude product.

Purification by column chromatography on silica gel (EtOAc) to yield 4-aryl-5-pyrazolones **3**.

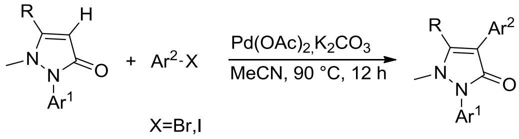

Spectral data of 3

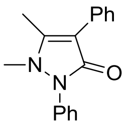

3a

1,2-Dihydro-2,3-dimethyl-1,4-diphenylpyrazol-5-one (3a)**:**

light yellow solid. mp:160.0-160.8 °C. ^{1}H NMR (400MHz, $CDCl_3$): δ = 7.57 (d, *J* = 7.6 Hz, 2H), 7.49 (d, *J* = 4.4 Hz, 4H), 7.42 (t, *J* = 7.8 Hz, 2H), 7.34-7.29 (m, 2H), 3.17 (s, 3H), 2.40 (s, 3H); ^{13}C NMR (100MHz, $CDCl_3$): δ = 164.6, 152.2, 135.3, 131.3, 129.1(2C) , 128.9 (2C) , 128.3 (2C) , 126.8, 126.5, 124.1(2C), 111.2, 36.0, 12.1; IR (KBr): 3158, 3086, 3050, 1598, 1527, 1514, 1501, 1465, 1457, 1384, 1338, 1064, 994, 957, 855, 791, 757, 703, 685 cm^{-1}; HR-MS: m/z calcd for $C_{17}H_{16}N_2O$: 264.1284 $[M]^+$; found: 264.1276.

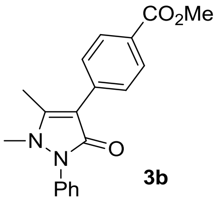

3b

Methyl 4-(1,5-dimethyl-3-oxo-2-phenyl-2,3-dihydro-1*H*-pyrazol-4-yl)benzoate (3b) :

yellow solid. mp 148.2-149.3 °C. ^{1}H NMR (400 MHz, $CDCl_3$): δ = 8.90 (d, *J* = 8.4 Hz, 2H), 7.68 (d, *J* = 8.4 Hz, 2H), 7.52-7.45 (m, 4H), 7.34(t, *J* = 7.2 Hz, 1H), 3.94 (s, 3H),3.20 (s, 3H), 2.42 (s, 3H); ^{13}C NMR (100 MHz, $CDCl_3$): δ = 167.1, 164.1, 152.2, 136.5, 135.0, 129.6 (2C), 129.2 (2C), 128.3 (2C), 127.9 , 126.9, 124.4 (2C), 109.54, 52.1, 35.8, 12.3; IR (KBr): 3064, 3028, 1716, 1651, 1608, 1588, 1453, 1376, 1344, 1056, 962, 890, 861, 760, 709, 699 cm^{-1}; HR-MS: m/z calcd for $C_{19}H_{18}N_2O_3$: 322.1324$[M]^+$; found: 322.1319.

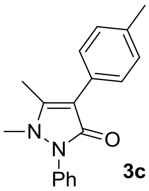

3c

1,5-Dimethyl-2-phenyl-4-(*p*-tolyl)-1*H*-pyrazol-3(2*H*)-one (**3c**):

white solid. mp 148.9-149.7 °C. ^{1}H NMR (400 MHz, $CDCl_3$): δ = 7.49-7.48 (m, 4H), 7.46 (d, *J* = 8.0 Hz, 2H), 7.31 (t, *J* = 3.6 Hz, 1H), 7.24 (d, *J* = 8.0 Hz, 2H), 3.15 (s, 3H), 2.38 (s, 6H). ^{13}C NMR (100 MHz, $CDCl_3$) δ= 164.7, 152.2 , 136.4, 135.5, 129.1 (2C), 129.0 (2C), 128.8 (2C), 128.3, 126.3, 123.9 (2C), 111.4, 36.2, 21.3, 12.2. IR (KBr): 3100, 3056, 1650, 1590, 1510, 1494, 1456, 1372,

1345, 1056, 912, 889, 827, 782, 695 cm^{-1} ; HR-MS: m/z calcd for $C_{18}H_{18}N_2O$: 278.1422 $[M]^+$; found: 278.1428.

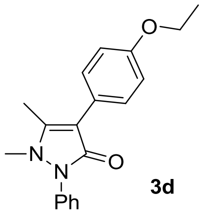

3d

4-(4-Ethoxyphenyl)-1,5-dimethyl-2-phenyl-1*H*-pyrazol-3(2*H*)-one (**3d**):

light yellow solid. mp: 158.2-158.9 °C. ^{1}H NMR (400 MHz, $CDCl_3$): δ = 7.49-7.48 (m, 6H), 7.32-7.29 (m, 1H), 6.96 (d, *J* = 8.4 Hz, 2H), 4.07 (q, *J* = 6.8 Hz, 2H), 3.14 (s, 3H), 2.38. (s, 3H), 1.4(t, *J* = 6.8 Hz, 3H); ^{13}C NMR (100 MHz, $CDCl_3$): δ = 164.8, 157.8, 152.0, 135.5, 130.5 (2C) 129.1 (2C), 126.3, 123.8 (2C), 123.4, 114.5 (2C), 111.3, 63.4, 36.2, 14.9, 12.2; IR (KBr): 3132, 3066, 1651, 1613, 1558, 1533, 1491, 1435, 1389, 1344, 971, 936, 850, 782, 713 cm^{-1}; HR-MS: m/z calcd for $C_{19}H_{20}N_2O_2$: 308.1532 $[M]^+$; found: 308.1527

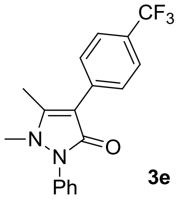

3e

1,5-Dimethyl-2-phenyl-4-(4-(trifluoromethyl)phenyl)-1*H*-pyrazol-3(2*H*)-one (**3e**):

white solid. mp: 162.7-163.2 °C. ^{1}H NMR (400 MHz, $CDCl_3$): δ = 7.72-7.66 (m, 4H), 7.52-7.45 (m, 4H), 7.34 (t, *J* = 6.8 Hz, 1H), 3.21 (s, 3H), 2.43 (s, 3H); ^{13}C NMR(100 MHz,$CDCl_3$): δ = 164.1, 152.1, 135.2, 134.9, 129.2 (2C), 128.8 (2C), 128.4 (q, *J* = 64.1 Hz, 1C), 126.9 (2C), 125.2 (q, *J* = 5.7 Hz , 2C), 124.4, 124.3(q, *J* =270.1 Hz, 1C), 109.3, 36.7, 12.2; IR (KBr): 3071, 3024, 1646, 1616, 1591, 1496, 1458, 1404, 1376, 1324, 914, 855, 805, 751, 698 cm^{-1}; HR-MS: m/z calcd for $C_{18}H_{15}F_3N_2O$: 332.1145 $[M]^+$; found: 332.1138.

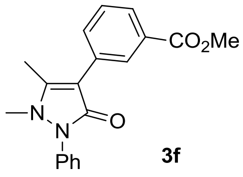

3f

Methyl 3-(1,5-dimethyl-3-oxo-2-phenyl-2,3-dihydro-1*H*-pyrazol-4-yl)benzoate (**3f**):

yellow solid. mp: 149.5-150.4 °C; ^{1}H NMR (400 MHz, $CDCl_3$): δ = 8.20 (s, 1H), 7.97 (d, *J* = 8.0 Hz, 1H), 7.84 (d, *J* = 8.0 Hz, 1H), 7.52-7.47 (m , 5H), 7.34-7.29 (m, 1H), 3.93 (s, 3H), 3.19(s, 3H), 2.42 (s, 3H); ^{13}C NMR (100 MHz, $CDCl_3$): δ = 167.2, 164.4, 152.4, 135.2, 133.4, 131.8, 130.2, 129.7, 129.2 (2C), 128.5, 127.8, 126.6, 124.2 (2C), 110.0, 52.1, 35.9, 12.2; IR (KBr): 3060, 3027, 2945, 1723, 1649, 1581, 1488, 1414, 1325, 1306, 964, 879, 763, 694 cm^{-1}; HR-MS: m/z calcd for $C_{19}H_{18}F_3N_2O_3$: 322.1319 $[M]^+$; found: 322.1322.

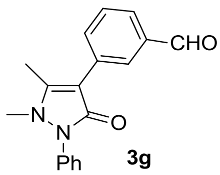

3g

3-(1,5-Dimethyl-3-oxo-2-phenyl-2,3-dihydro-1*H*-pyrazol-4-yl)benzaldehyde (3g):

yellow soild, mp: 179.1-180.6 ^oC. ^1H NMR (400 MHz, $CDCl_3$): δ = 10.07 (s, 1H), 8.09 (s, 1H), 7.91(d, *J* = 7.6 Hz, 1H), 7.82 (d, *J* = 7.6 Hz, 1H), 7.59(t, *J* = 8.0 Hz, 1H), 7.53-7.46 (m, 4H), 7.34 (t, *J* = 7.2 Hz, 1H), 3.22 (s, 3H), 2.45 (s, 3H). ^{13}C NMR (100 MHz, $CDCl_3$): δ = 192.5, 164.3, 152.2, 136.5, 134.9, 134.7, 132.6, 130.3, 129.2 (2C), 129.1, 127.5, 126.9, 124.3 (2C), 103.3, 35.8, 12.2; IR (KBr): 3088, 3013, 1787, 1764, 1692, 1658, 1649, 1582, 1565, 1529, 1432, 945, 887, 763, 683 cm^{-1}; HR-MS: m/z calcd for $C_{18}H_{16}N_2O_2$: 292.1216 $[M]^+$; found: 292.1221

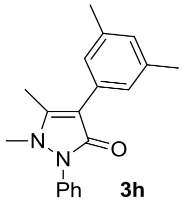

3h

4-(3,5-Dimethylphenyl)-1,5-dimethyl-2-phenyl-1*H*-pyrazol-3(2*H*)-one (3h):

yellow solid. mp: 207.0-207.8^oC. ^1H NMR (400 MHz, $CDCl_3$): δ = 7.49-7.48 (m, 4H), 7.32-7.29 (m, 1H), 7.18 (s, 2H), 6.96 (s, 1H), 3.14 (s, 3H), 2.38 (s, 3H), 2.36 (s, 6H); ^{13}C NMR (100 MHz, $CDCl_3$): δ = 164.7, 152.5, 137.7 (2C), 135.5, 131.0, 129.0 (2C), 128.5, 126.7 (2C), 126.3, 123.8 (2C), 111.5, 36.2, 21.4 (2C), 12.2; IR (KBr): 3084, 3053, 3013, 1655, 1620, 1592, 1468, 1455, 1373, 1323, 880, 855, 758, 700 cm^{-1}; HR-MS: m/z calcd for $C_{19}H_{20}N_2O$: 292.1566 $[M]^+$; found: 292.1558

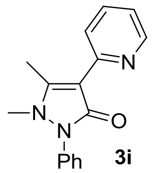

3i

1,5-Dimethyl-2-phenyl-4-(pyridin-2-yl)-1*H*-pyrazol-3(2*H*)-one (**3i**):

yellow solid. mp: 101.0-101.9 ^oC. ^1H NMR (400 MHz, $CDCl_3$): δ = 8.59 (d, *J* = 4.4 Hz, 1H), 8.31 (d, *J* = 8.0 Hz, 1H), 7.72-7.69 (m, 1H), 7.53-7.43 (m, 4H), 7.35 (t, *J* = 7.2 Hz, 1H), 7.13-7.10(m, 1H) 3.25(s, 3H), 2.78(s, 3H); ^{13}C NMR (100 MHz, $CDCl_3$): δ = 164.6, 154.5, 152.4, 148.5, 136.2, 135.0, 129.2 (2C), 127.1, 124.9 (2C), 122.5, 120.7, 107.5, 35.1, 12.8; IR (KBr): 3150, 3027, 1754, 1737, 1581, 1529, 1493, 1468, 1366, 1330, 879, 752, 712 cm^{-1} ; HR-MS: m/z calcd for $C_{16}H_{15}N_3O$: 265.1219 $[M]^+$; found: 265.1216

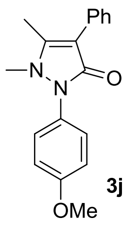

3j

2-(4-Methoxyphenyl)-1,5-dimethyl-4-phenyl-1*H*-pyrazol-3(2*H*)-one (**3j**):

tan solid. mp: 134.4-135.1 °C. ^{1}H NMR (400 MHz, $CDCl_3$): δ = 7.57 (d, *J* = 7.2 Hz, 2H), 7.42-7.37 (m, 4H), 7.31 (s, 1H), 7.03-7.02 (m, 2H), 3.86 (s , 3H), 3.18 (s, 3H), 2.39 (s, 3H); ^{13}C NMR (100 MHz, $CDCl_3$): δ = 164.6, 158.5, 150.9, 131.5, 128.8 (2C), 128.3 (2C), 128.1, 126.6, 126.5 (2C), 114.5 (2C), 110.6, 55.6, 35.5, 12.1; IR (KBr): 3060, 3035, 1660, 1622, 1592, 1510, 1454, 1335, 836, 807, 704, 670cm^{-1} ; HR-MS: m/z calcd for $C_{18}H_{18}N_2O_2$: 294.1365 $[M]^+$; found: 294.1372

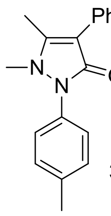

3k

1,5-Dimethyl-4-phenyl-2-(*p*-tolyl)-1*H*-pyrazol-3(2*H*)-one (**3k**):

yellow solid. mp: 157.3-158.2 °C. ^{1}H NMR (400 MHz, $CDCl_3$): δ = 7.57 (d, *J* = 7.2 Hz, 2H), 7.42 (t, *J* = 7.6 Hz, 2H), 7.35 (d, *J* = 8.0 Hz, 2H), 7.30-7.28 (m, 3H), 3.15 (s, 3H), 2.40 (s, 3H), 2.39 (s, 3H); ^{13}C NMR (100 MHz, $CDCl_3$): δ = 164.5, 151.6, 136.5, 132.7, 131.4, 129.7 (2C), 128.9 (2C), 128.3 (2C), 126.6, 124.3 (2C), 110.9, 35.8, 21.1, 12.1; IR (KBr): 3078, 3048, 1654, 1609, 1511, 1487, 1418, 1303, 935, 881, 803, 778, 741, 669 cm^{-1} ; HR-MS: m/z calcd for $C_{18}H_{18}N_2O$: 278.1432 $[M]^+$; found: 278.1439.

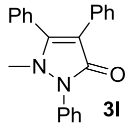

3l

1-Methyl-2,4,5-triphenyl-1*H*-pyrazol-3(2*H*)-one (**3l**) :

yellow solid. mp: 108.8-109.7 °C. ^{1}H NMR (400 MHz, $CDCl_3$): δ = 7.64 (d, *J* = 7.2 Hz, 2H) ,7.50 (t, *J* = 8.0 Hz, 3H) ,7.48-7.44 (m, 6H), 7.32 (t, *J* = 7.2 Hz, 1H), 7.26 (t, *J* = 7.6 Hz, 2H), 7.20 (t, *J* = 7.2 Hz, 1H), 2.97 (s, 3H); ^{13}C NMR (100 MHz, $CDCl_3$): δ = 164.2, 155.9, 135.4, 130.6, 130.2, 129.6 (2C), 129.5, 129.2(2C), 129.1 (2C), 128.9 (2C), 128.1 (2C), 126.7, 126.4, 123.6 (2C), 112.2, 38.7; IR (KBr): 3056, 2924, 1667, 1592, 1494, 1447, 1393, 1342, 911, 879, 803, 755, 695 cm^{-1} ; HR-MS: m/z calcd for $C_{22}H_{18}N_2O$: 326.1425 $[M]^+$; found: 326.1418.

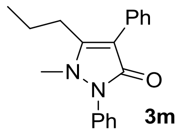

1-Methyl-2,4-diphenyl-5-propyl-1*H*-pyrazol-3(2*H*)-one (3m):

yellow solid. mp: 116.5-117.2 ^{o}C . ^{1}H NMR (400 MHz, $CDCl_3$): δ = 7.53 (d, *J* = 7.2 Hz, 2H), 7.49 (d, *J* = 4.4 Hz, 4H), 7.42(t, *J* = 7.6 Hz, 2H), 7.32-7.29(m, 2H), 3.15 (s, 3H), 2.71 (t, *J* = 7.6 Hz, 2H), 1.77(q, *J* = 7.6 Hz, 2H), 1.07(t, *J* = 7.2 Hz, 3H); ^{13}C NMR (100 MHz, $CDCl_3$): δ = 164.8, 156.6, 135.4, 131.3, 129.1 (2C), 129.0 (2C), 128.4 (2C), 126.8, 126.4, 123.9 (2C), 111.6, 36.3, 27.4, 22.4, 14.0; IR (KBr): 3076, 3012, 1637, 1615, 1572, 1478, 1384, 1011, 972, 879, 724, 687 cm^{-1} ; HR-MS: m/z calcd for $C_{19}H_{20}N_2O$: 292.1627 $[M]^+$; found: 292.1622.

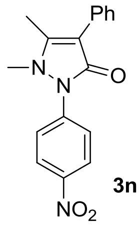

1,5-Dimethyl-2-(4-nitrophenyl)-4-phenyl-1*H*-pyrazol-3(2*H*)-one (3n) :

yellow solid. mp: 165.5-166.3 ^{o}C. ^{1}H NMR (400 MHz, $CDCl_3$): δ = 8.37 (d, *J* = 9.2 Hz, 2H), 7.72 (d, *J* = 9.2, 2H), 7.53-7.51 (m, 2H), 7.45 (t, *J* = 7.6, 2H), 7.34 (t, *J* = 7.5 Hz, 1H), 3.19 (s, 3H), 2.40 (s, 3H); ^{13}C NMR (100 MHz, $CDCl_3$): δ = 164.9, 155.8, 144.7, 144.1, 130.2, 128.9 (2C), 128.5 (2C), 127.4, 124.8 (2C), 122.1 (2C), 122.7, 37.3, 12.4; IR (KBr): 3111, 3077, 1661, 1639, 1616, 1588, 1514, 1491, 1412, 1339, 1054, 859, 748, 706 cm^{-1}; HR-MS: m/z calcd for $C_{17}H_{15}N_3O_3$: 309.1089 $[M]^+$; found: 309.1096.

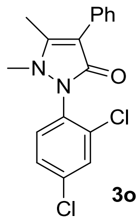

2-(2,4-Dichlorophenyl)-1,5-dimethyl-4-phenyl-1*H*-pyrazol-3(2*H*)-one (3o):

yellow solid.mp: 179.8-180.5 ^{o}C; ^{1}H NMR (400 MHz, $CDCl_3$): δ = 7.58-7.57(m, 3H), 7.42 (t, *J* = 7.7 Hz, 2H), 7.38 (d, *J* = 2.1 Hz, 1H), 7.35 (d, *J* = 8.4 Hz, 1H), 7.32-7.25 (m, 1H), 3.12 (s, 3H), 2.40 (s, 3H); ^{13}C NMR (100 MHz, $CDCl_3$): δ = 164.7, 151.2, 135.6, 134.8, 131.9, 131.3, 131.2, 130.7, 128.8 (2C), 128.4 (2C), 128.1, 126.7, 109.6, 34.8, 12.1; IR (KBr): 3083, 3027, 1661, 1607, 1579, 1474, 1415, 1388, 1307, 837, 821, 759, 702, 667 cm^{-1}; HR-MS: m/z calcd for $C_{17}H_{14}{}^{35}Cl_2N_2O$: 332.0489 $[M]^+$; found: 332.0478.

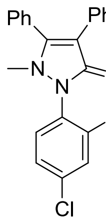

3p

2-(2,4-Dichlorophenyl)-1-methyl-4,5-diphenyl-1*H*-pyrazol-3(2*H*)-one (3p):

yellow solid. mp: 149.8-150.7 °C. ^{1}H NMR (400 MHz, $CDCl_3$): δ = 7.62 (d, *J* = 2.1 Hz, 1H), 7.53 -7.39 (m, 9H), 7.25 (t, *J* = 7.3 Hz, 2H), 7.18 (t, *J* = 7.2 Hz, 1H), 2.92 (s, 3H); ^{13}C NMR (100 MHz, $CDCl_3$): δ = 164.4, 155.3, 135.6, 134.4, 131.8, 131.0, 130.8, 130.5, 130.3, 129.6 (2C), 129.3 , 129.2 (2C), 128.8 (2C), 128.1 (3C), 126.6, 110.6, 37.2; IR (KBr): 3062, 3031, 1667, 1639, 1581, 1480, 1441, 1415, 1396, 1315, 1022, 924, 880, 825, 783, 716 cm^{-1};HR-MS: m/z calcd for $C_{22}H_{16}{}^{35}Cl_2N_2O$: 394.0638 $[M]^+$; found: 394.0631.

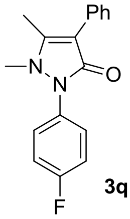

3q

2-(4-Fluorophenyl)-1,5-dimethyl-4-phenyl-1*H*-pyrazol-3(2*H*)-one (3q):

yellow solid. mp: 149.3-150.3 °C. ^{1}H NMR (400 MHz, $CDCl_3$): δ = 7.56 (d, *J* =7.3 Hz, 2H), 7.44 (m, 4H), 7.31 (d, *J* = 7.4 Hz, 1H), 7.19 (t, *J* = 8.6 Hz, 2H), 3.15 (s, 3H), 2.40 (s, 3H); ^{13}C NMR (100 MHz, $CDCl_3$): δ = 164.8, 161.1(d, *J* = 246.4 Hz, 1C), 152.5, 131.4, 131.1, 128.9 (2C), 128.4 (2C), 126.8, 125.9 (d, *J* = 8.5 Hz, 2C), 116.1 (d, *J* = 22.7 Hz, 2C), 111.3, 36.0, 12.2; IR (KBr): 3065, 3021, 1654, 1603, 1503, 1438, 1340, 1307, 1011, 881, 786, 763, 702 cm^{-1}; HR-MS: m/z calcd for $C_{17}H_{15}{}^{19}F_2N_2O$: 282.1174 $[M]^+$; found: 282.1171.

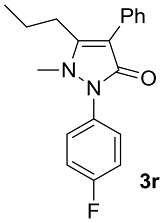

3r

2-(4-Fluorophenyl)-1-methyl-4-phenyl-5-propyl-1*H*-pyrazol-3(2*H*)-one (3r):

yellow solid. mp: 106.2-107.1 °C. ^{1}H NMR (400 MHz, $CDCl_3$): δ = 7.51 (d, *J* = 7.4 Hz, 2H), 7.48-7.39 (m, 4H), 7.32 (d, *J* = 7.3 Hz, 1H), 7.18 (t, *J* = 8.6 Hz, 2H), 3.13 (s, 3H), 2.71 (t, *J* = 8.0 Hz, 2H), 1.76 (m, 2H), 1.07 (t, *J* = 7.2 Hz, 3H); ^{13}C NMR (100 MHz, $CDCl_3$): δ = 165.0, 161.0 (d, *J* = 246.3 Hz, 1C), 156.8, 131.5, 131.2, 129.0 (2C), 128.4 (2C), 126.9, 125.8 (d, *J* = 8.4 Hz, 2C), 116.0 (d, *J* = 22.8 Hz, 2C), 111.6, 36.2, 27.4, 22.4, 14.03; IR (KBr): 3051, 3014, 1649, 1603, 1582, 1457, 1414, 1322, 1017, 913, 837, 826, 743, 702 cm^{-1}; HR-MS: m/z calcd for $C_{19}H_{19}{}^{19}FN_2O$: 310.1472 $[M]^+$; found: 310.1481.

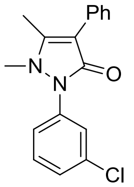
3s

2-(3-Chlorophenyl)-1,5-dimethyl-4-phenyl-1*H*-pyrazol-3(2*H*)-one (3s):

yellow solid. mp：203.6-204.3 °C. ^{1}H NMR (400 MHz, $CDCl_3$): δ = 7.54 (d, *J* = 7.4 Hz, 2H), 7.51 (s, 1H), 7.45-7.41(m, 4H), 7.33-7.26 (m, 2H), 3.19 (s, 3H), 2.40 (s, 3H); ^{13}C NMR (100 MHz, $CDCl_3$): δ = 164.8, 153.7, 136.7, 134.8, 130.9, 130.1, 128.9 (2C), 128.4 (2C), 127.0, 126.3, 123.4, 121.6, 111.7, 36.5, 12.2; IR (KBr): 3071, 3028, 1764, 1662, 1629, 1586, 1549, 1462, 1310, 1022, 981, 917, 861, 786, 702 cm^{-1}; HR-MS: m/z calcd for $C_{17}H_{15}{}^{35}ClN_2O$: 298.0865 $[M]^+$; found: 298.0874.

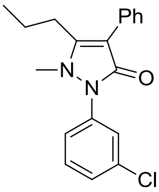
3t

2-(3-chlorophenyl)-1-methyl-4-phenyl-5-propyl-1*H*-pyrazol-3(2*H*)-one (3t):

yellow solid. mp:115.5-116.2 °C. ^{1}H NMR (400 MHz, $CDCl_3$): δ = 7.51-7.49 (m, 3H), 7.44-7.41 (m, 4H), 7.32 (t, J = 7.3 Hz, 1H), 7.29-7.25 (m, 1H), 3.14 (s, 3H), 2.73-2.68 (t, *J* = 7.8 Hz, 2H), 1.80-1.74 (m, 2H), 1.07 (t, *J* = 7.4 Hz, 3H); ^{13}C NMR (100 MHz, $CDCl_3$): δ = 164.9, 157.9, 136.7, 134.7, 130.9, 130.1, 129.0 (2C), 128.4 (2C), 127.0, 126.2, 123.3, 121.6, 112.1, 36.7, 27.4, 22.4, 14.1. IR (KBr): 3129, 3080, 1657, 1612, 1590, 1481, 1429, 1399, 1342, 1018, 998, 860, 829, 774, 725, 697 cm^{-1}; HR-MS: m/z calcd for $C_{19}H_{19}{}^{35}ClN_2O$: 326.1174 $[M]^+$; found: 326.1182.

References

1. Balestra, M.; Bunting, H.; Chen, D.; Egle, I.; Forst, J.; Frey, J.; Isaac, M.; Ma, F.; Nugiel, D.; Slassi, A.; Steelman, G.; Sun, G.; Sundar, B.; Ukkiramapandian, R.; Urbanek, R.; Walsh, S. Pyrazolone Compounds as Metabotropic Glutamate Receptor Agonists for the Treatment of Neurological and Psychiatric Disorders. WO Patent 2006071730A1, July 6, 2006.
2. Ailawadi, S.; Jyoti, Yadav, M.; Pathak, D. *Der Pharma Chemica* **2011,** *3*, 215–222.

NMR spectra data for all compounds

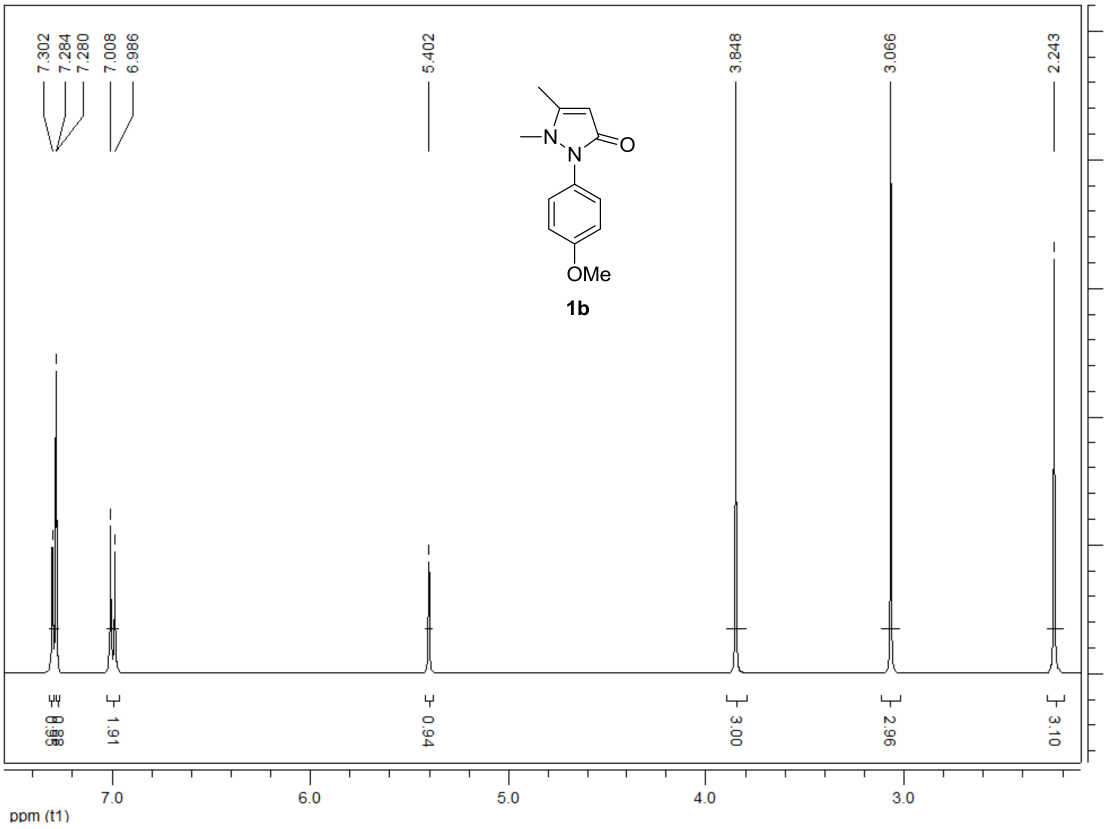

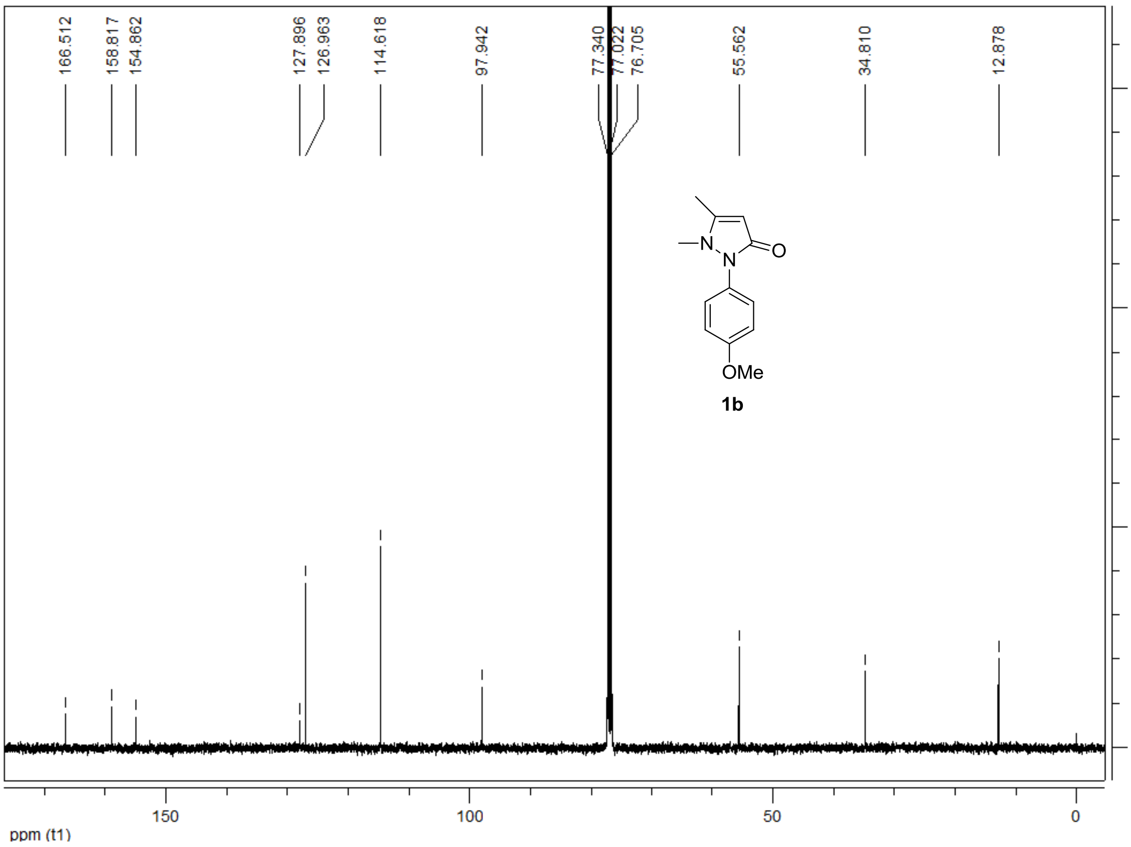

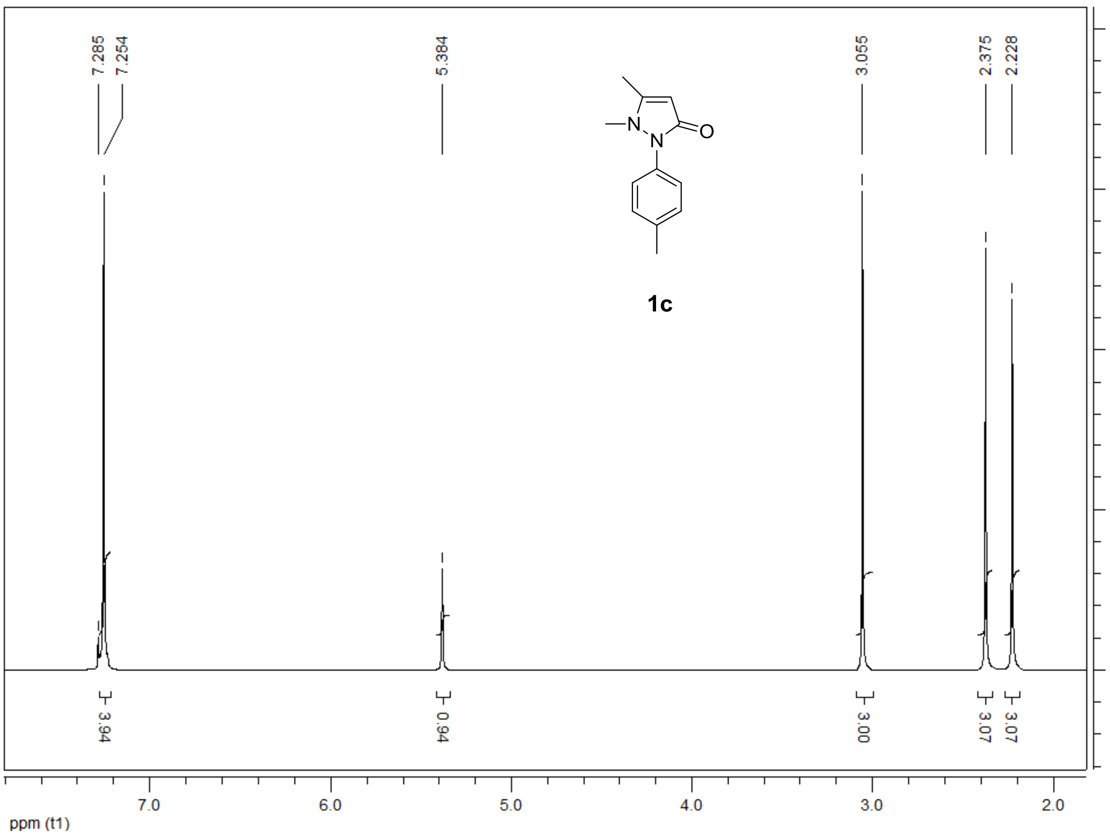
7.285
7.254
5.384
3.055
2.375
2.228
1c
3.94
0.94
3.00
3.07
3.07
7.0
6.0
5.0
4.0
3.0
2.0
ppm (t1)

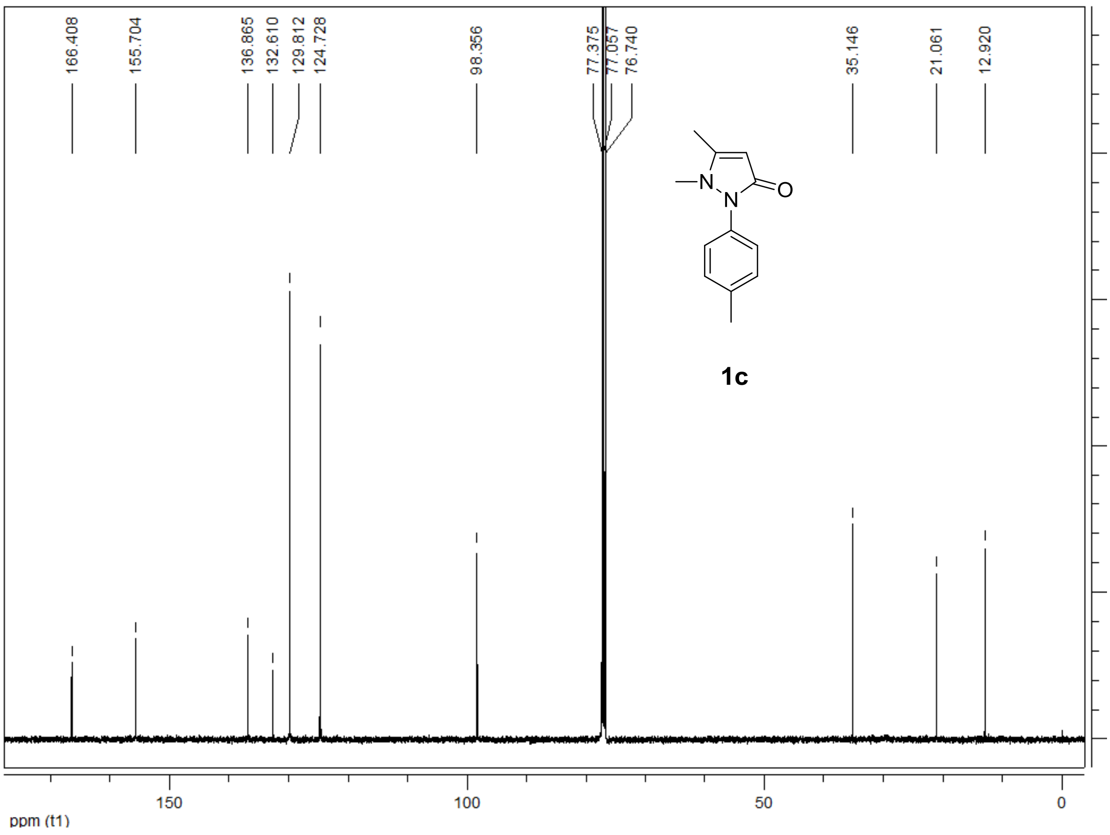
166.408
155.704
136.865
132.610
129.812
124.728
98.356
77.375
77.057
76.740
35.146
21.061
12.920
1c
150
100
50
0
ppm (t1)

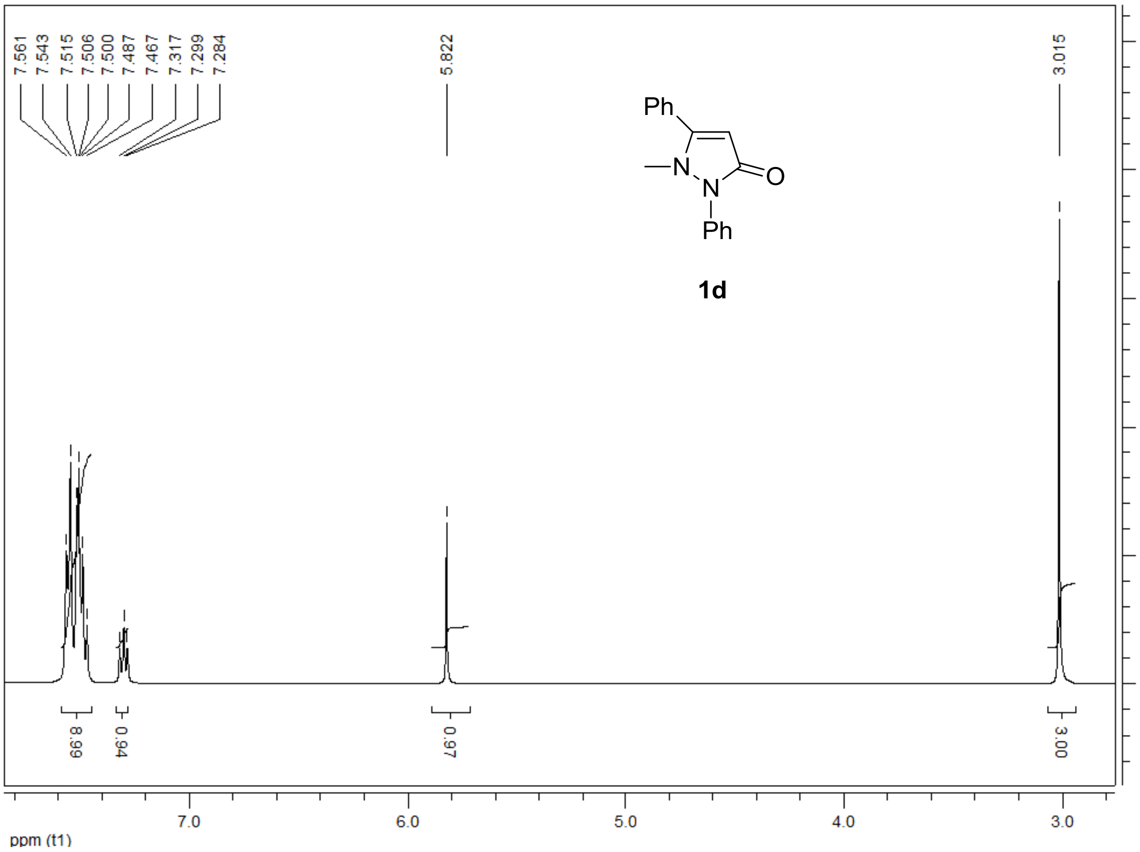
7.561
7.543
7.515
7.506
7.500
7.487
7.467
7.317
7.299
7.284
5.822
3.015
Ph
N
N
O
Ph
1d
8.99
0.94
0.97
3.00
7.0
6.0
5.0
4.0
3.0
ppm (t1)

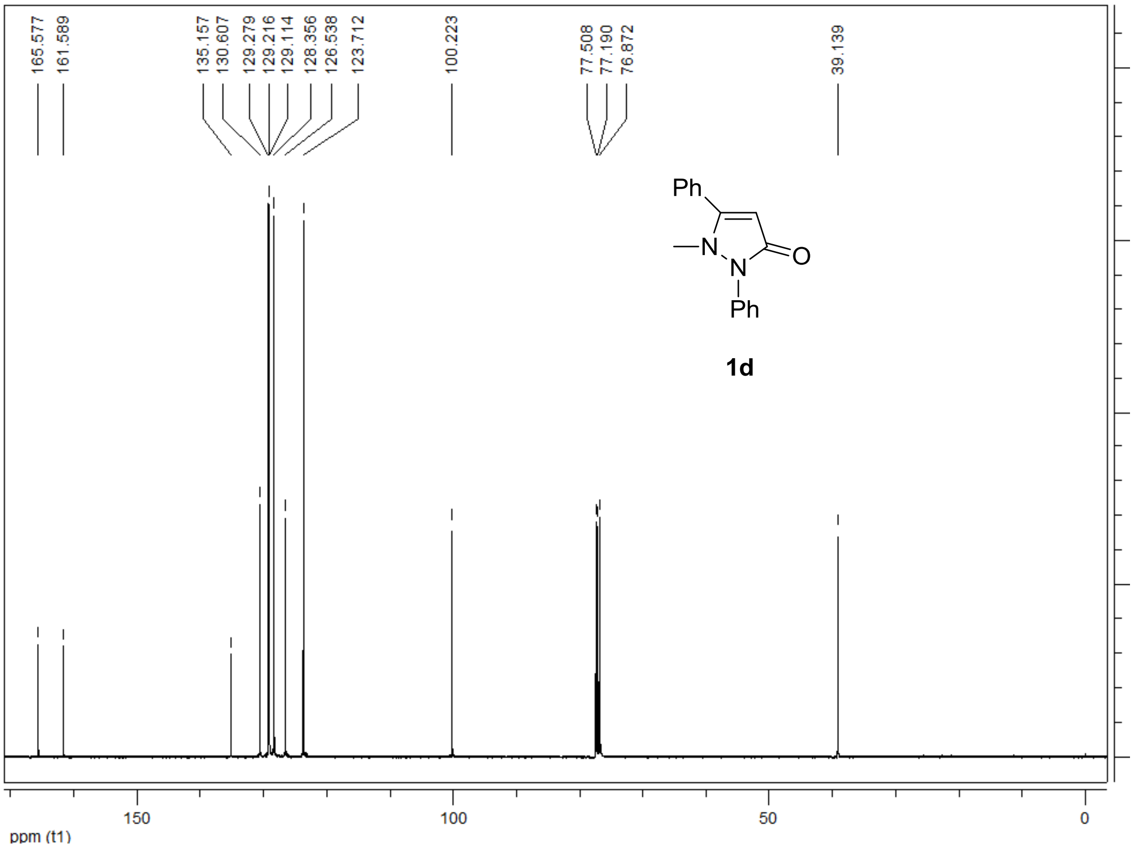
165.577
161.589
135.157
130.607
129.279
129.216
129.114
128.356
126.538
123.712
100.223
77.508
77.190
76.872
39.139
Ph
N
N
O
Ph
1d
150
100
50
0
ppm (t1)

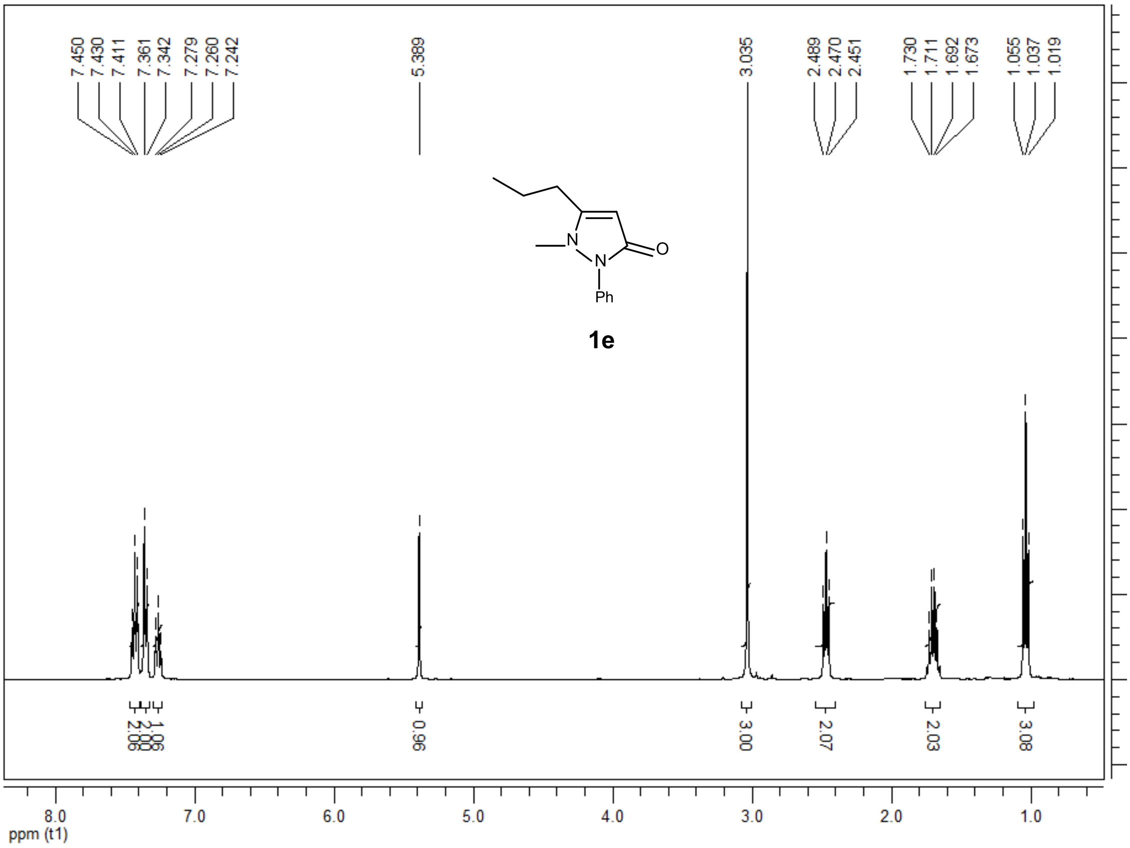

7.450
7.430
7.411
7.361
7.342
7.279
7.260
7.242
5.389
3.035
2.489
2.470
2.451
1.730
1.711
1.692
1.673
1.055
1.037
1.019
N
N
O
Ph
1e
2.06
2.00
1.06
0.96
3.00
2.07
2.03
3.08
8.0
7.0
6.0
5.0
4.0
3.0
2.0
1.0
ppm (t1)

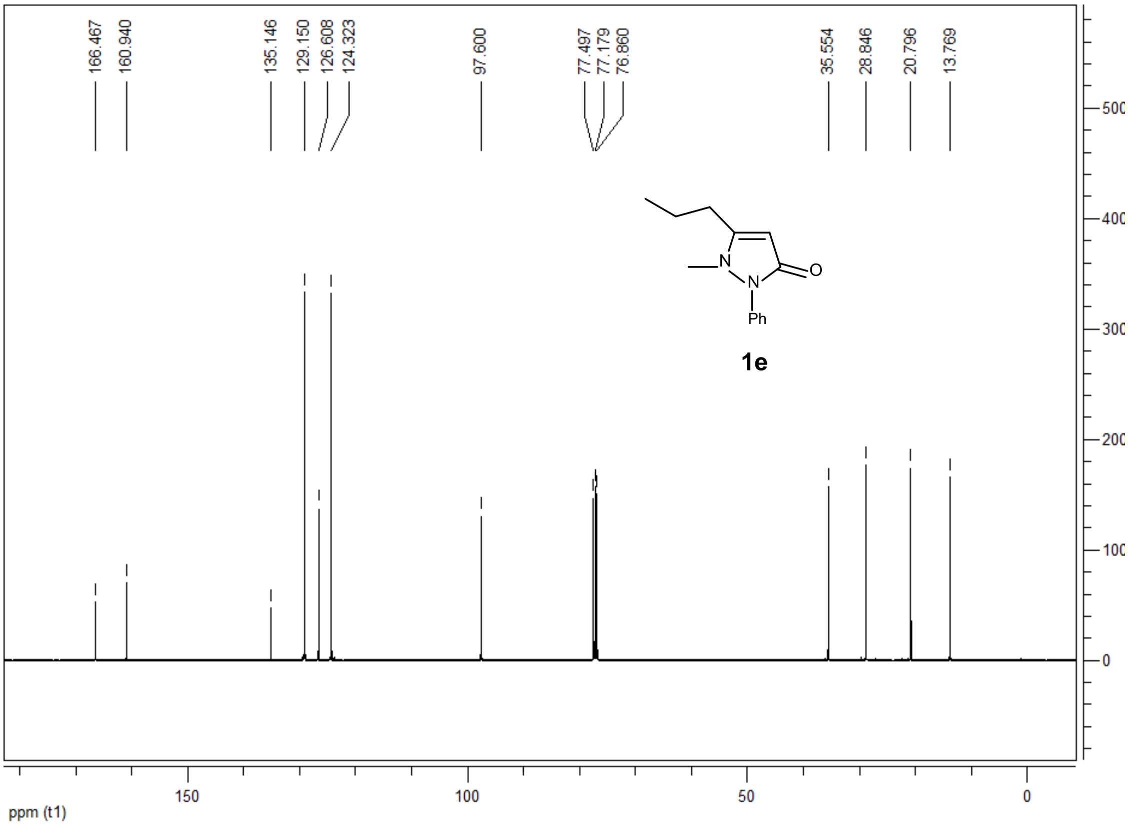

166.467
160.940
135.146
129.150
126.608
124.323
97.600
77.497
77.179
76.860
35.554
28.846
20.796
13.769
N
N
O
Ph
1e
500
400
300
200
100
0
150
100
50
0
ppm (t1)

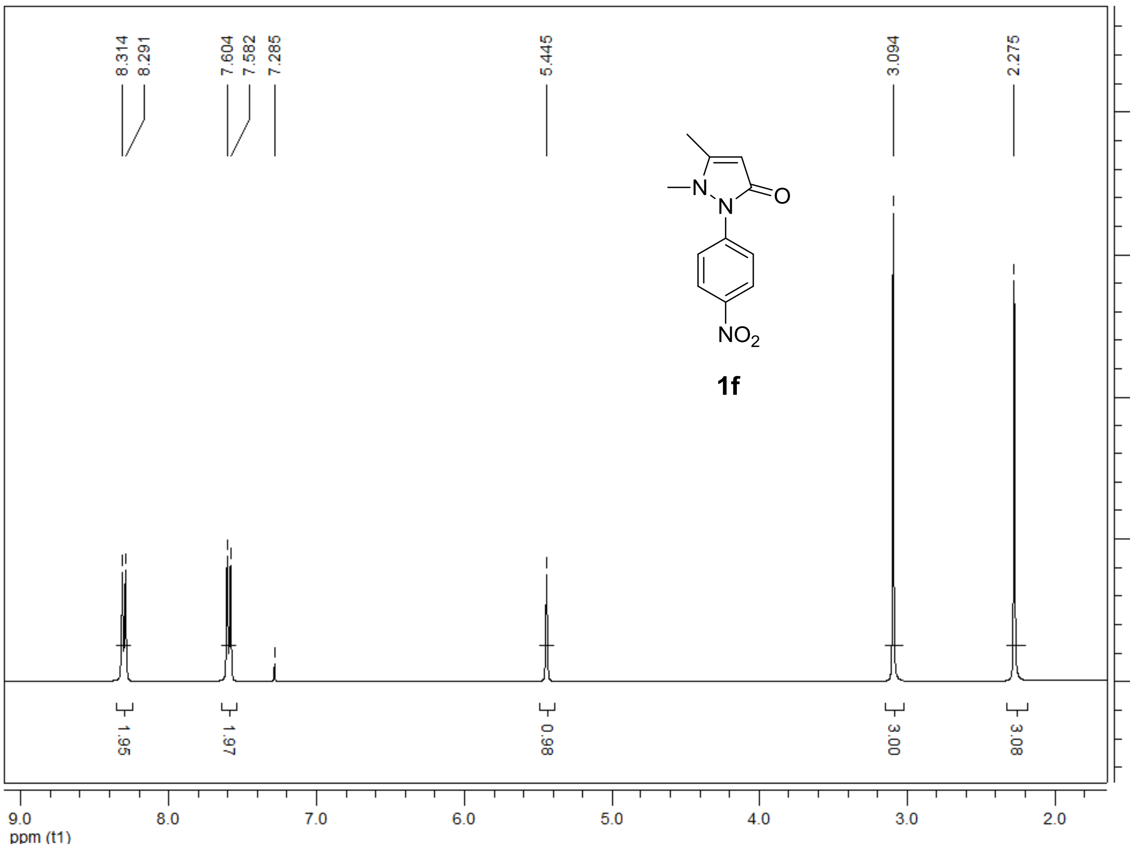
8.314
8.291
7.604
7.582
7.285
5.445
3.094
2.275
N
N
O
NO2
1f
1.95
1.97
0.98
3.00
3.08
9.0
8.0
7.0
6.0
5.0
4.0
3.0
2.0
ppm (t1)

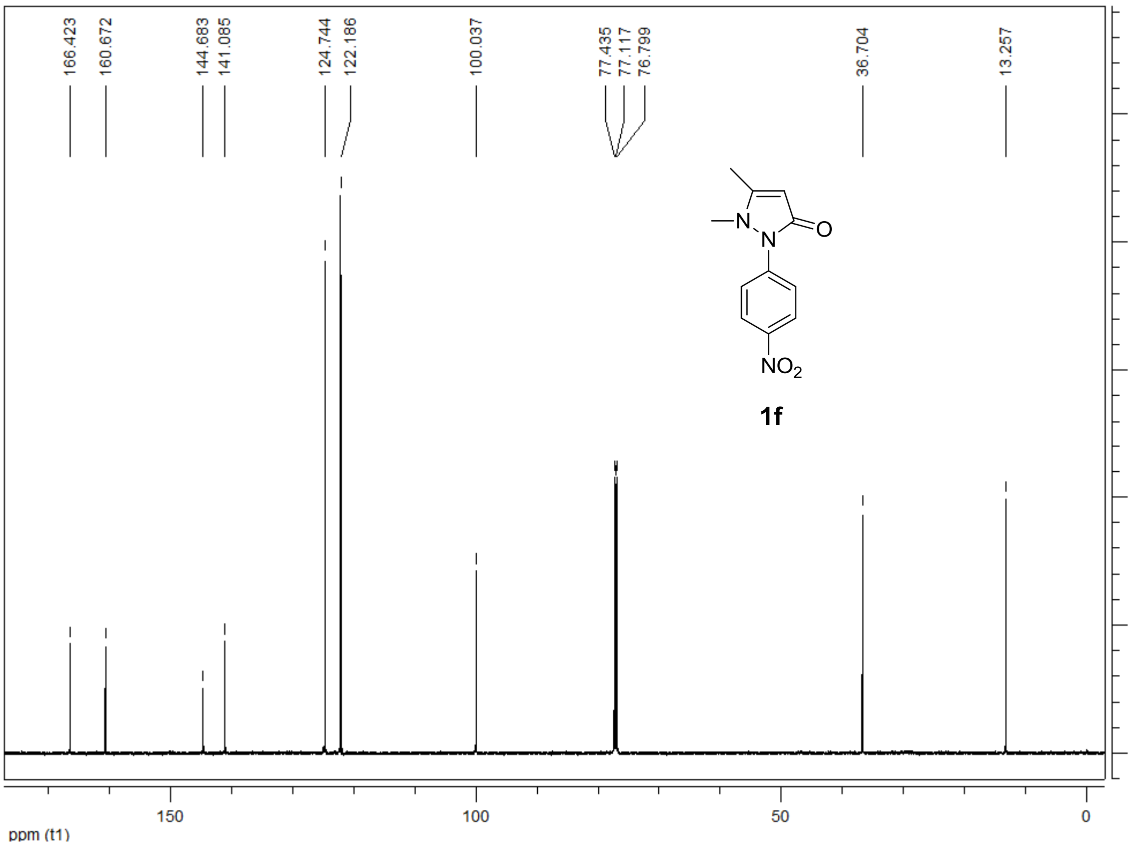
166.423
160.672
144.683
141.085
124.744
122.186
100.037
77.435
77.117
76.799
36.704
13.257
N
N
O
NO2
1f
150
100
50
0
ppm (t1)

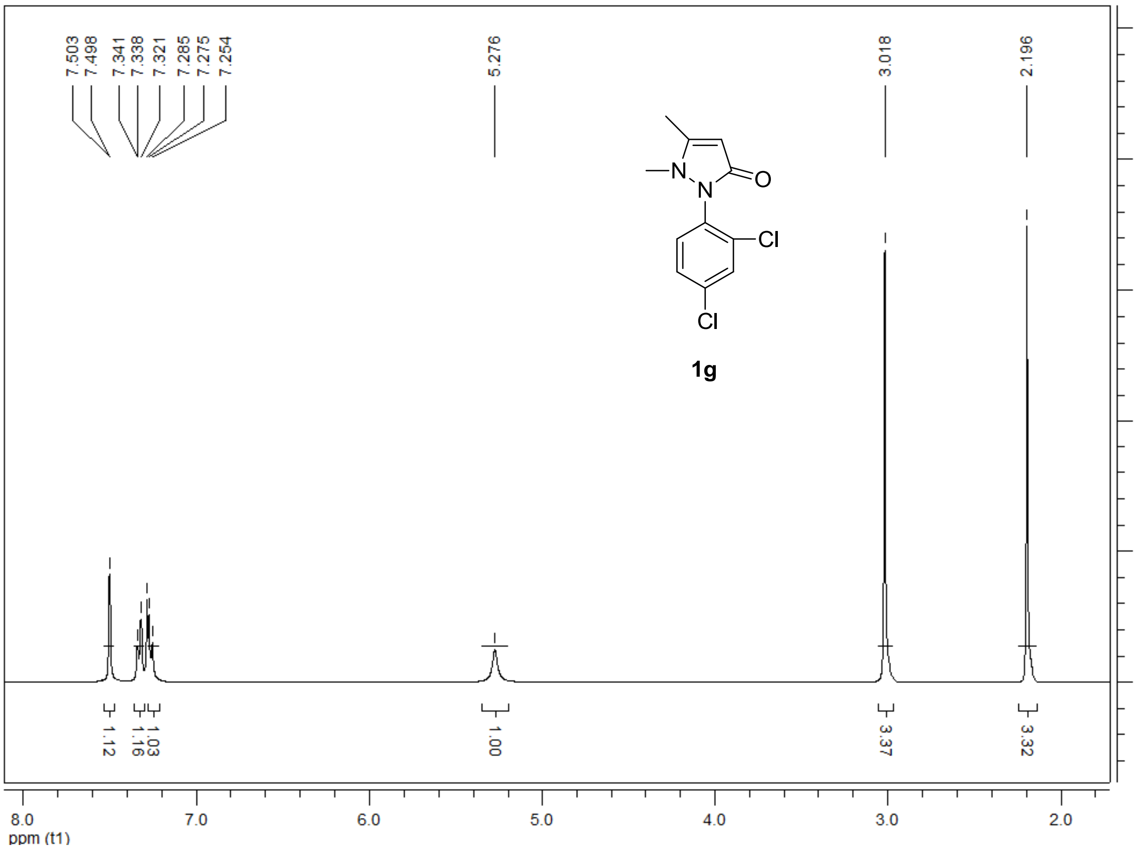
7.503
7.498
7.341
7.338
7.321
7.285
7.275
7.254
5.276
3.018
2.196
N
N
O
Cl
Cl
1g
1.12
1.16
1.03
1.00
3.37
3.32
8.0
7.0
6.0
5.0
4.0
3.0
2.0
ppm (t1)

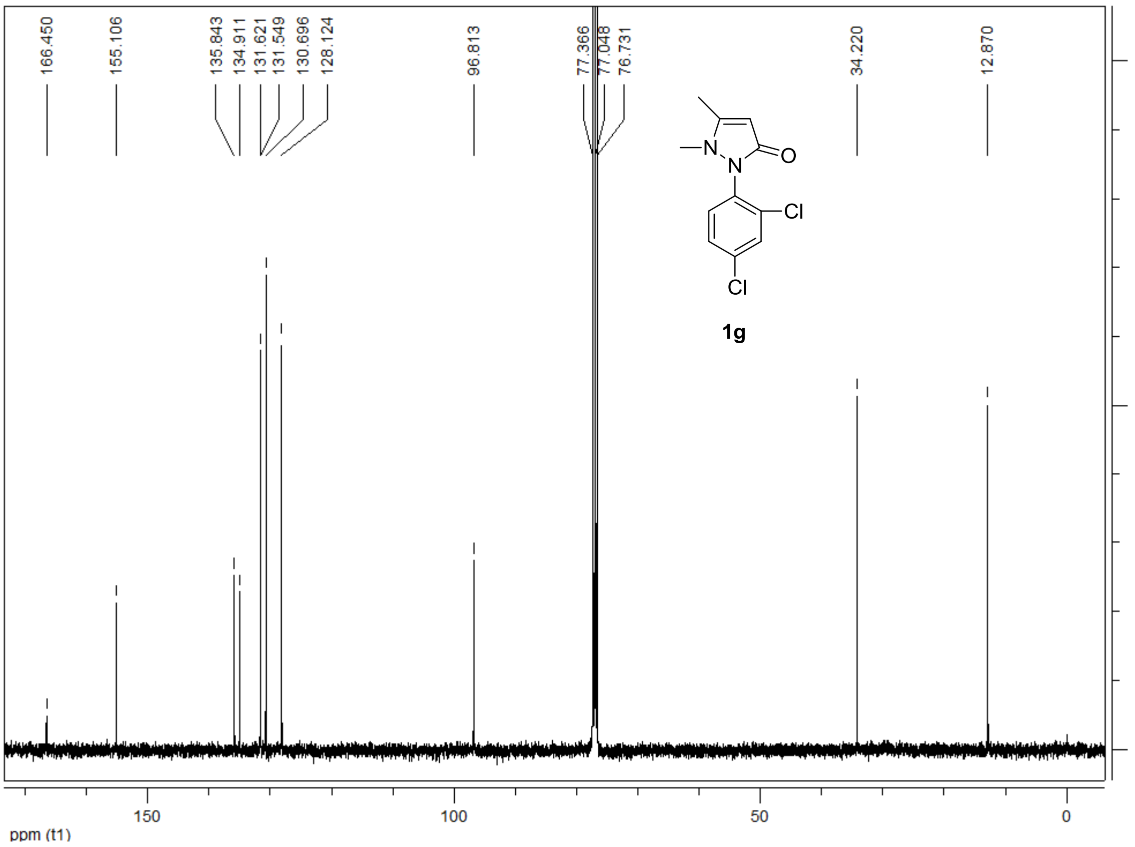
166.450
155.106
135.843
134.911
131.621
131.549
130.696
128.124
96.813
77.366
77.048
76.731
34.220
12.870
N
N
O
Cl
Cl
1g
150
100
50
0
ppm (t1)

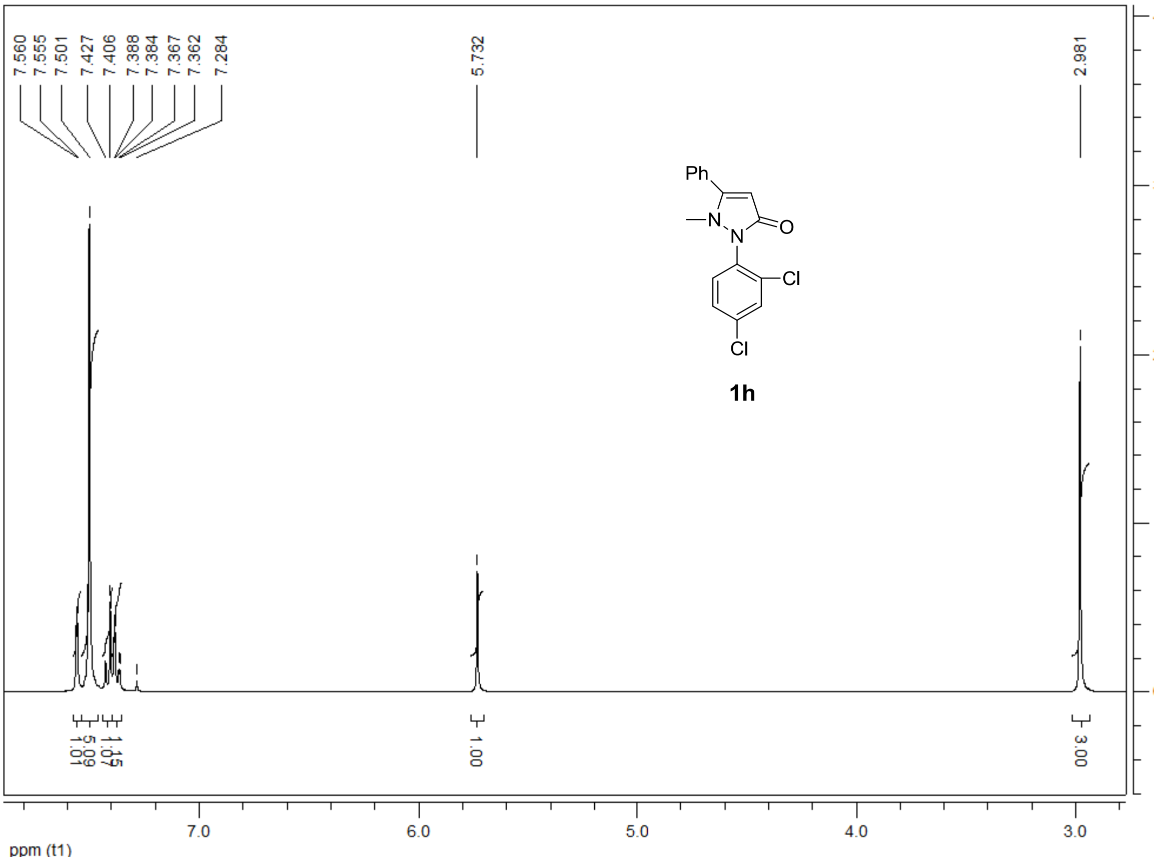
7.560
7.555
7.501
7.427
7.406
7.388
7.384
7.367
7.362
7.284
5.732
2.981
Ph
N
N
O
Cl
Cl
1h
1.01
5.09
1.07
1.15
1.00
3.00
7.0
6.0
5.0
4.0
3.0
ppm (t1)

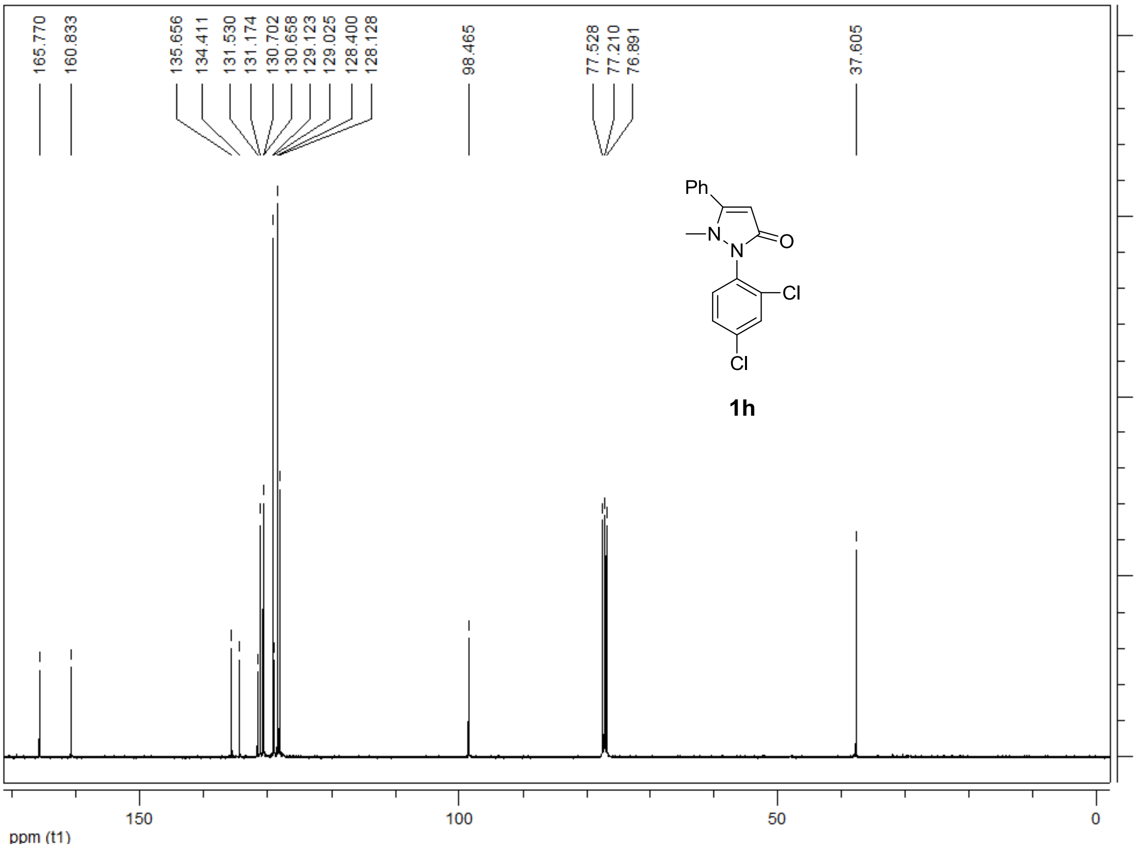
165.770
160.833
135.656
134.411
131.530
131.174
130.702
130.658
129.123
129.025
128.400
128.128
98.465
77.528
77.210
76.891
37.605
Ph
N
N
O
Cl
Cl
1h
150
100
50
0
ppm (t1)

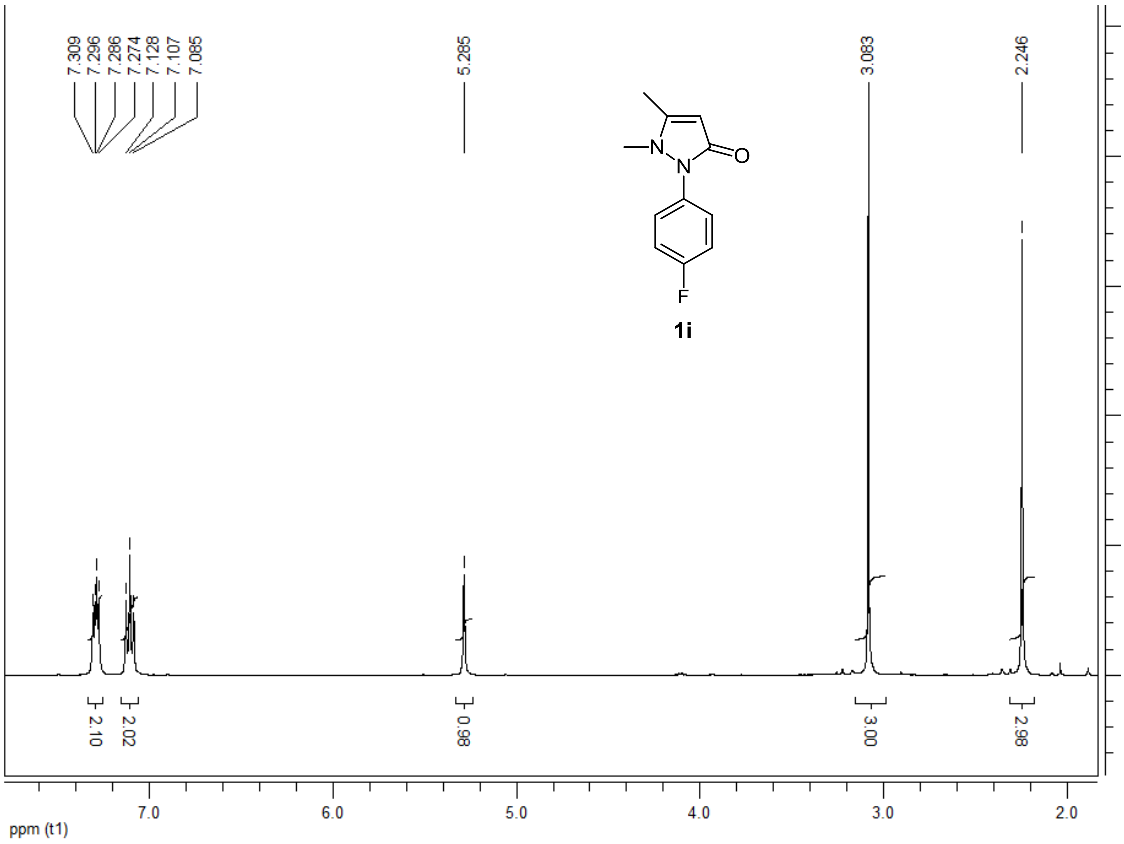
7.309
7.296
7.286
7.274
7.128
7.107
7.085
5.285
3.083
2.246
N
N
O
F
1i
2.10
2.02
0.98
3.00
2.98
7.0
6.0
5.0
4.0
3.0
2.0
ppm (t1)

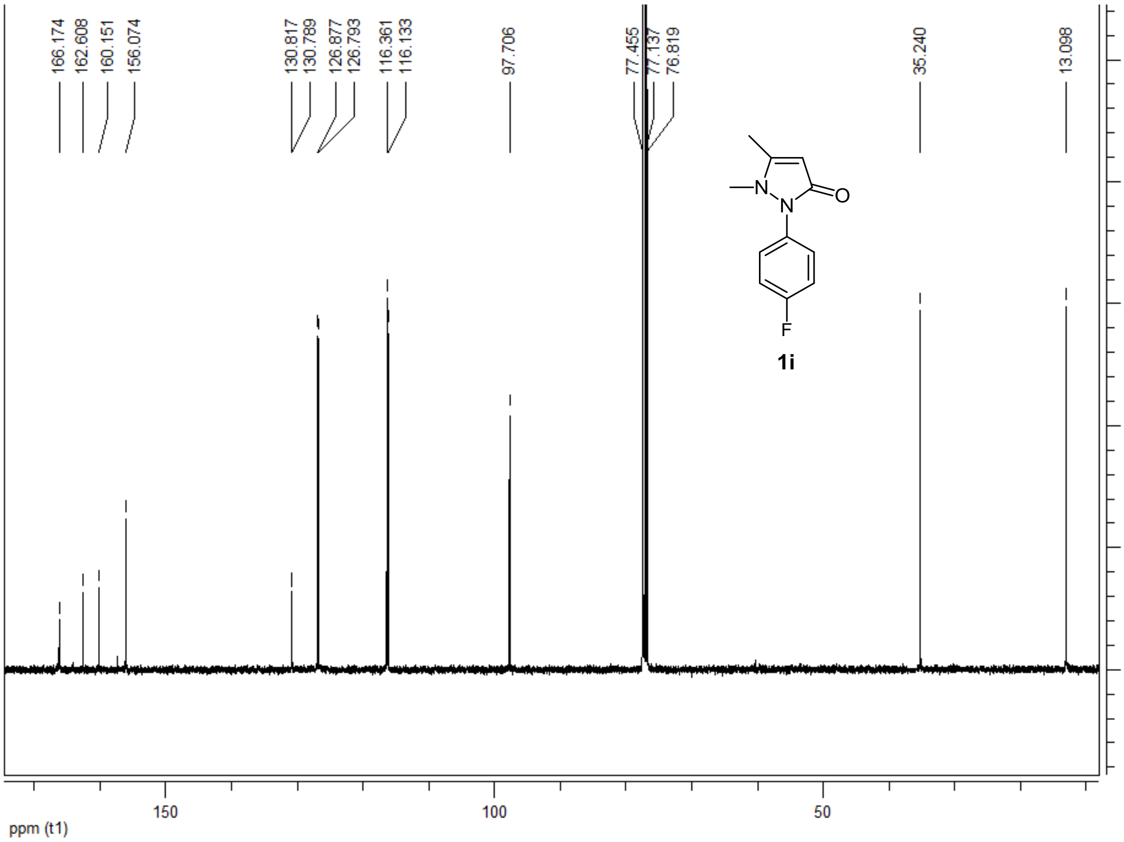
166.174
162.608
160.151
156.074
130.817
130.789
126.877
126.793
116.361
116.133
97.706
77.455
77.137
76.819
35.240
13.098
N
N
O
F
1i
150
100
50
ppm (t1)

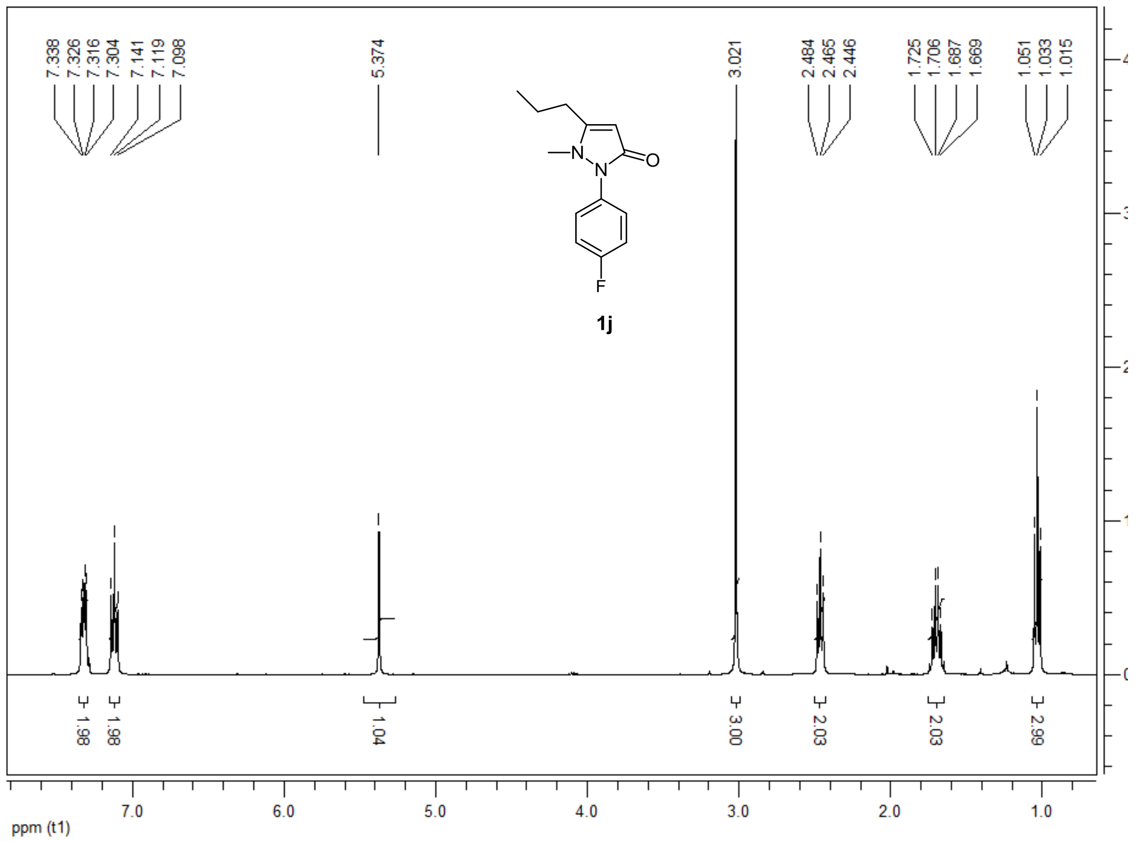
7.338
7.326
7.316
7.304
7.141
7.119
7.098
5.374
3.021
2.484
2.465
2.446
1.725
1.706
1.687
1.669
1.051
1.033
1.015
N
N
O
F
1j
1.98
1.98
1.04
3.00
2.03
2.03
2.99
7.0
6.0
5.0
4.0
3.0
2.0
1.0
ppm (t1)

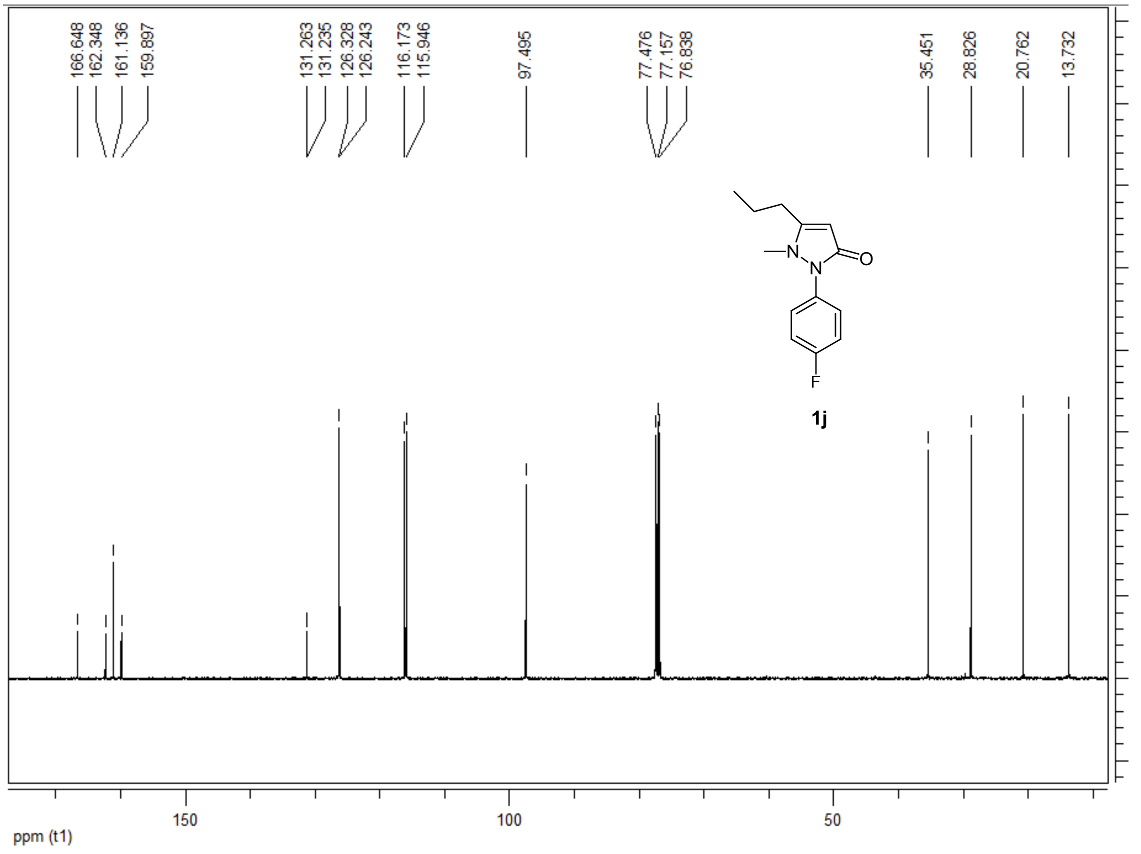
166.648
162.348
161.136
159.897
131.263
131.235
126.328
126.243
116.173
115.946
97.495
77.476
77.157
76.838
35.451
28.826
20.762
13.732
N
N
O
F
1j
150
100
50
ppm (t1)

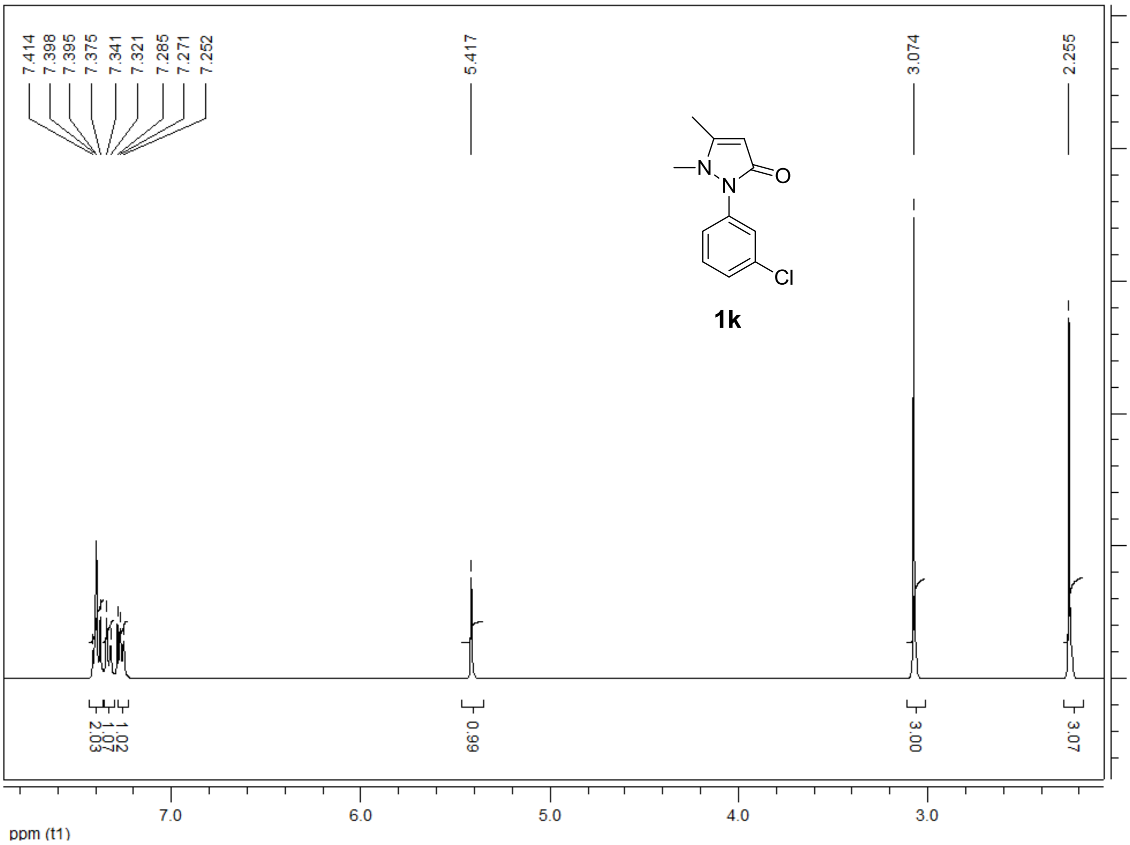
7.414
7.398
7.395
7.375
7.341
7.321
7.285
7.271
7.252
5.417
3.074
2.255
N
N
O
Cl
1k
2.03
1.07
1.02
0.99
3.00
3.07
7.0
6.0
5.0
4.0
3.0
ppm (t1)

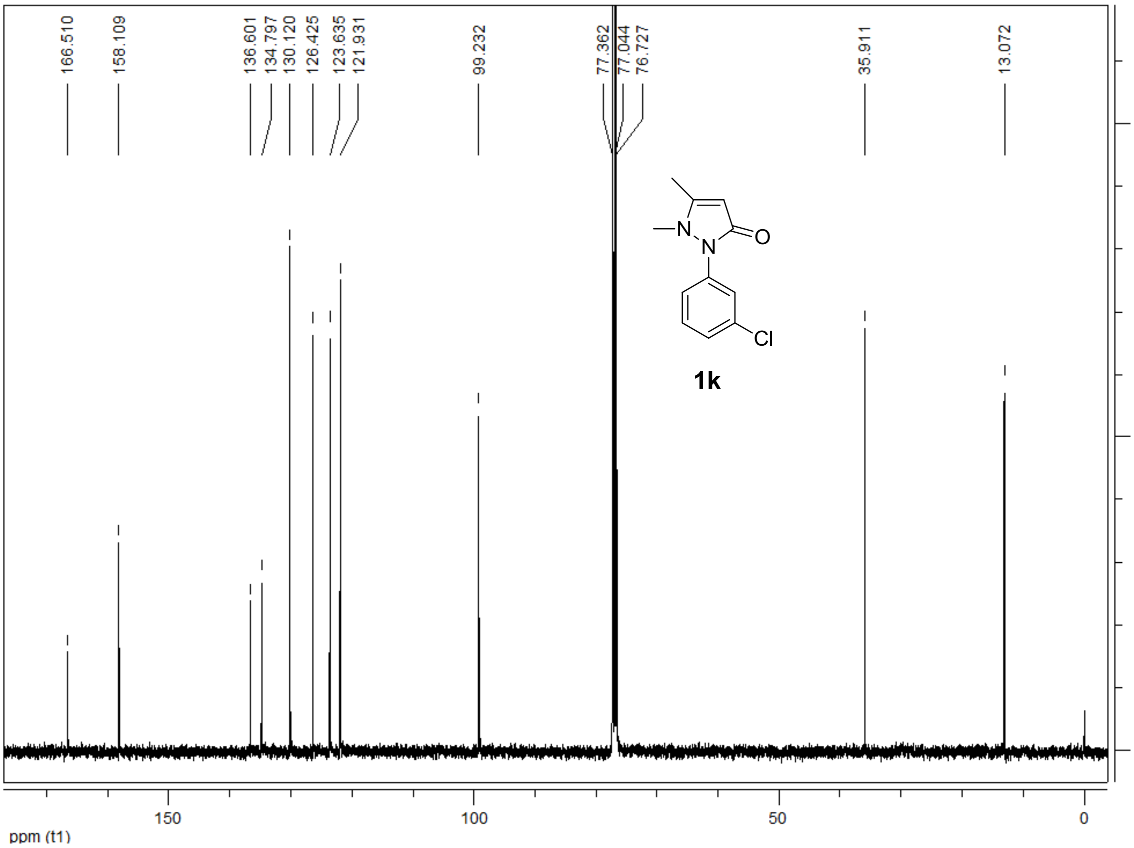
166.510
158.109
136.601
134.797
130.120
126.425
123.635
121.931
99.232
77.362
77.044
76.727
35.911
13.072
N
N
O
Cl
1k
150
100
50
0
ppm (t1)

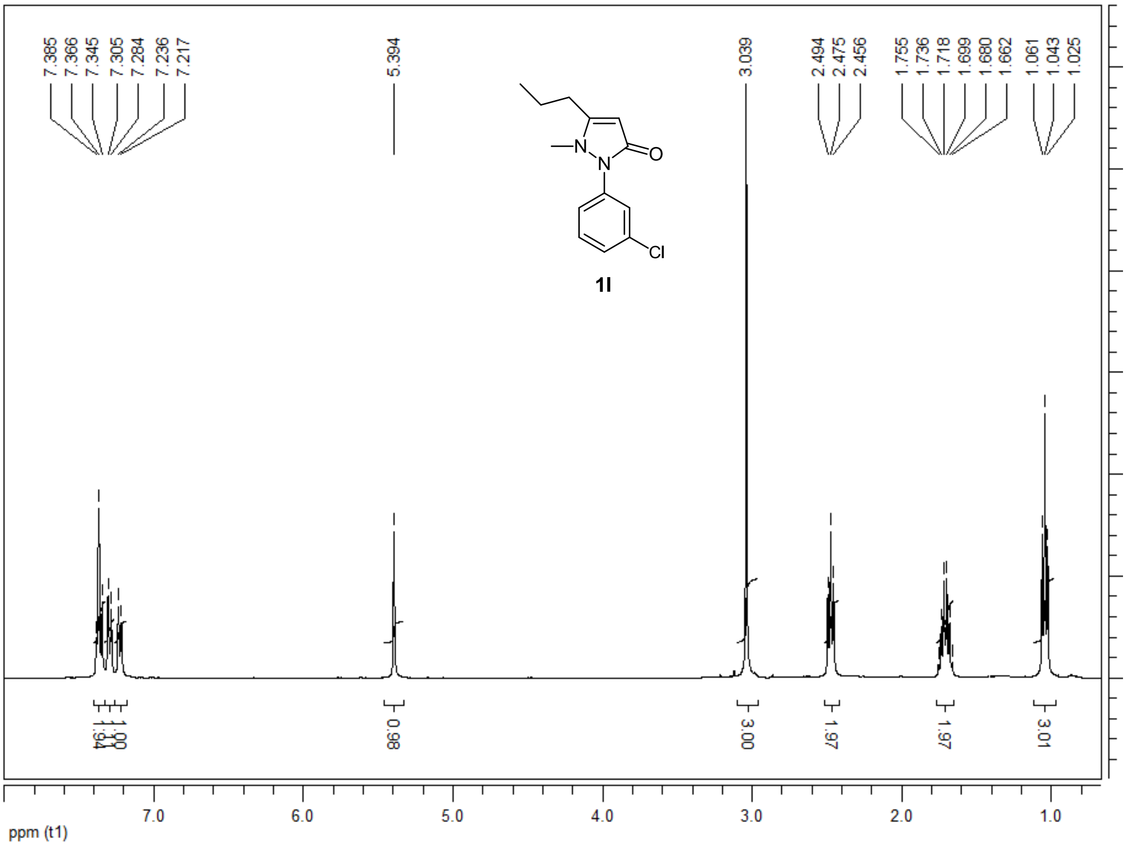
7.385
7.366
7.345
7.305
7.284
7.236
7.217
5.394
3.039
2.494
2.475
2.456
1.755
1.736
1.718
1.699
1.680
1.662
1.061
1.043
1.025
N
N
O
Cl
1l
1.94
1.11
1.00
0.98
3.00
1.97
1.97
3.01
7.0
6.0
5.0
4.0
3.0
2.0
1.0
ppm (t1)

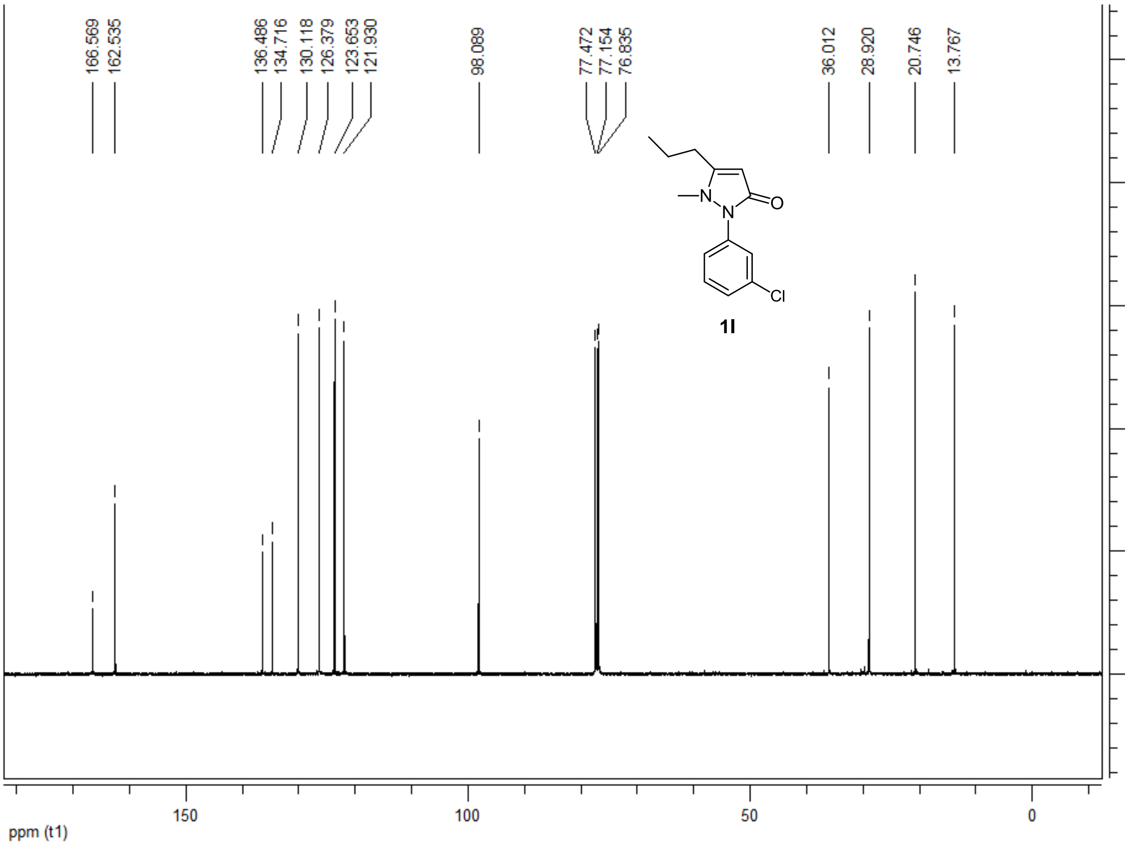
166.569
162.535
136.486
134.716
130.118
126.379
123.653
121.930
98.089
77.472
77.154
76.835
36.012
28.920
20.746
13.767
N
N
O
Cl
1l
150
100
50
0
ppm (t1)

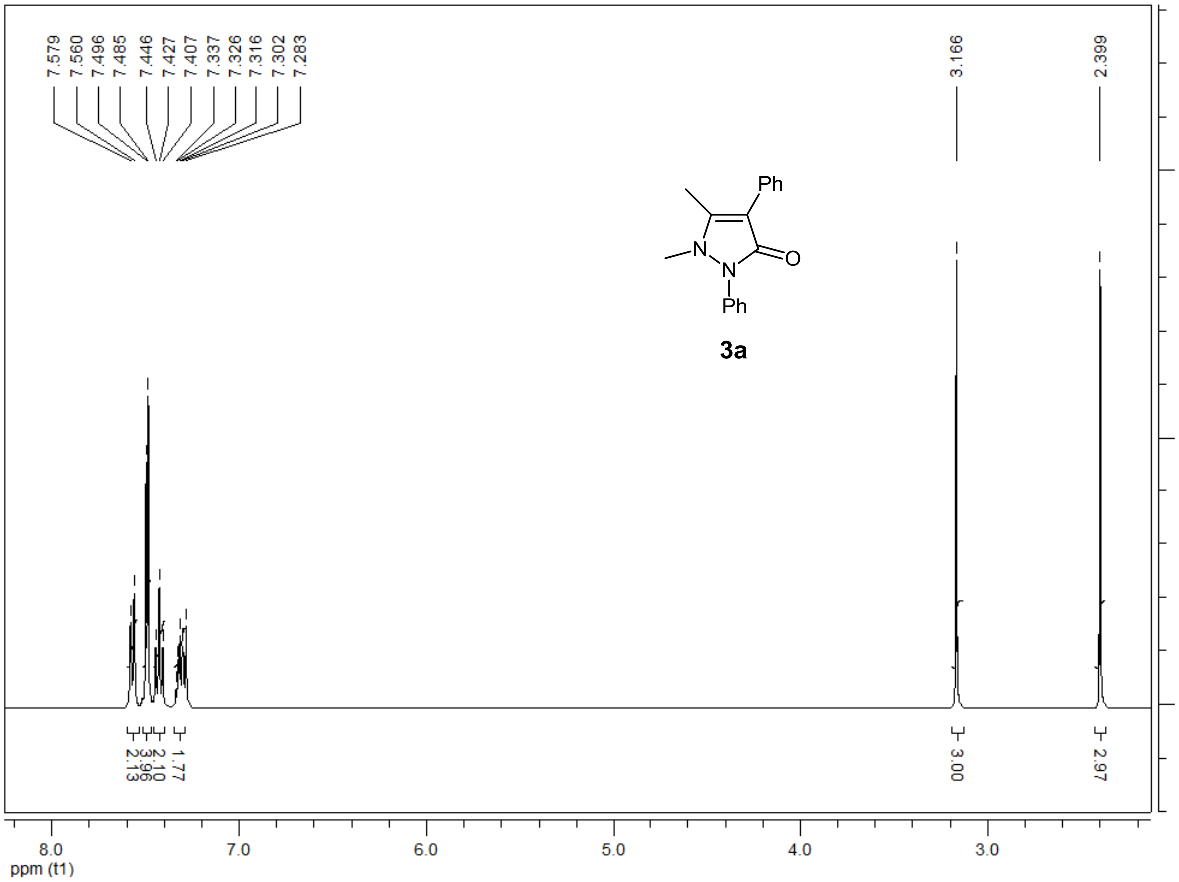
7.579
7.560
7.496
7.485
7.446
7.427
7.407
7.337
7.326
7.316
7.302
7.283
3.166
2.399
Ph
N
N
O
Ph
3a
2.13
3.96
2.10
1.77
3.00
2.97
8.0
7.0
6.0
5.0
4.0
3.0
ppm (t1)

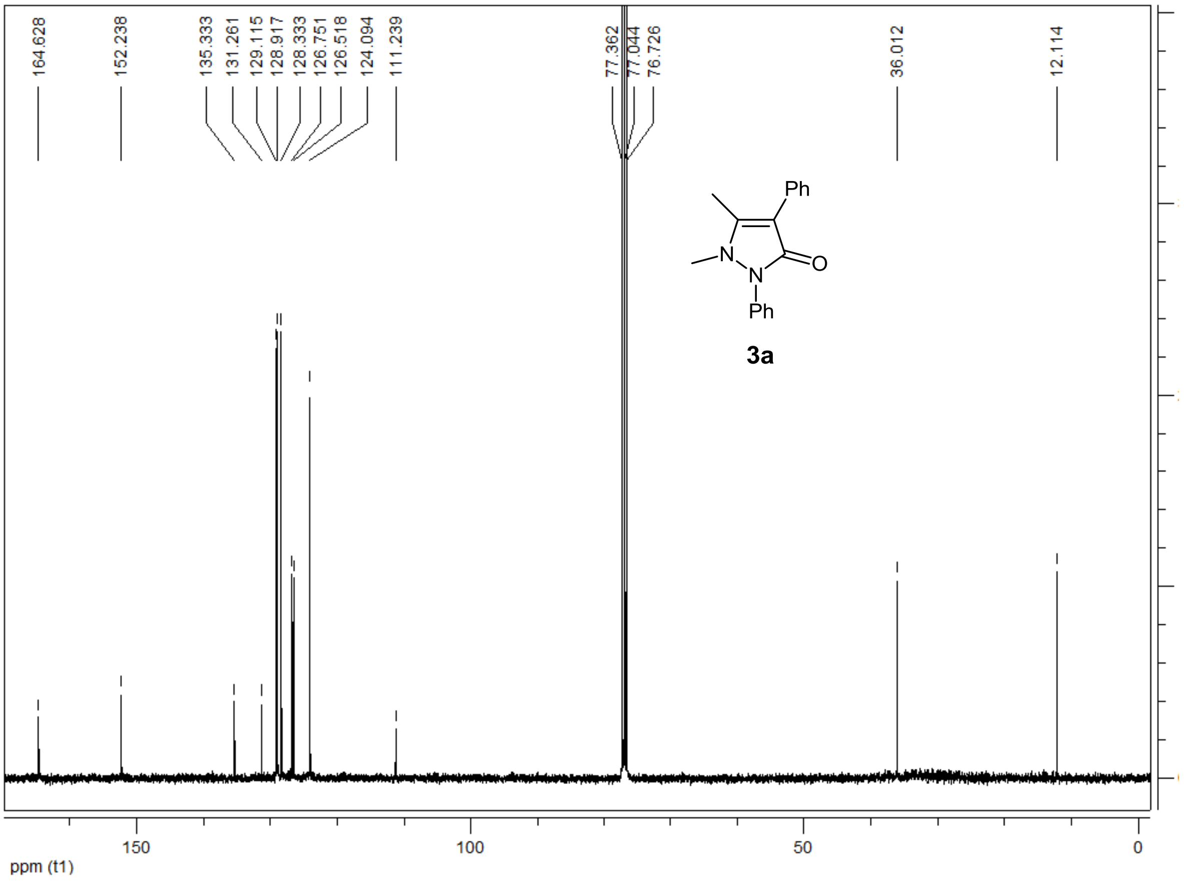
164.628
152.238
135.333
131.261
129.115
128.917
128.333
126.751
126.518
124.094
111.239
77.362
77.044
76.726
36.012
12.114
Ph
N
N
O
Ph
3a
150
100
50
0
ppm (t1)

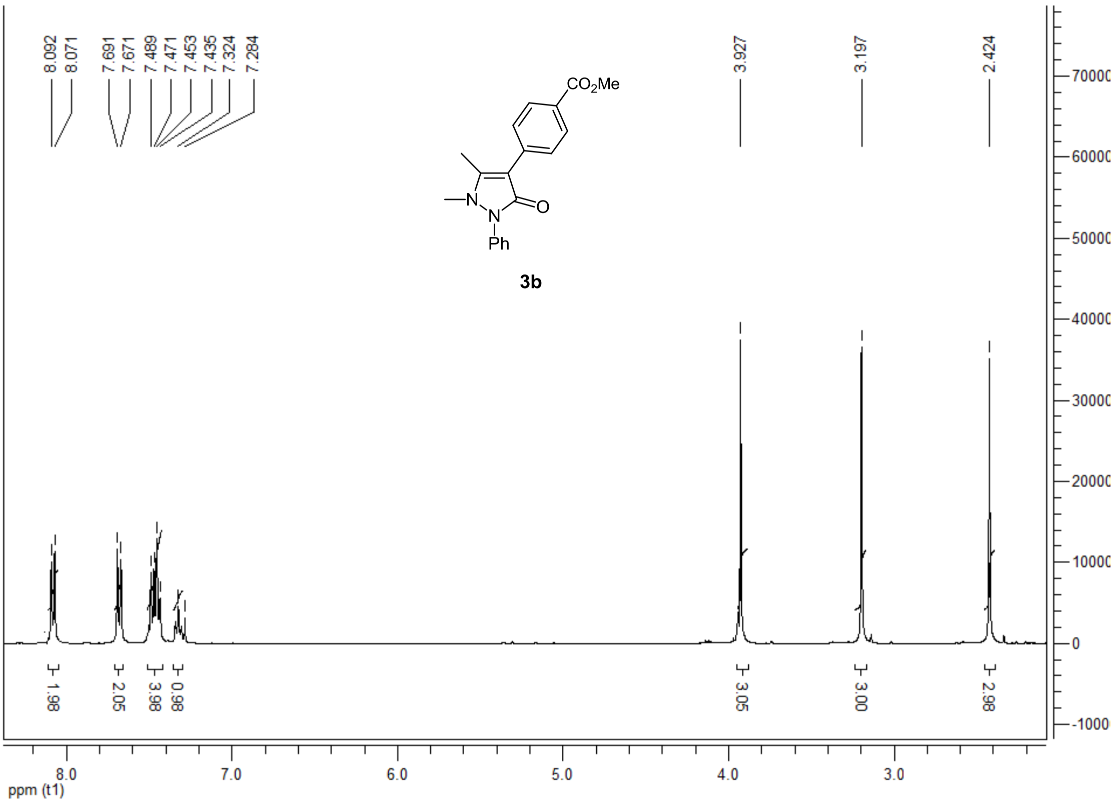
8.092
8.071
7.691
7.671
7.489
7.471
7.453
7.435
7.324
7.284
3.927
3.197
2.424
CO2Me
N
N
O
Ph
3b
1.98
2.05
3.98
0.98
3.05
3.00
2.98
ppm (t1)

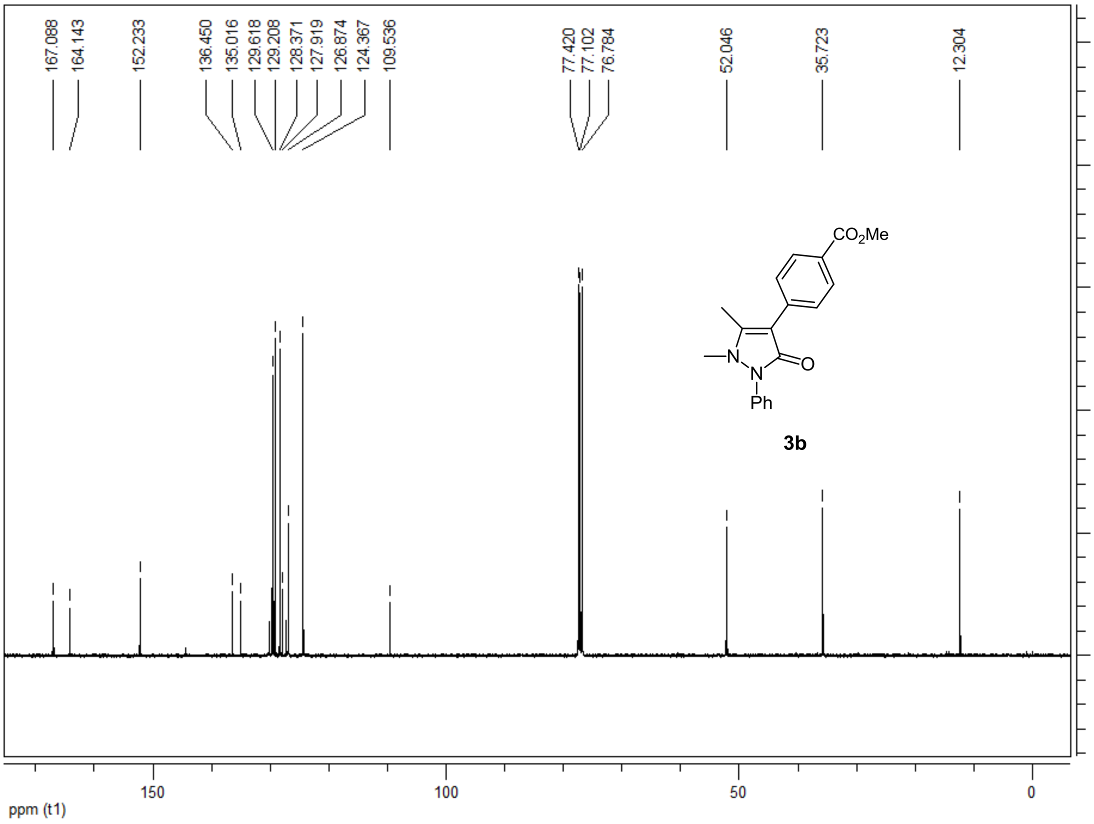
167.088
164.143
152.233
136.450
135.016
129.618
129.208
128.371
127.919
126.874
124.367
109.536
77.420
77.102
76.784
52.046
35.723
12.304
CO2Me
N
N
O
Ph
3b
ppm (t1)

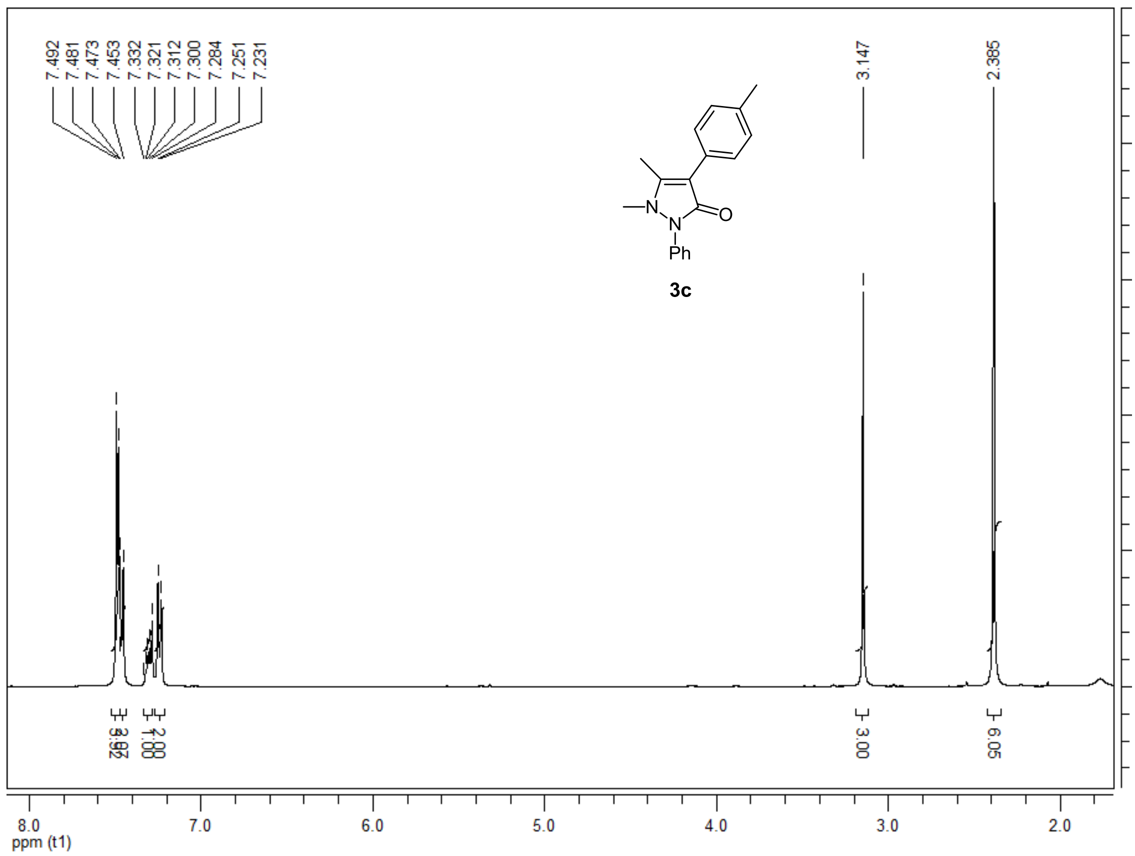
7.492
7.481
7.473
7.453
7.332
7.321
7.312
7.300
7.284
7.251
7.231
3.147
2.385
N
N
O
Ph
3c
3.00
6.05
2.00
1.00
8.0
7.0
6.0
5.0
4.0
3.0
2.0
ppm (t1)

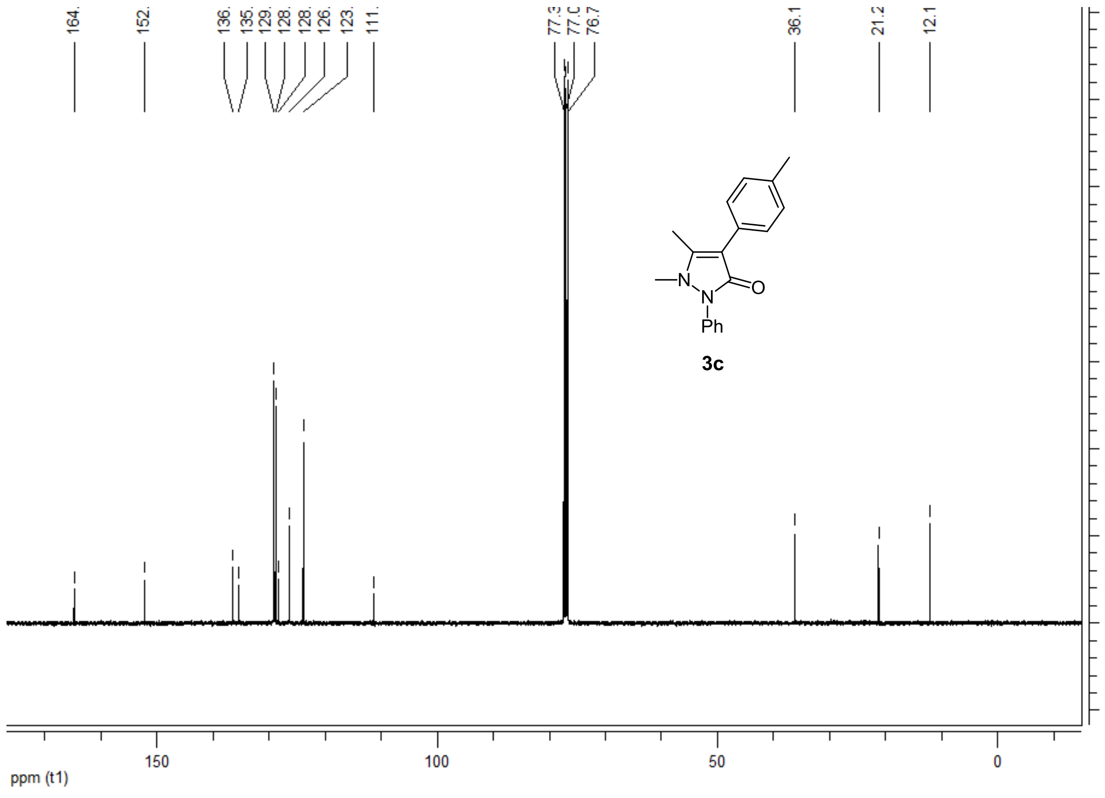
164.
152.
136.
135.
129.
128.
128.
126.
123.
111.
76.7
36.1
12.1
N
N
O
Ph
3c
150
100
50
0
ppm (t1)

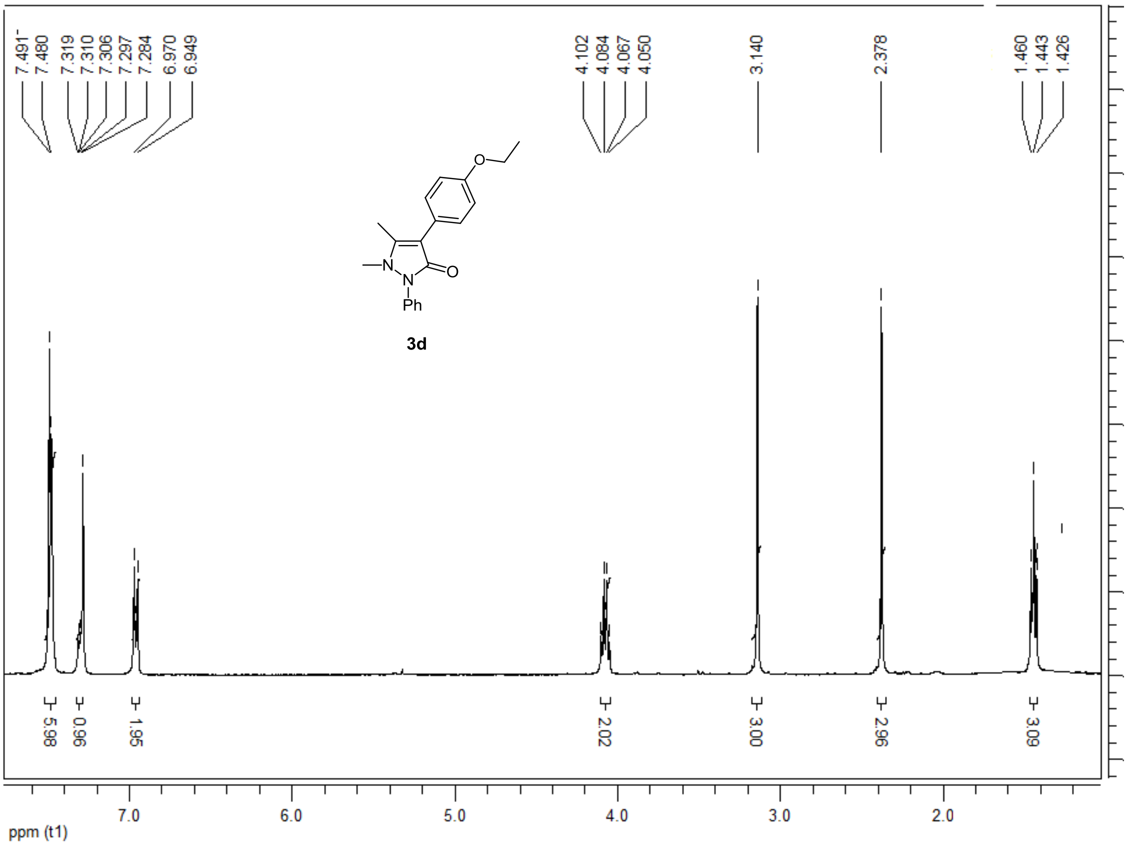
7.491
7.480
7.319
7.310
7.306
7.297
7.284
6.970
6.949
4.102
4.084
4.067
4.050
3.140
2.378
1.460
1.443
1.426
Ph
3d
5.98
0.96
1.95
2.02
3.00
2.96
3.09
7.0
6.0
5.0
4.0
3.0
2.0
ppm (t1)

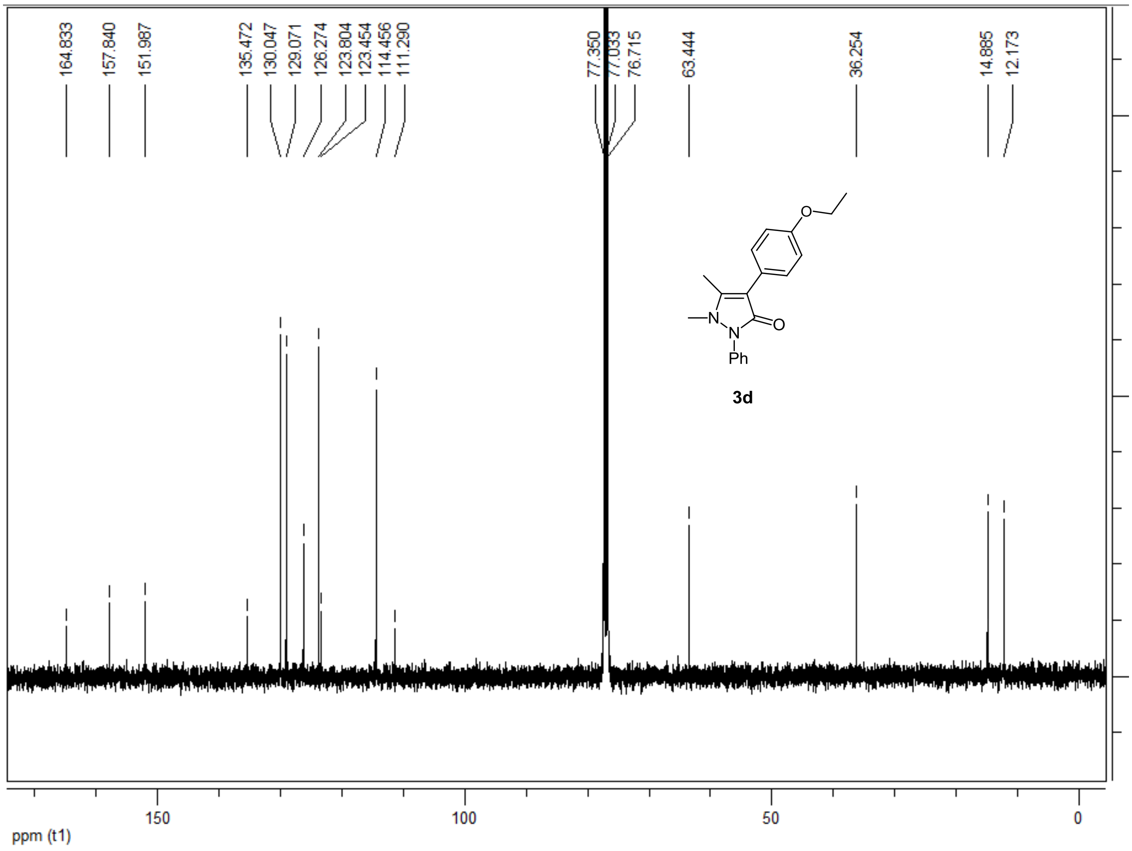
164.833
157.840
151.987
135.472
130.047
129.071
126.274
123.804
123.454
114.456
111.290
77.350
77.033
76.715
63.444
36.254
14.885
12.173
Ph
3d
150
100
50
0
ppm (t1)

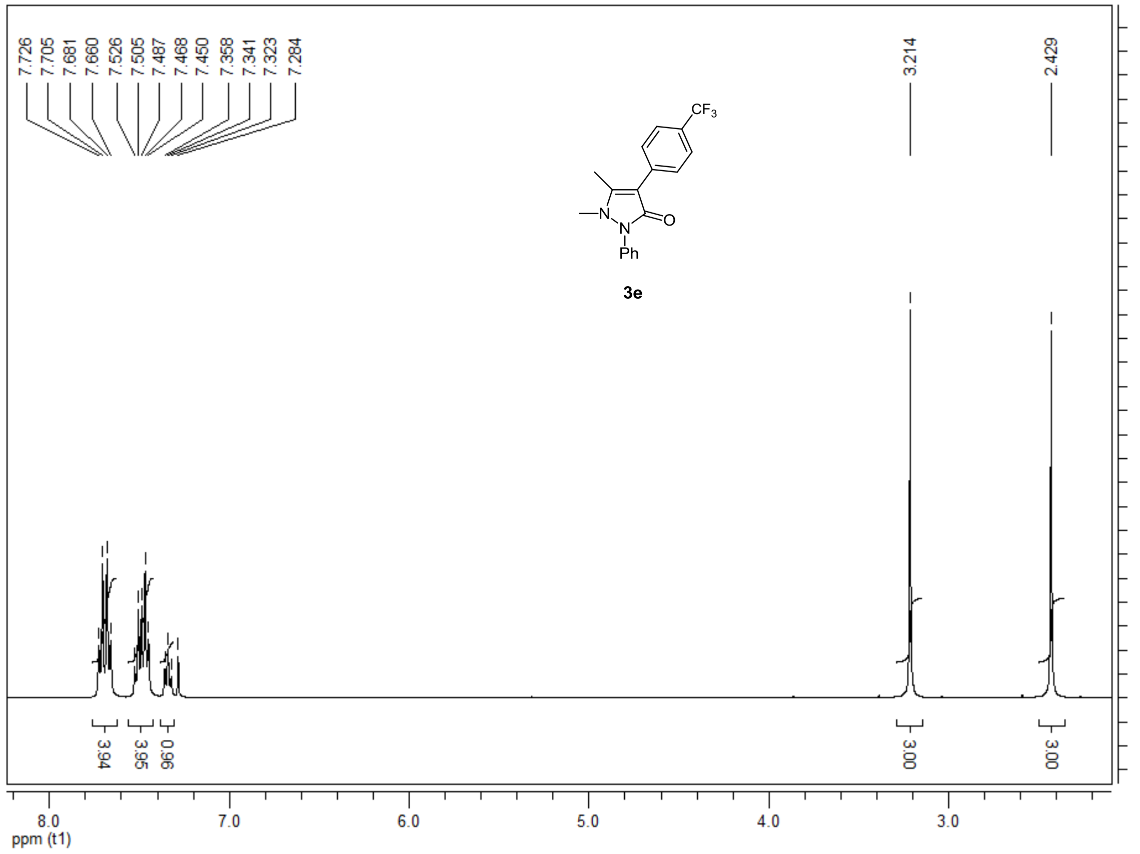
7.726
7.705
7.681
7.660
7.526
7.505
7.487
7.468
7.450
7.358
7.341
7.323
7.284
3.214
2.429
CF3
N
N
O
Ph
3e
3.94
3.95
0.96
3.00
3.00
8.0
7.0
6.0
5.0
4.0
3.0
ppm (t1)

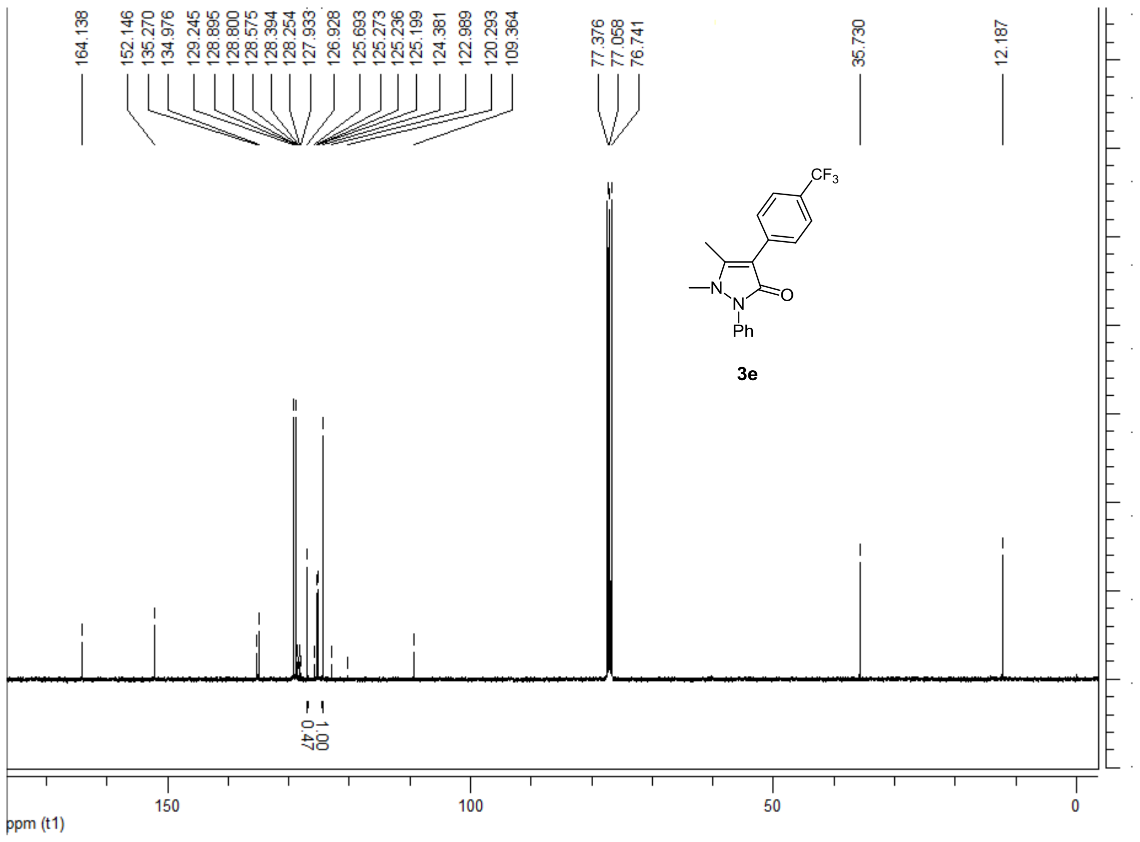
164.138
152.146
135.270
134.976
129.245
128.895
128.800
128.575
128.394
128.254
127.933
126.928
125.693
125.273
125.236
125.199
124.381
122.989
120.293
109.364
77.376
77.058
76.741
35.730
12.187
CF3
N
N
O
Ph
3e
0.47
1.00
150
100
50
0
ppm (t1)

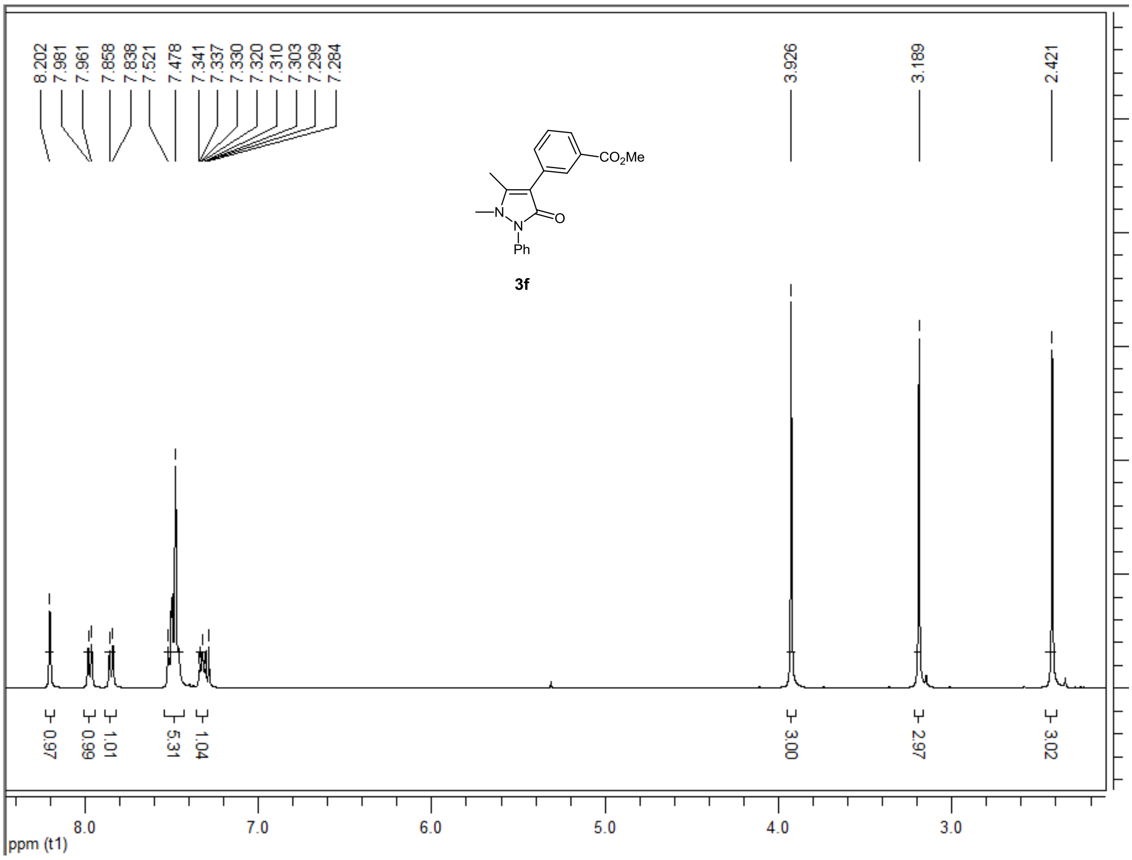
8.202
7.981
7.961
7.858
7.838
7.521
7.478
7.341
7.337
7.330
7.320
7.310
7.303
7.299
7.284
3.926
3.189
2.421
CO2Me
N
N
O
Ph
3f
0.97
0.99
1.01
5.31
1.04
3.00
2.97
3.02
8.0
7.0
6.0
5.0
4.0
3.0
ppm (t1)

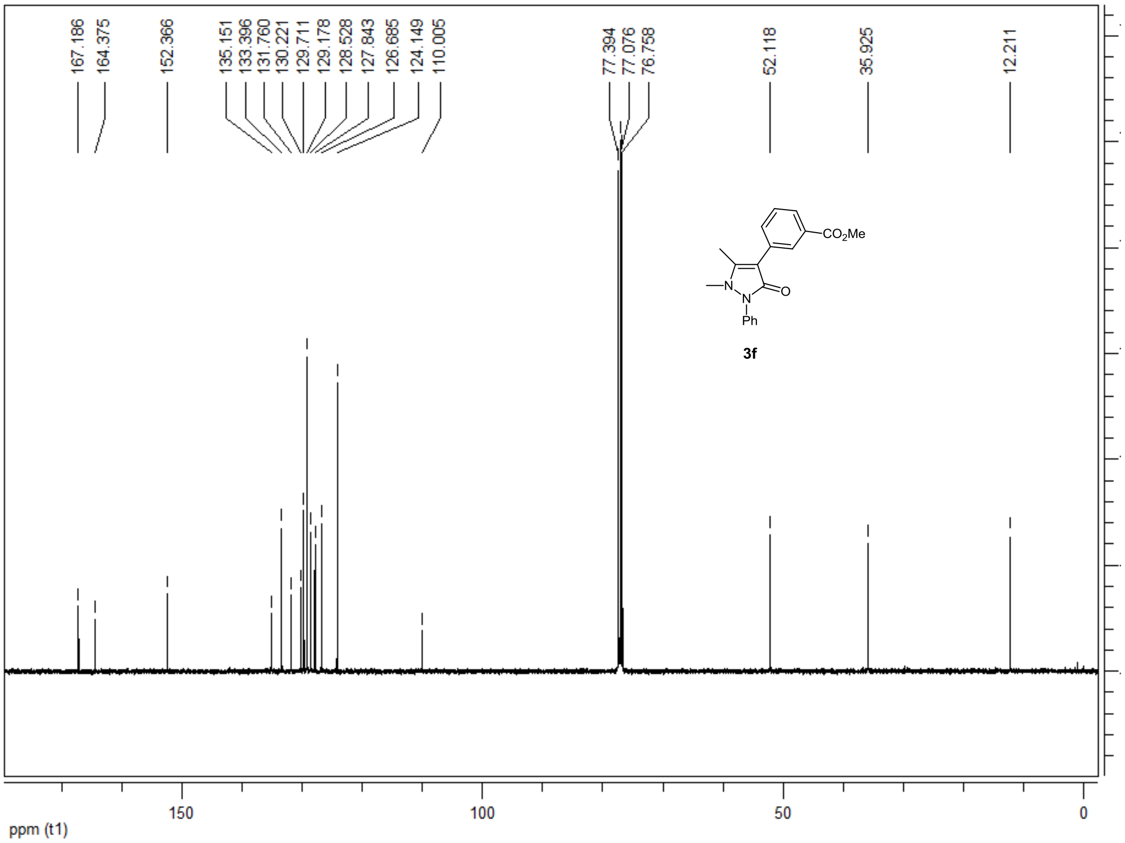
167.186
164.375
152.366
135.151
133.396
131.760
130.221
129.711
129.178
128.528
127.843
126.685
124.149
110.005
77.394
77.076
76.758
52.118
35.925
12.211
CO2Me
N
N
O
Ph
3f
150
100
50
0
ppm (t1)

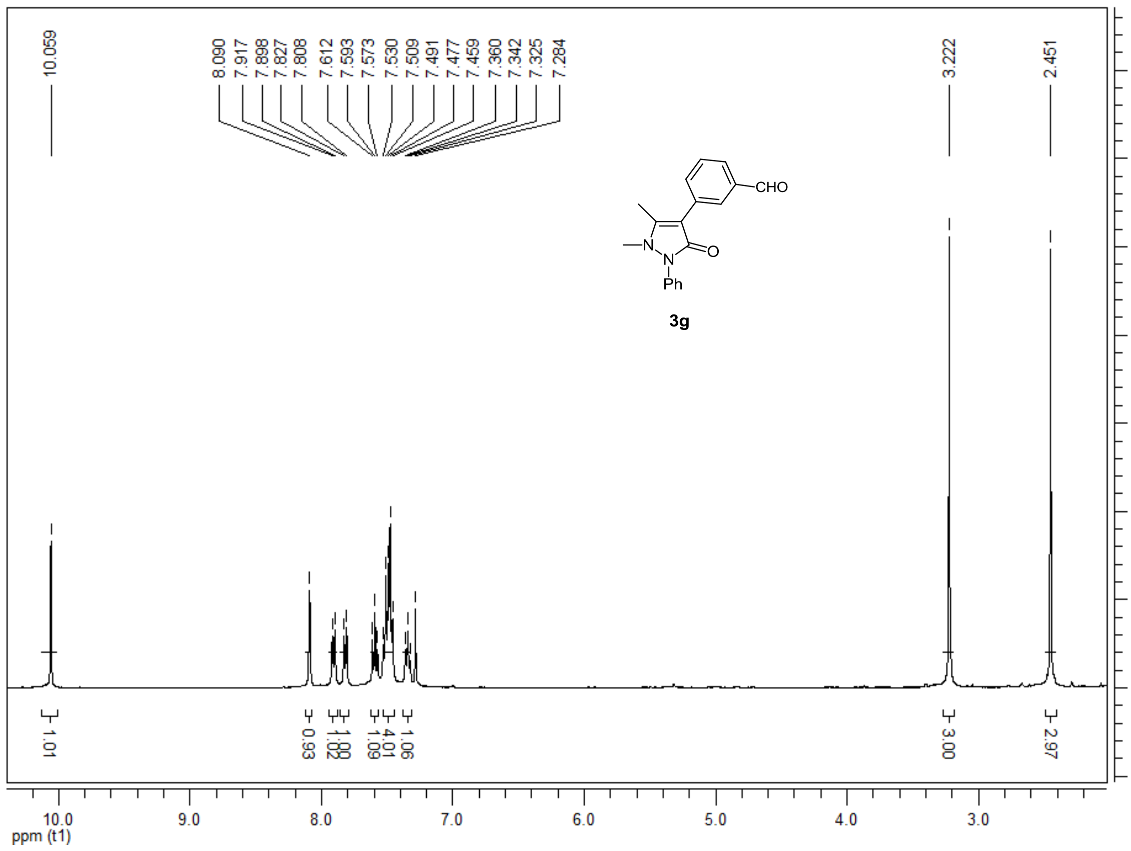
10.059
8.090
7.917
7.898
7.827
7.808
7.612
7.593
7.573
7.530
7.509
7.491
7.477
7.459
7.360
7.342
7.325
7.284
3.222
2.451
CHO
N
N
O
Ph
3g
1.01
0.93
1.02
1.00
1.09
4.01
1.06
3.00
2.97
10.0
9.0
8.0
7.0
6.0
5.0
4.0
3.0
ppm (t1)

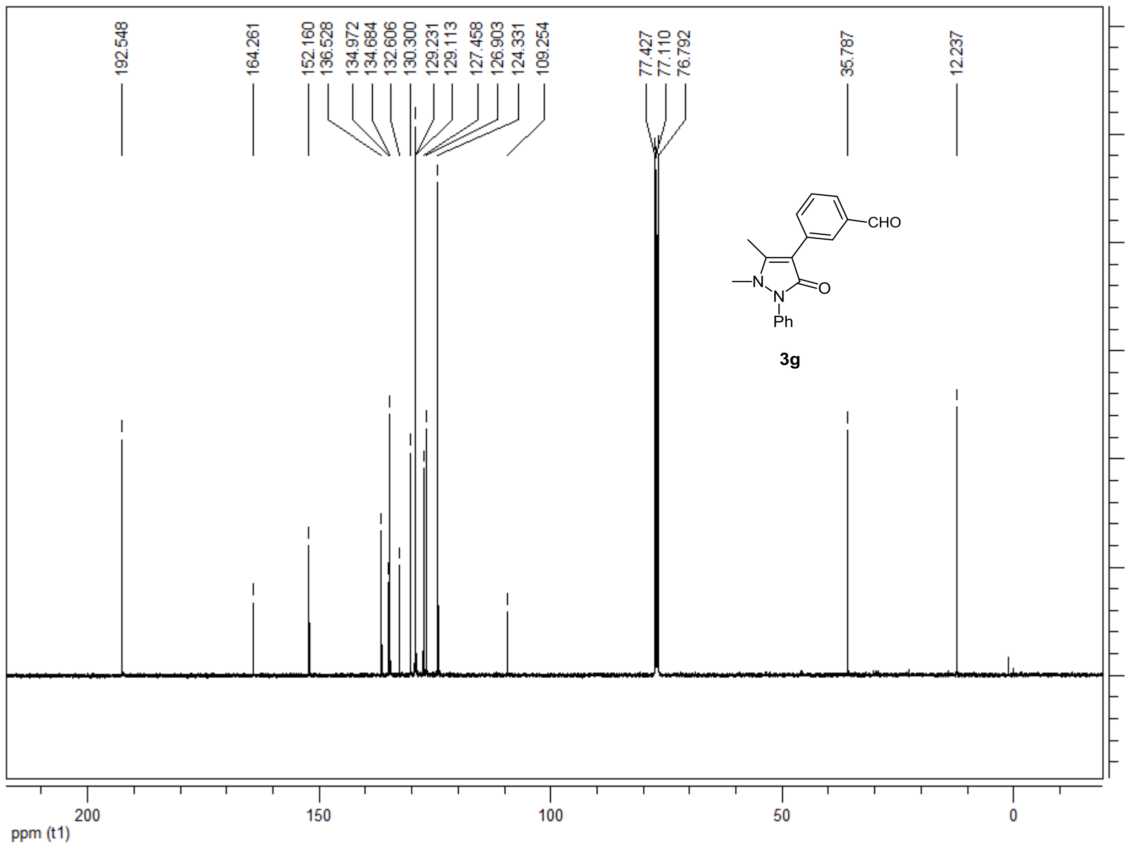
192.548
164.261
152.160
136.528
134.972
134.684
132.606
130.300
129.231
129.113
127.458
126.903
124.331
109.254
77.427
77.110
76.792
35.787
12.237
CHO
N
N
O
Ph
3g
200
150
100
50
0
ppm (t1)

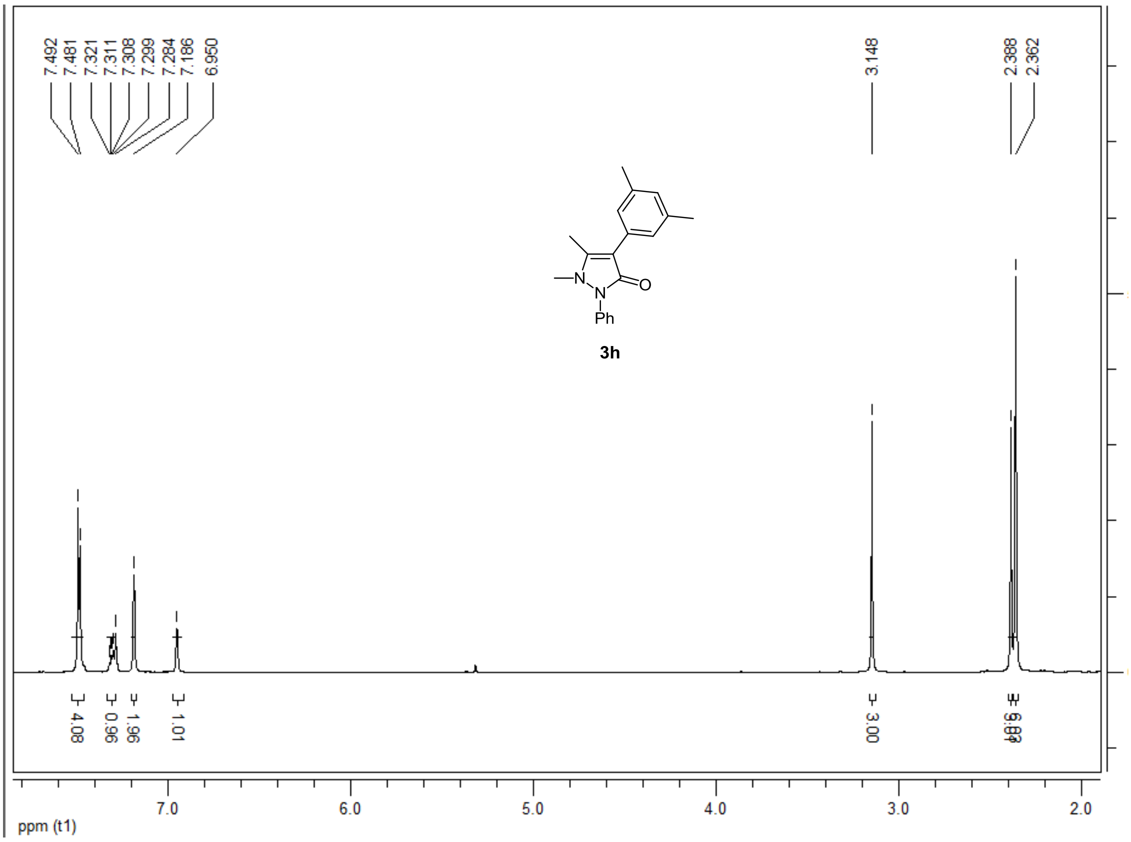
7.492
7.481
7.321
7.311
7.308
7.299
7.284
7.186
6.950
3.148
2.388
2.362
Ph
3h
4.08
0.96
1.96
1.01
3.00
6.08
7.0
6.0
5.0
4.0
3.0
2.0
ppm (t1)

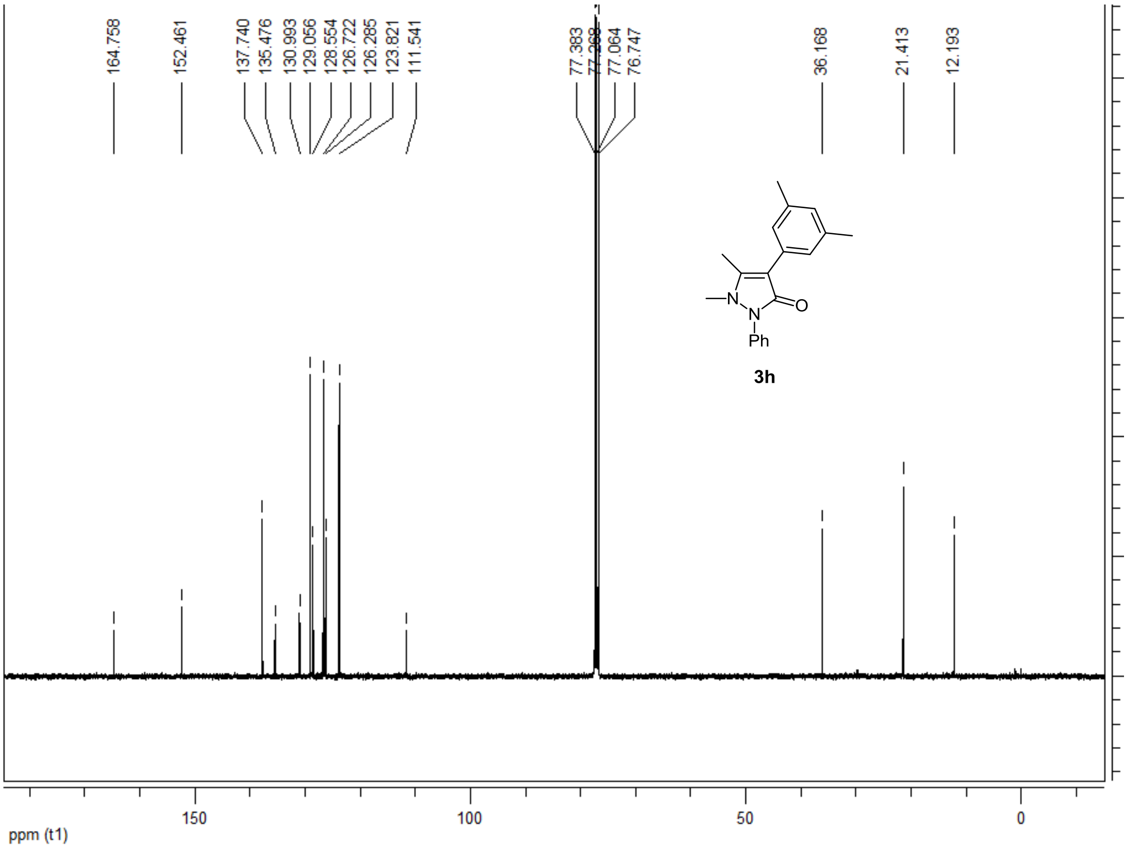
164.758
152.461
137.740
135.476
130.993
129.056
128.554
126.722
126.285
123.821
111.541
77.383
77.268
77.064
76.747
36.168
21.413
12.193
Ph
3h
150
100
50
0
ppm (t1)

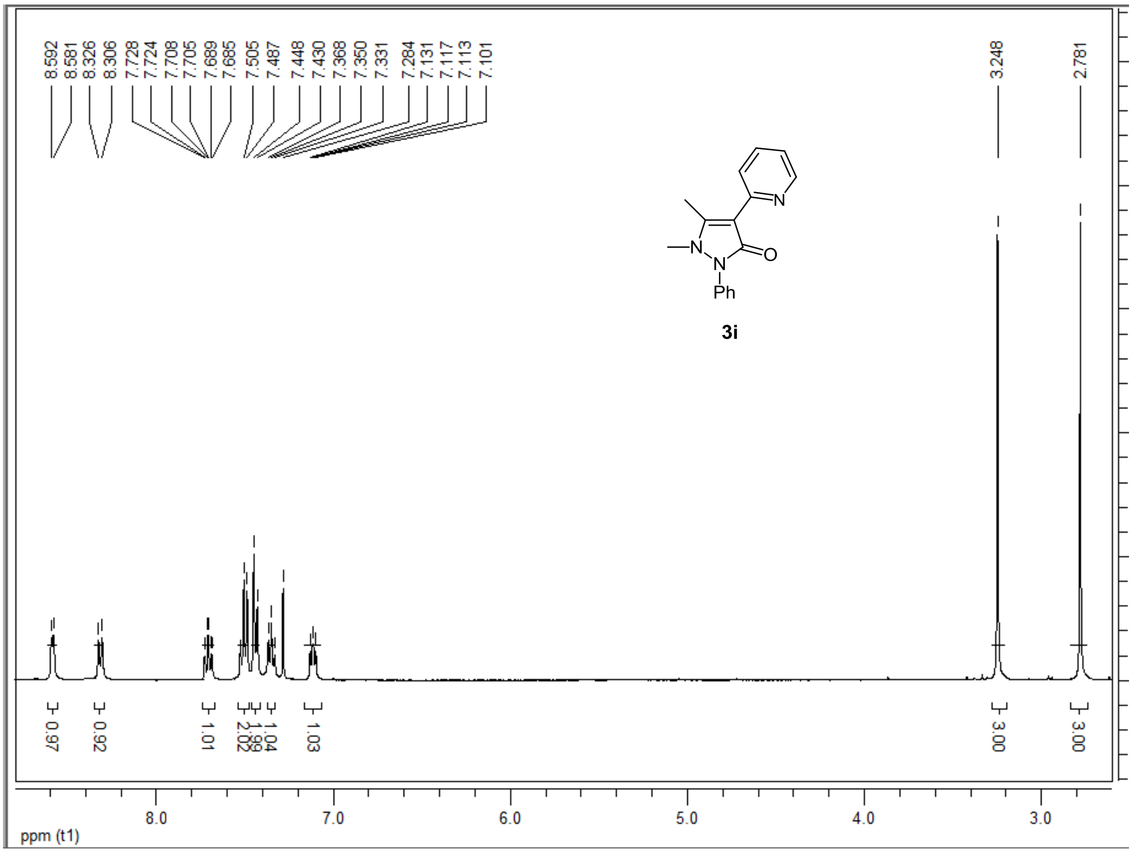

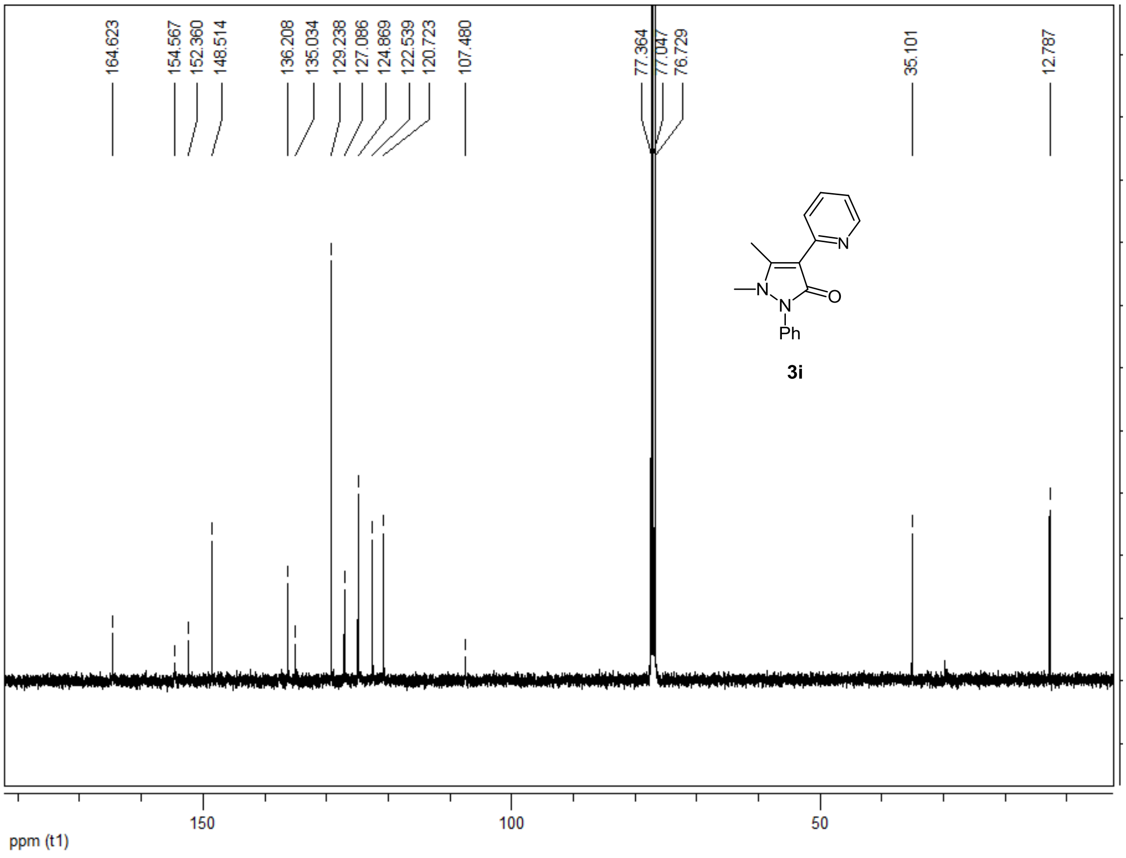

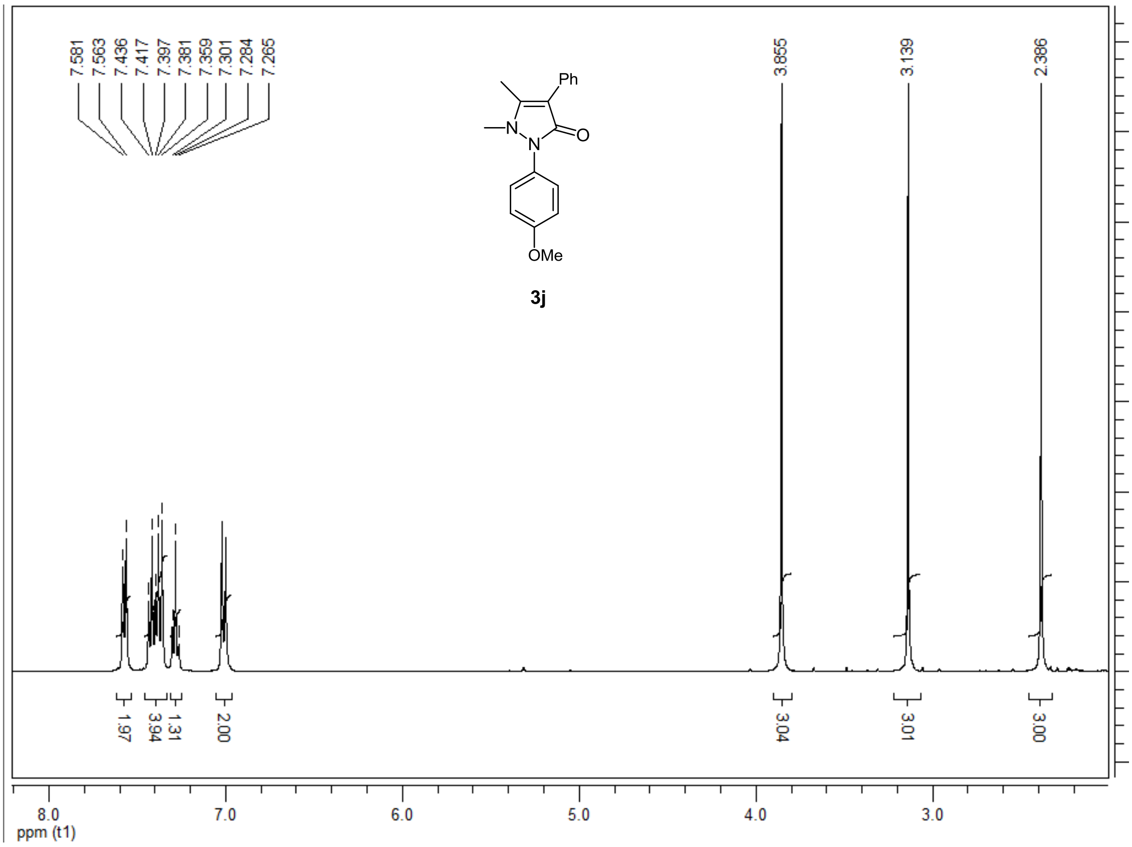
7.581
7.563
7.436
7.417
7.397
7.381
7.359
7.301
7.284
7.265
3.855
3.139
2.386
Ph
N
N
O
OMe
3j
1.97
3.94
1.31
2.00
3.04
3.01
3.00
8.0
7.0
6.0
5.0
4.0
3.0
ppm (t1)

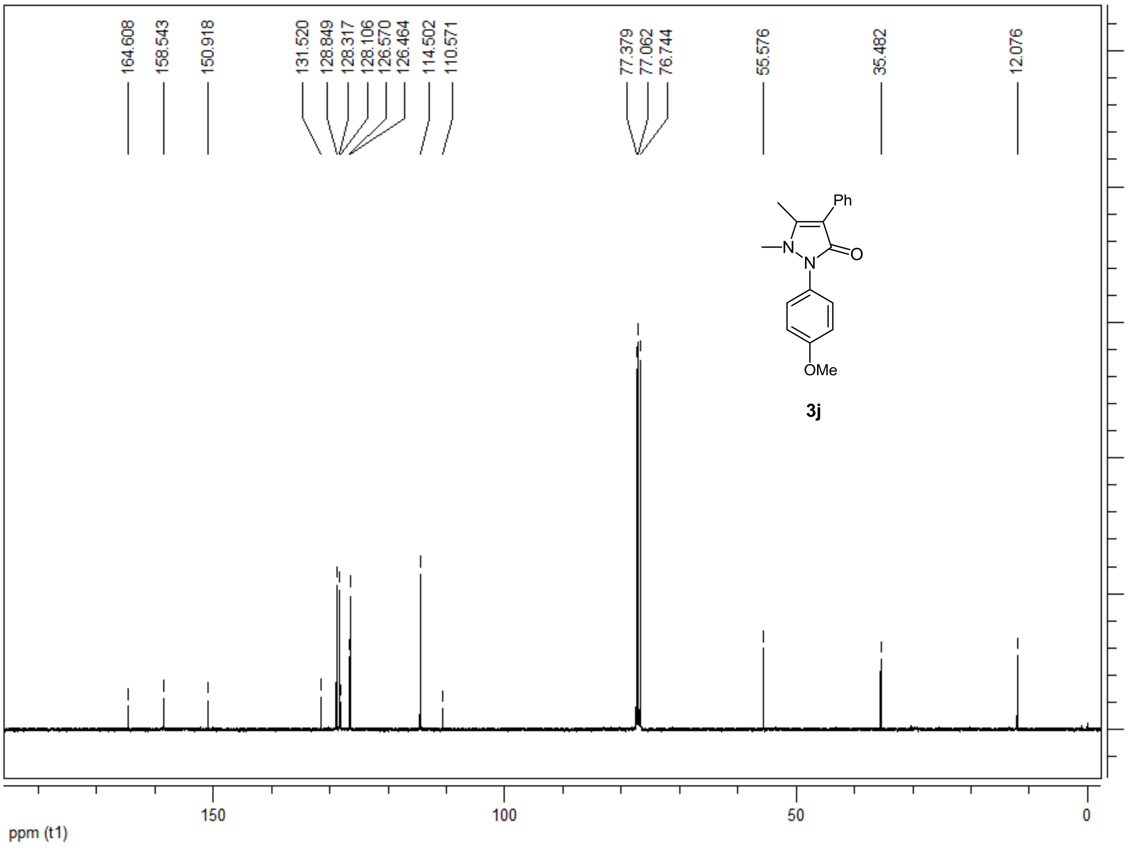
164.608
158.543
150.918
131.520
128.849
128.317
128.106
126.570
126.464
114.502
110.571
77.379
77.062
76.744
55.576
35.482
12.076
Ph
N
N
O
OMe
3j
150
100
50
0
ppm (t1)

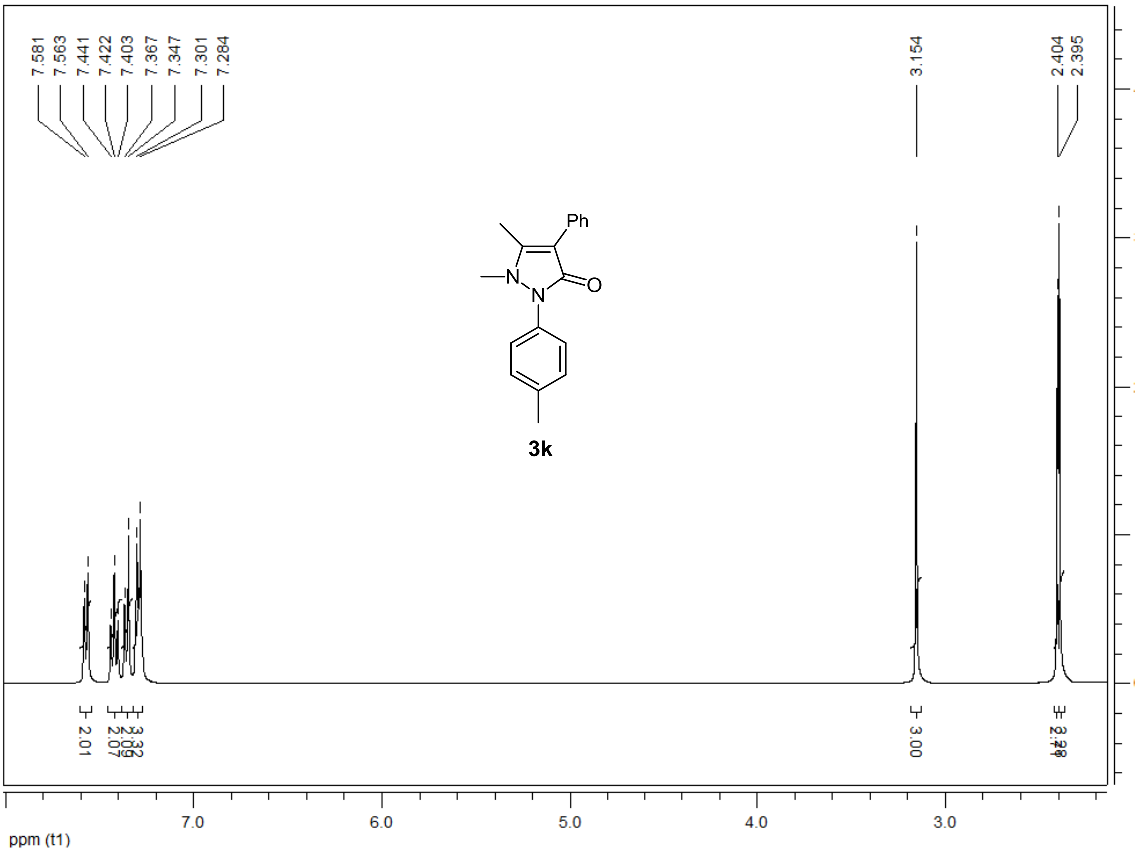

7.581
7.563
7.441
7.422
7.403
7.367
7.347
7.301
7.284
3.154
2.404
2.395
Ph
N
N
O
3k
2.01
2.07
2.09
3.32
3.00
ppm (t1)
7.0
6.0
5.0
4.0
3.0

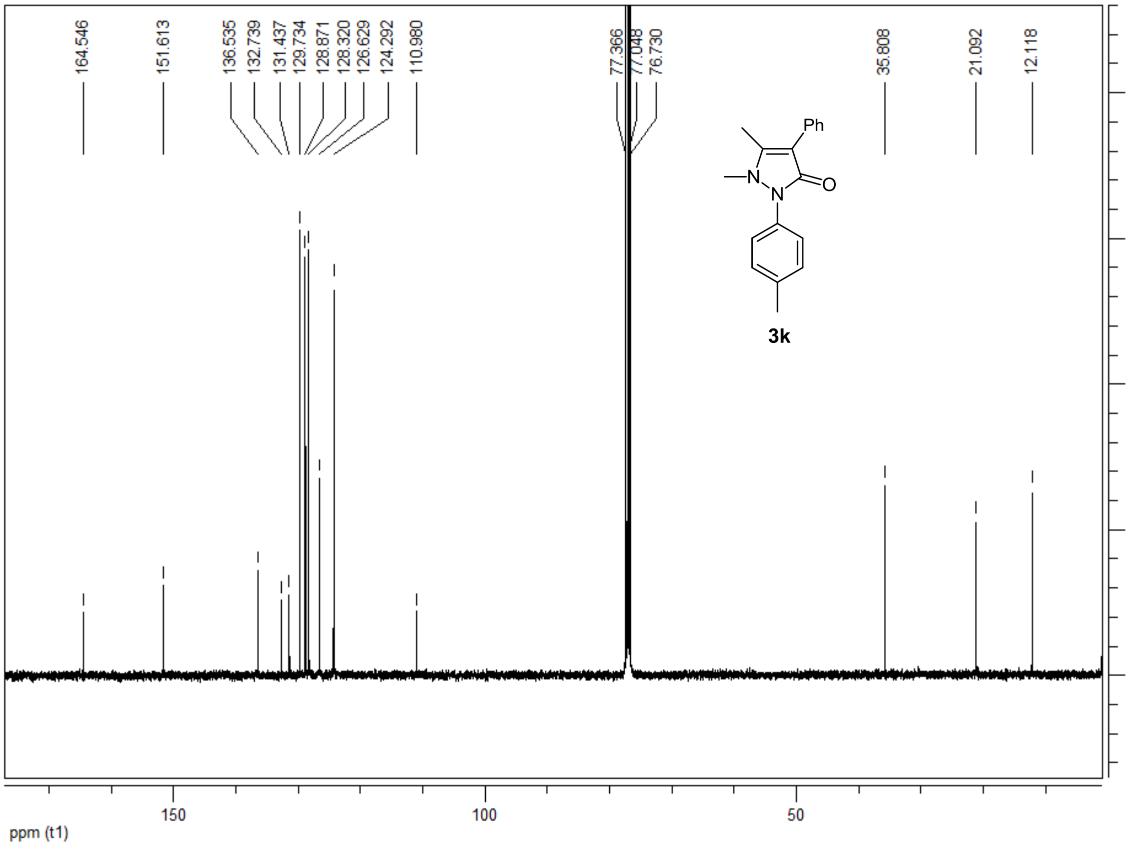

164.546
151.613
136.535
132.739
131.437
129.734
128.871
128.320
126.629
124.292
110.980
77.366
77.048
76.730
35.808
21.092
12.118
Ph
N
N
O
3k
ppm (t1)
150
100
50

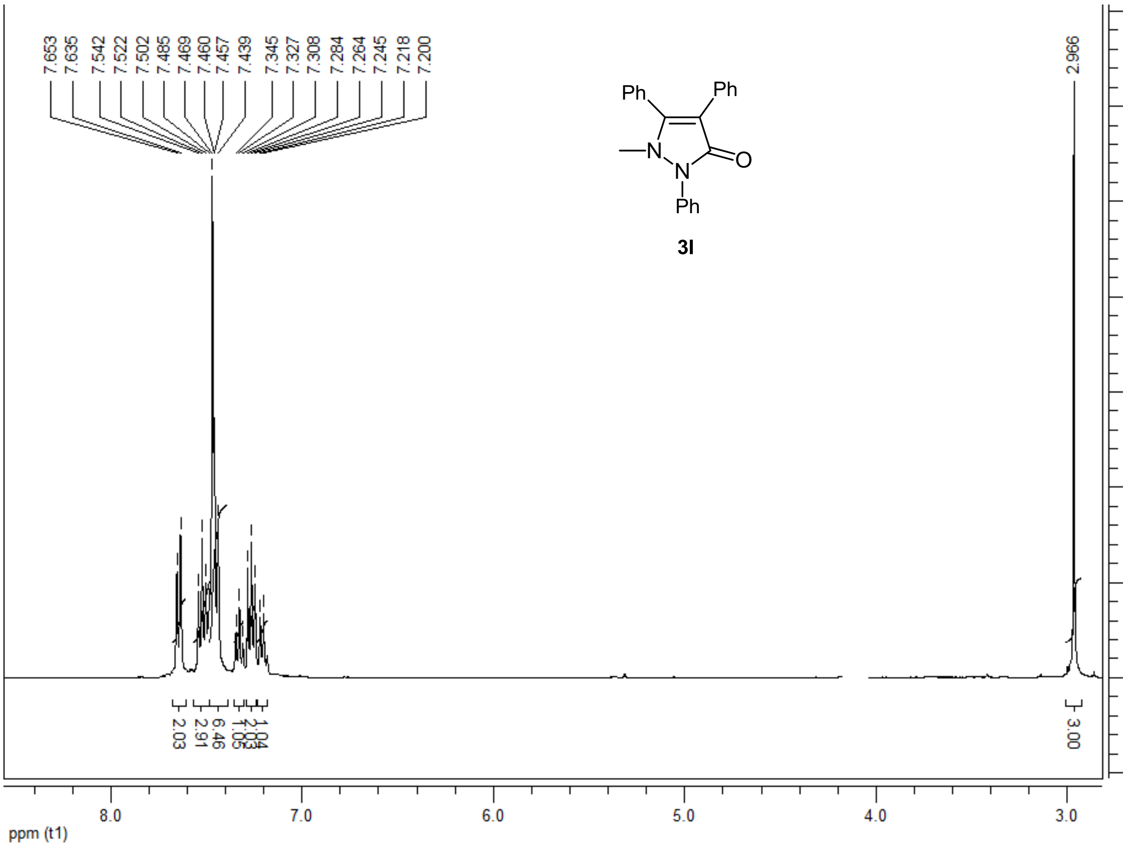
7.653
7.635
7.542
7.522
7.502
7.485
7.469
7.460
7.457
7.439
7.345
7.327
7.308
7.284
7.264
7.245
7.218
7.200
2.966
Ph
Ph
N
N
O
Ph
3l
2.03
2.91
6.46
1.05
2.03
1.04
3.00
8.0
7.0
6.0
5.0
4.0
3.0
ppm (t1)

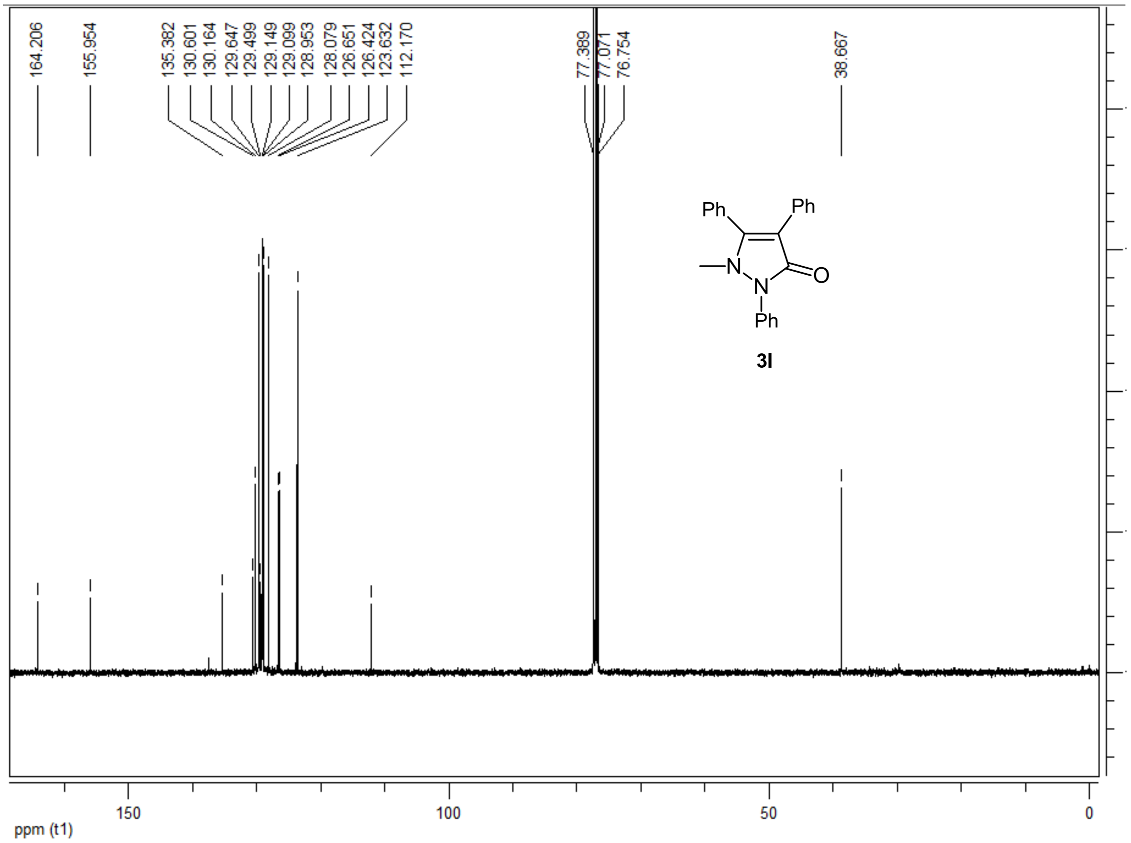
164.206
155.954
135.382
130.601
130.164
129.647
129.499
129.149
129.099
128.953
128.079
126.651
126.424
123.632
112.170
77.389
77.071
76.754
38.667
Ph
Ph
N
N
O
Ph
3l
150
100
50
0
ppm (t1)

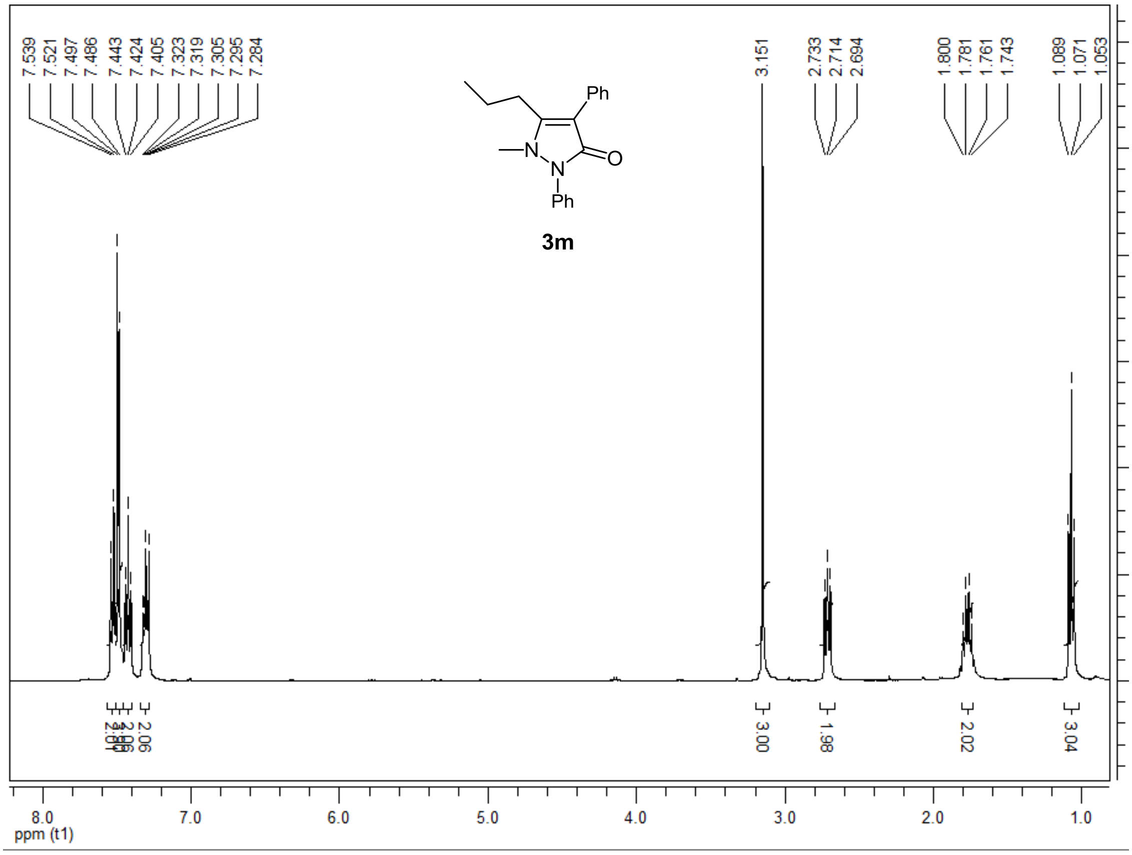
7.539
7.521
7.497
7.486
7.443
7.424
7.405
7.323
7.319
7.305
7.295
7.284
3.151
2.733
2.714
2.694
1.800
1.781
1.761
1.743
1.089
1.071
1.053
Ph
N
N
O
Ph
3m
2.06
3.00
1.98
2.02
3.04
8.0
7.0
6.0
5.0
4.0
3.0
2.0
1.0
ppm (t1)

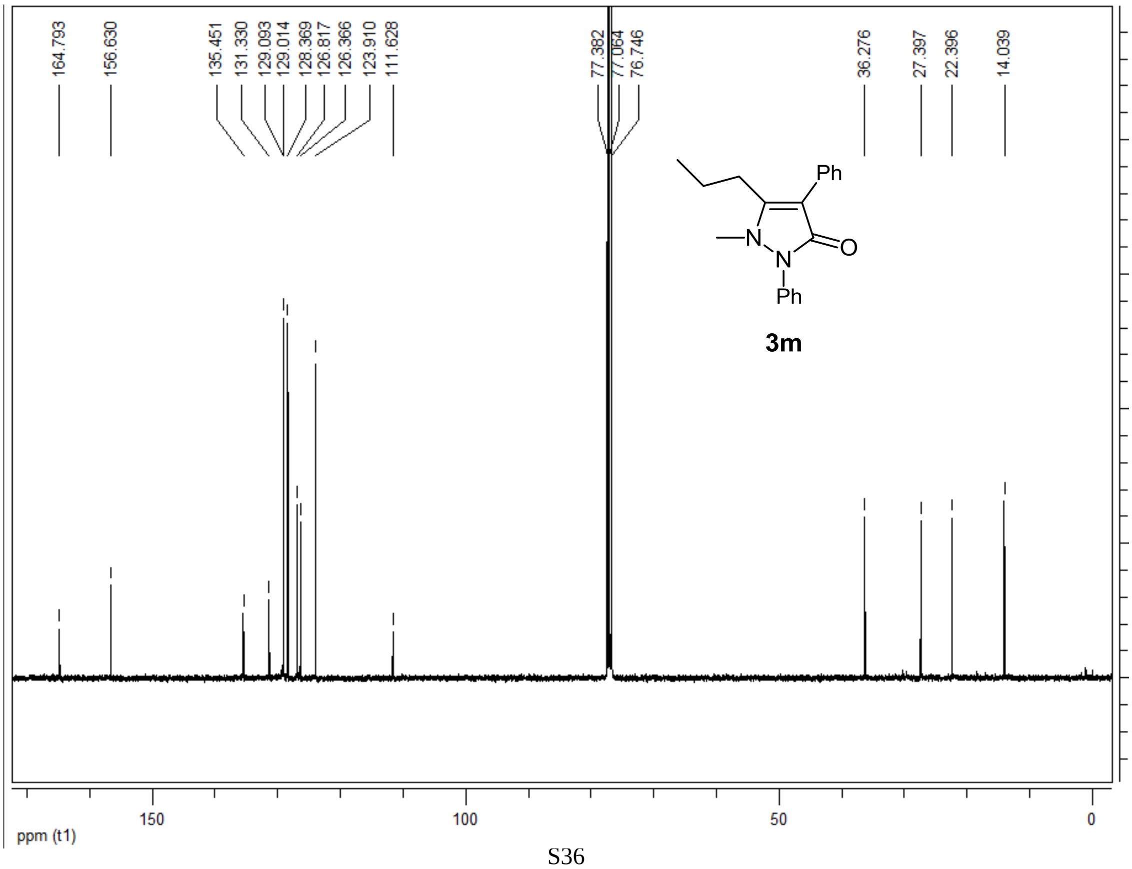
164.793
156.630
135.451
131.330
129.093
129.014
128.369
126.817
126.366
123.910
111.628
77.382
77.064
76.746
36.276
27.397
22.396
14.039
Ph
N
N
O
Ph
3m
150
100
50
0
ppm (t1)

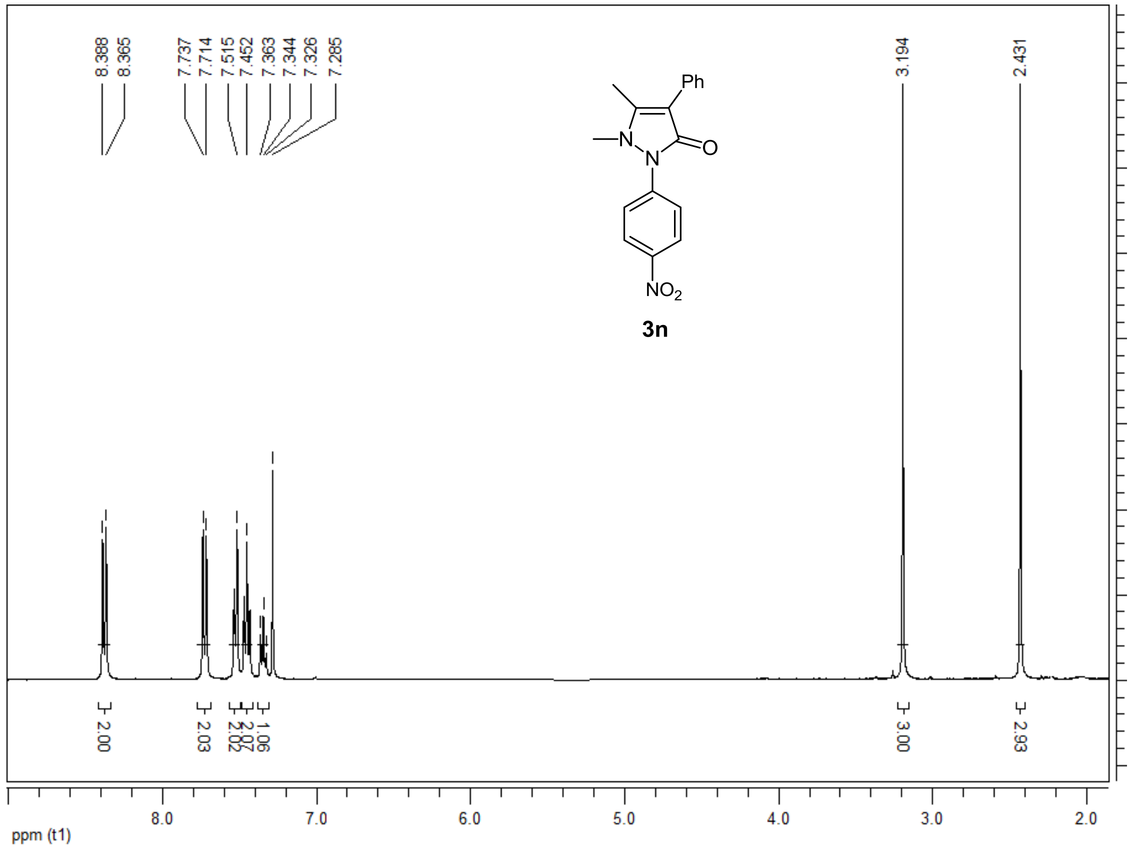

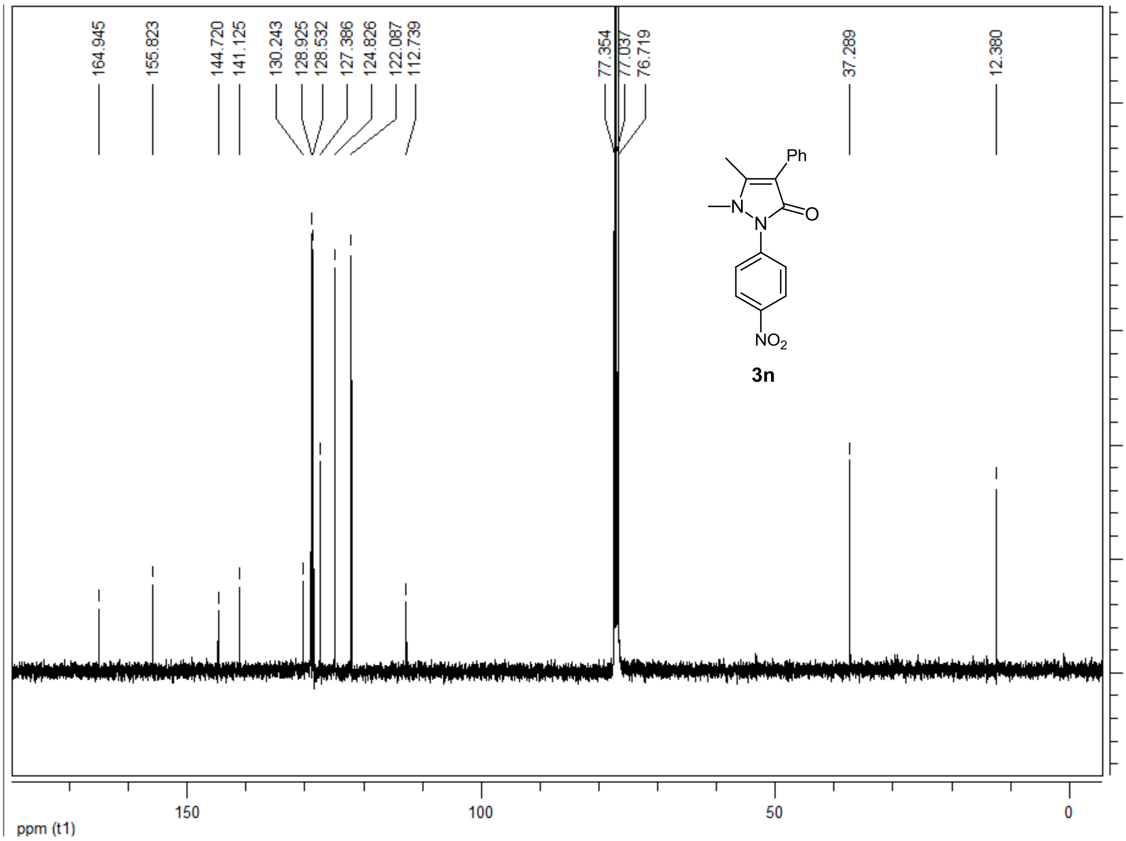

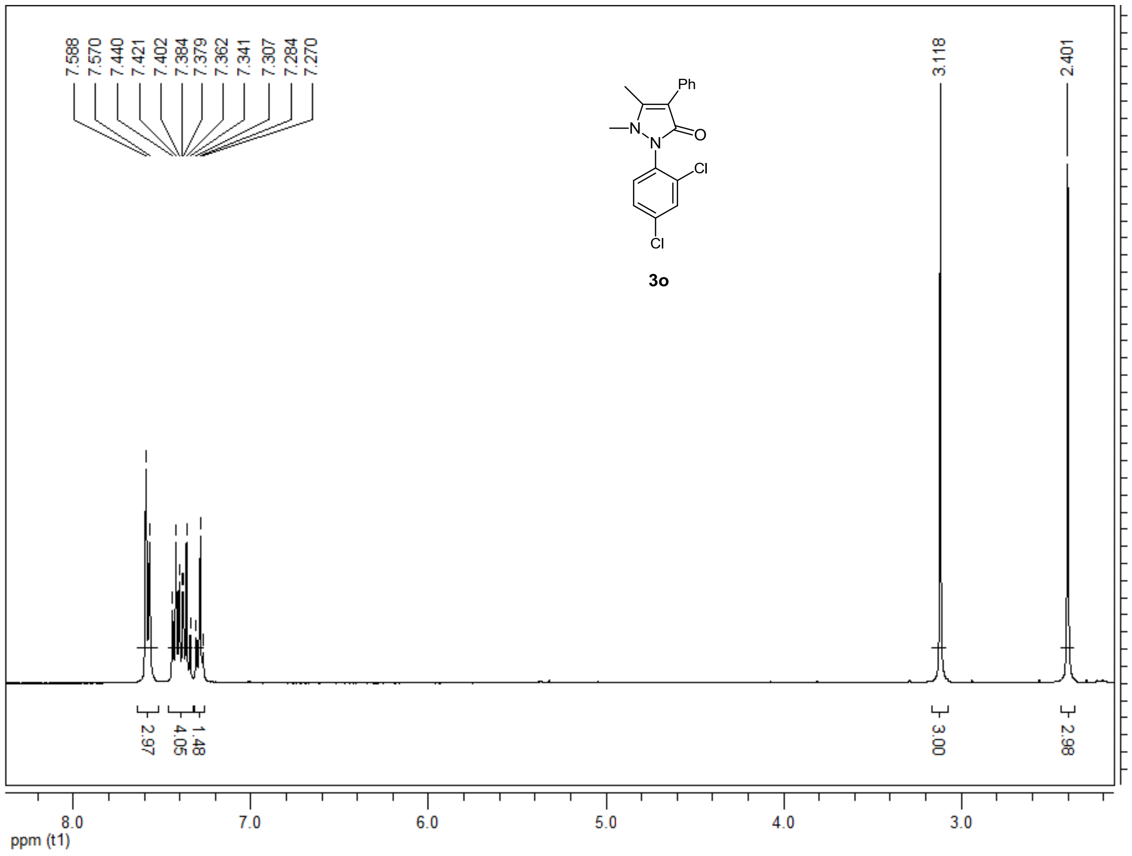
7.588
7.570
7.440
7.421
7.402
7.384
7.379
7.362
7.341
7.307
7.284
7.270
3.118
2.401
Ph
N
N
O
Cl
Cl
3o
2.97
4.05
1.48
3.00
2.98
8.0
7.0
6.0
5.0
4.0
3.0
ppm (t1)

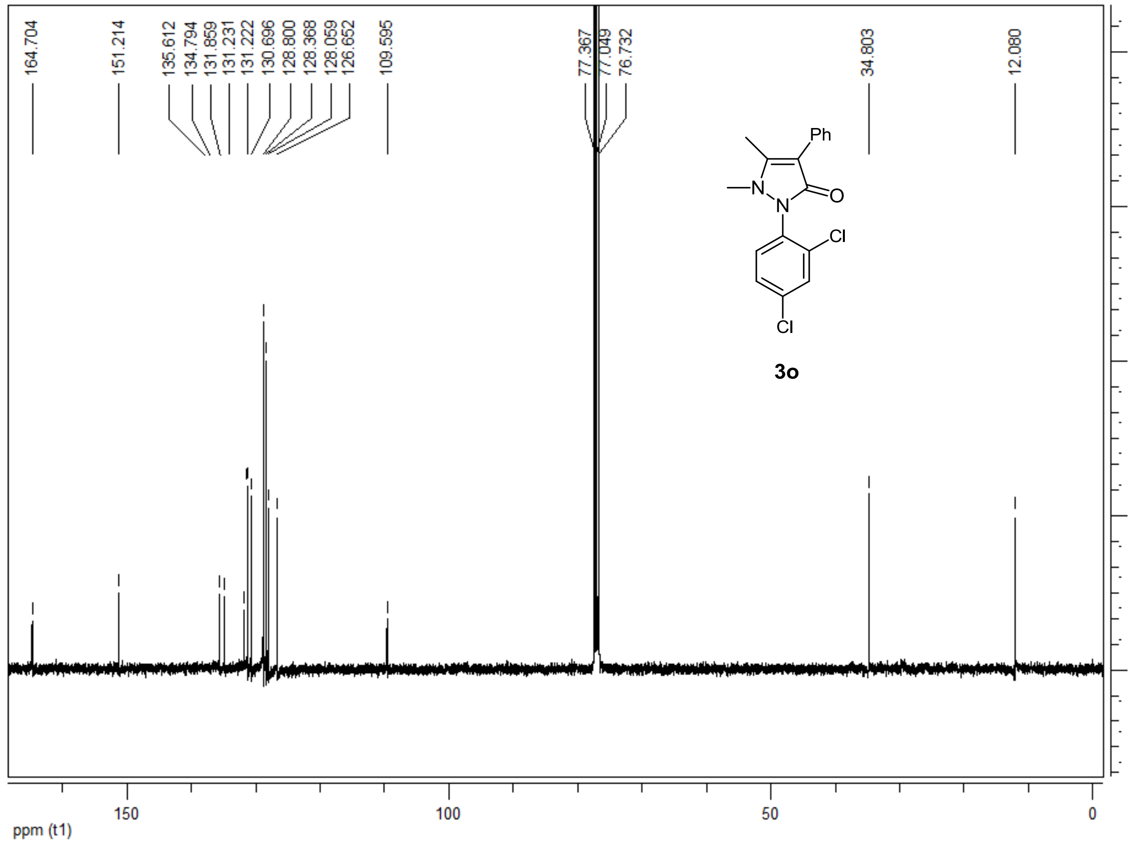
164.704
151.214
135.612
134.794
131.859
131.231
131.222
130.696
128.800
128.368
128.059
126.652
109.595
77.367
77.049
76.732
34.803
12.080
Ph
N
N
O
Cl
Cl
3o
150
100
50
0
ppm (t1)

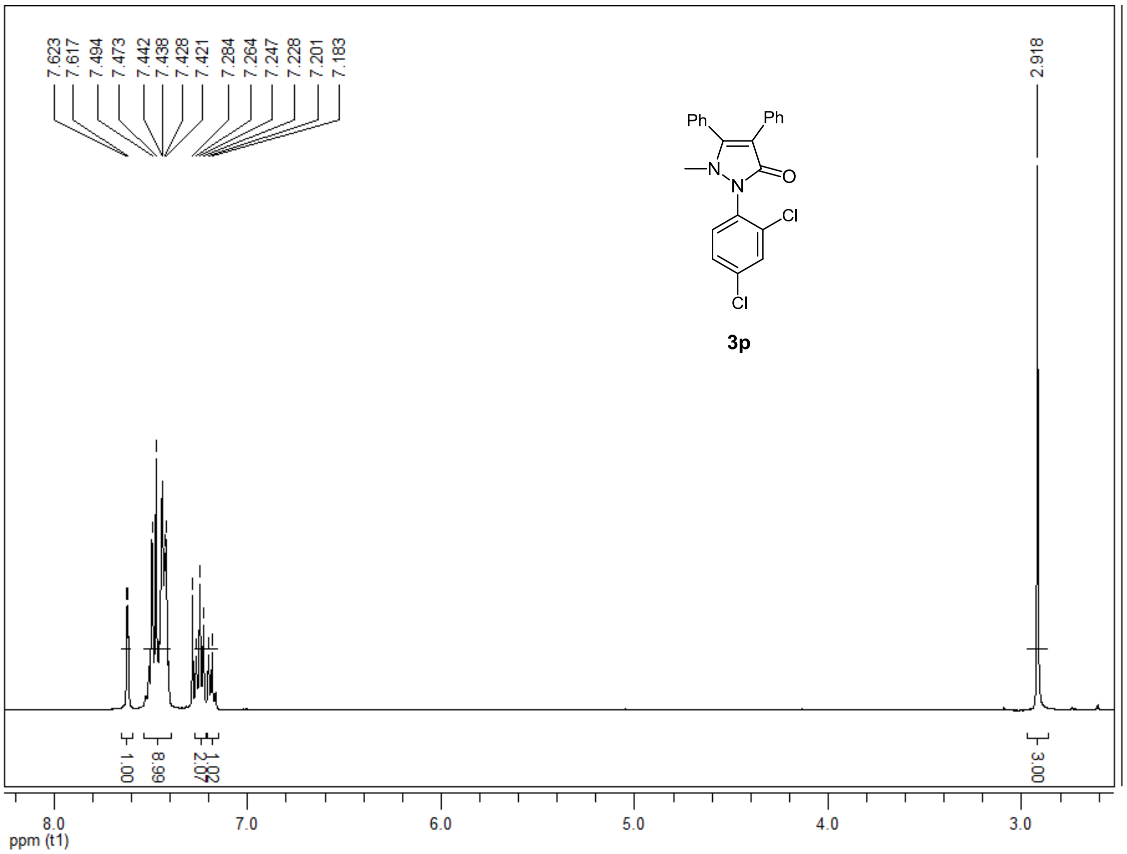
7.623
7.617
7.494
7.473
7.442
7.438
7.428
7.421
7.284
7.264
7.247
7.228
7.201
7.183
2.918
Ph
Ph
N
N
O
Cl
Cl
3p
1.00
8.99
2.07
1.02
3.00
8.0
7.0
6.0
5.0
4.0
3.0
ppm (t1)

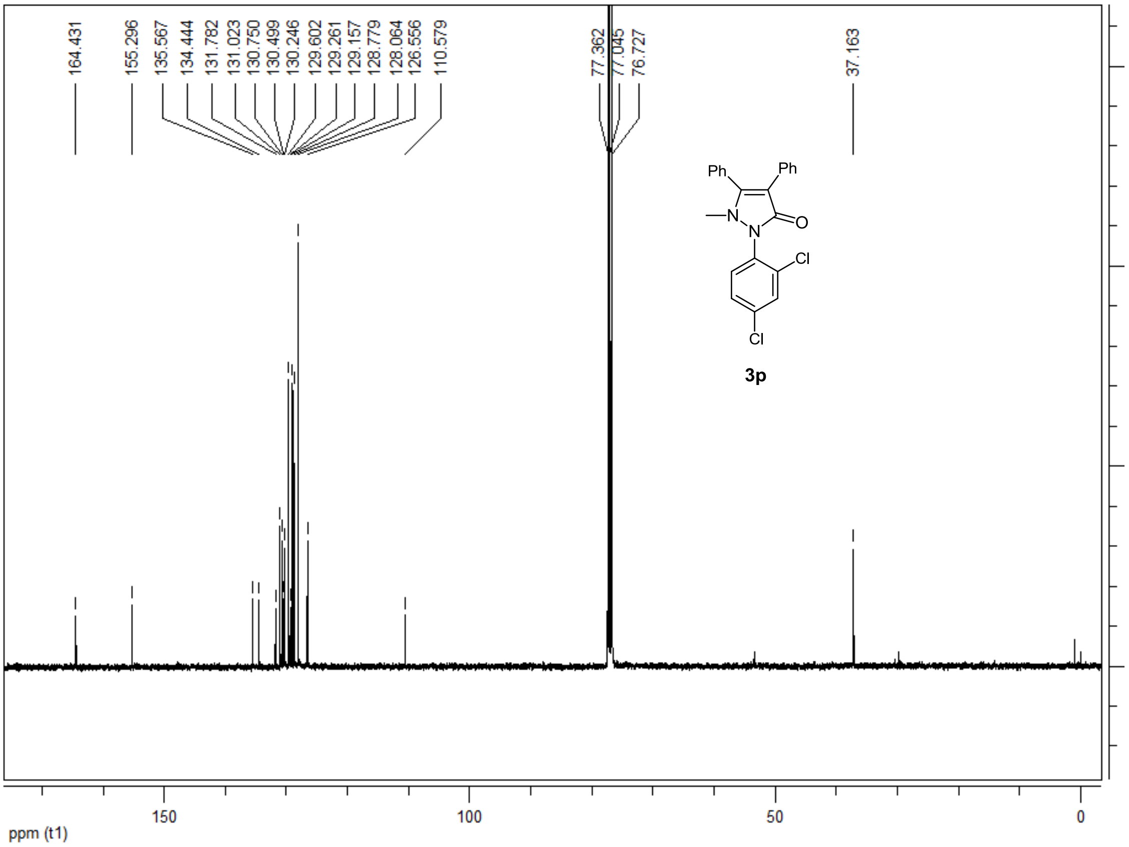
164.431
155.296
135.567
134.444
131.782
131.023
130.750
130.499
130.246
129.602
129.261
129.157
128.779
128.064
126.556
110.579
77.362
77.045
76.727
37.163
Ph
Ph
N
N
O
Cl
Cl
3p
150
100
50
0
ppm (t1)

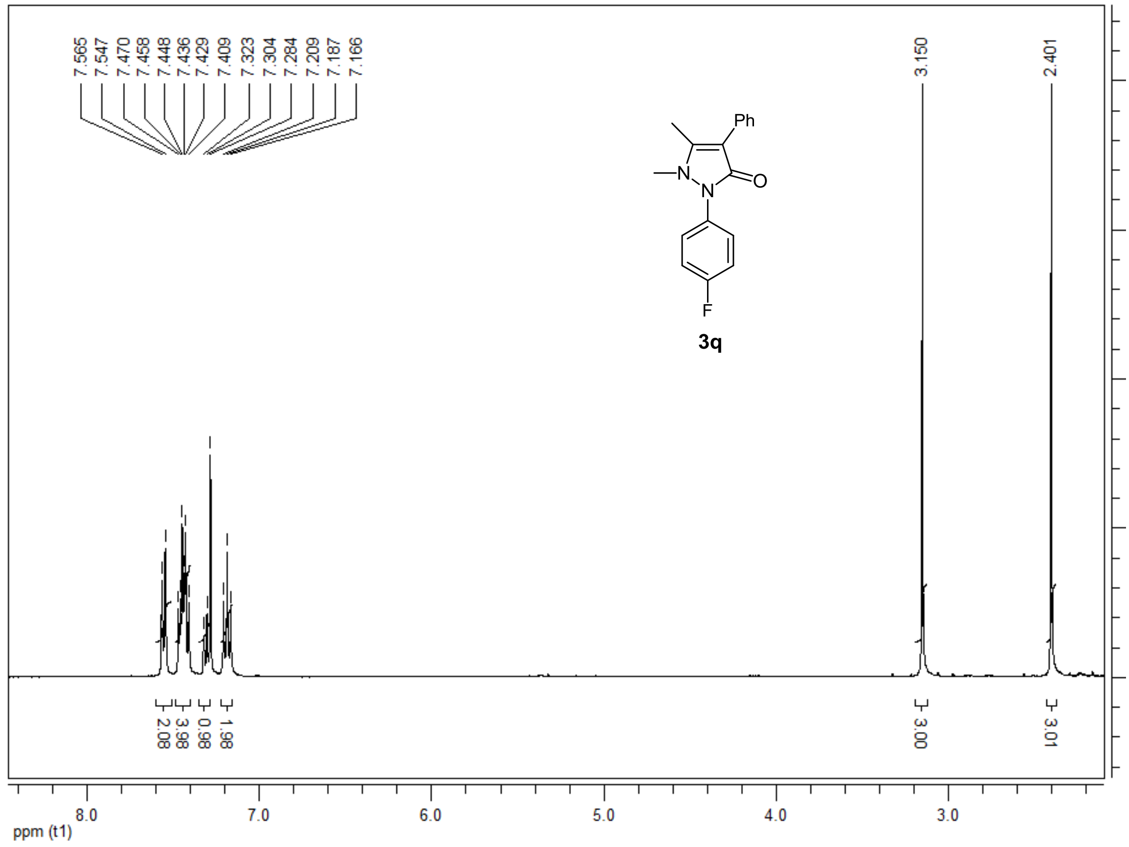

7.565
7.547
7.470
7.458
7.448
7.436
7.429
7.409
7.323
7.304
7.284
7.209
7.187
7.166
3.150
2.401
Ph
N
N
O
F
3q
2.08
3.98
0.98
1.98
3.00
3.01
8.0
7.0
6.0
5.0
4.0
3.0
ppm (t1)

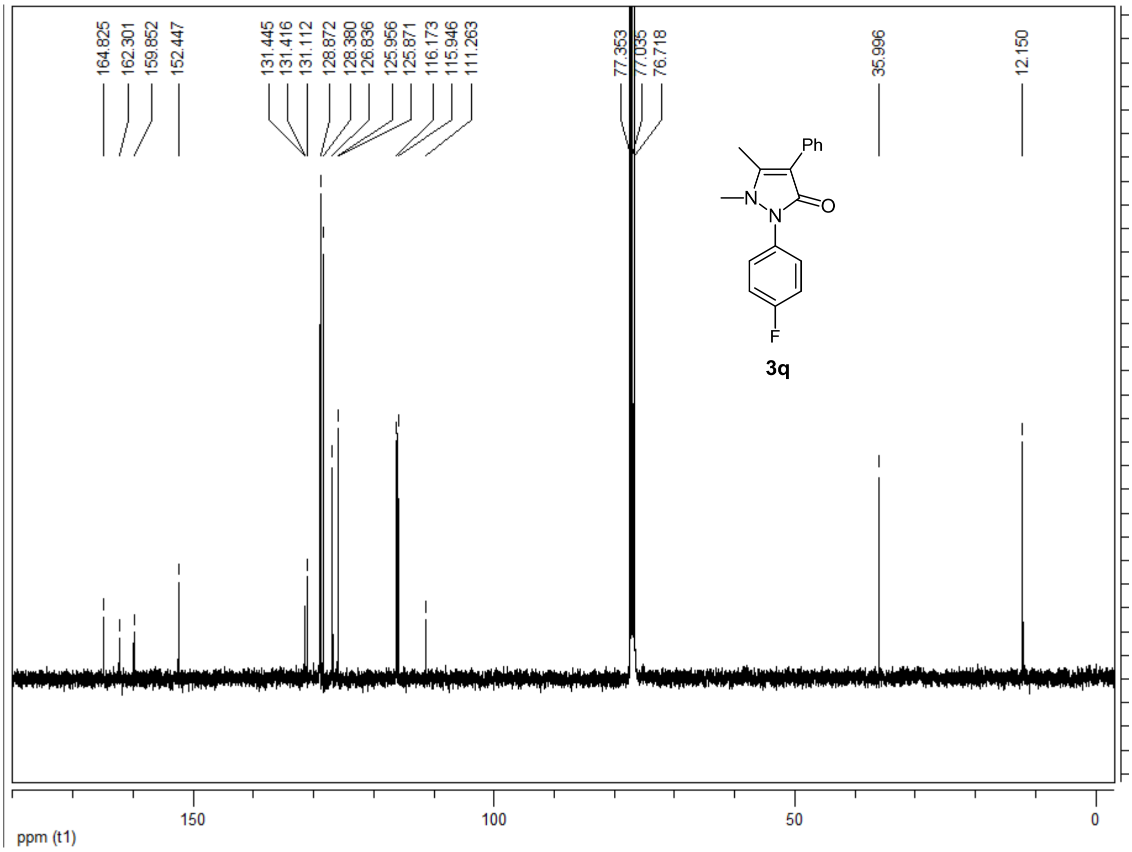

164.825
162.301
159.852
152.447
131.445
131.416
131.112
128.872
128.380
126.836
125.956
125.871
116.173
115.946
111.263
77.353
77.035
76.718
35.996
12.150
Ph
N
N
O
F
3q
150
100
50
0
ppm (t1)

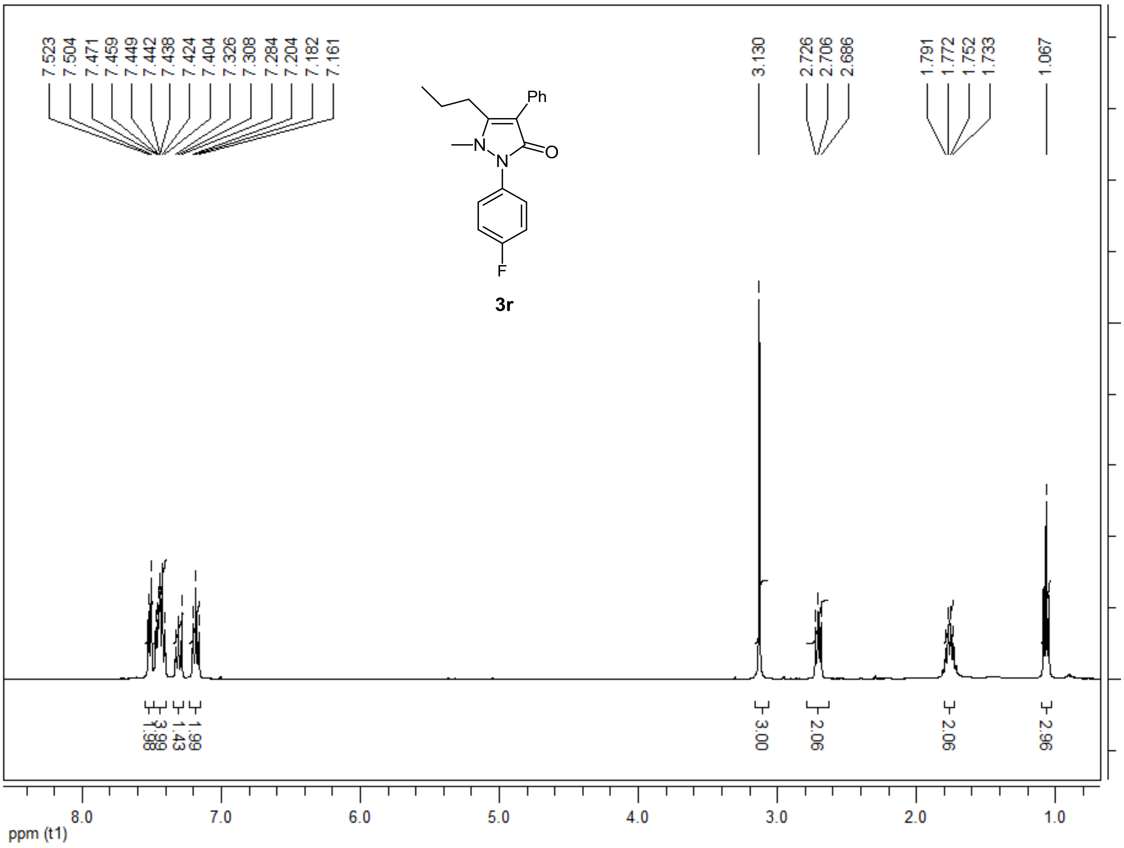
Ph
N
N
O
F
3r
ppm (t1)

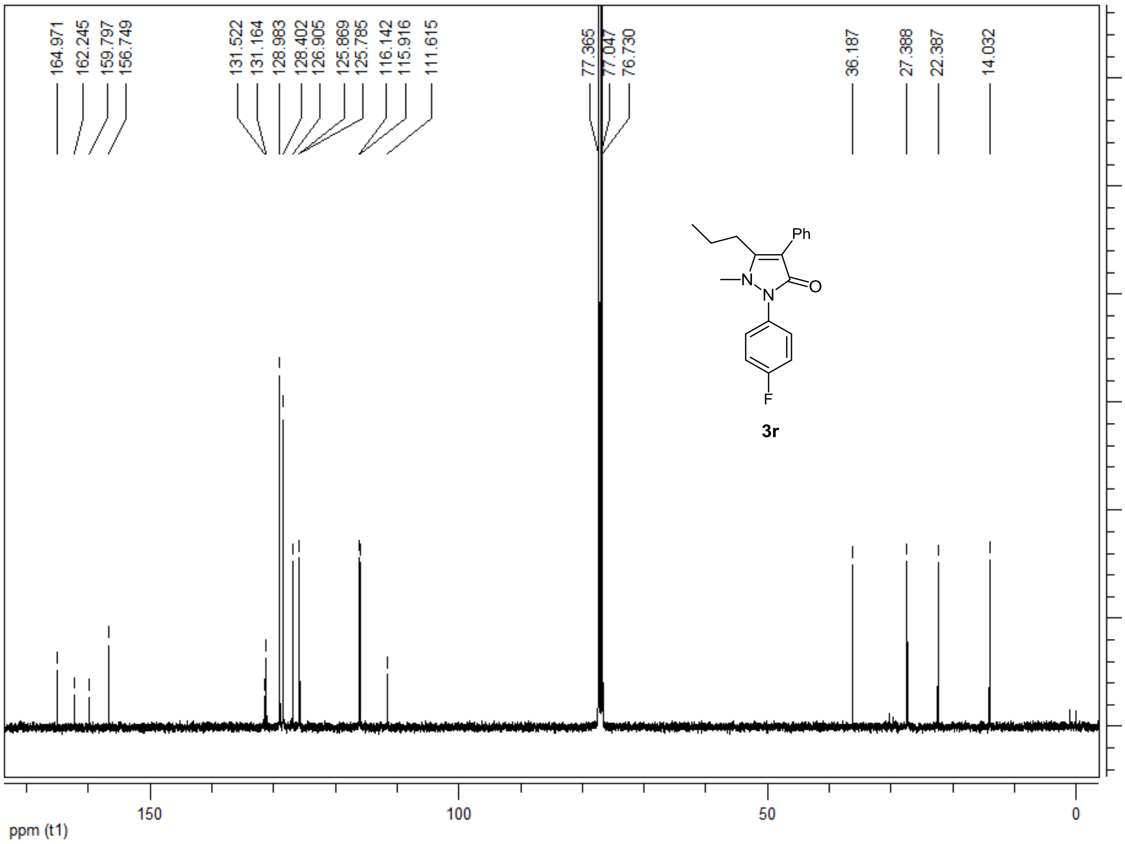
Ph
N
N
O
F
3r
ppm (t1)

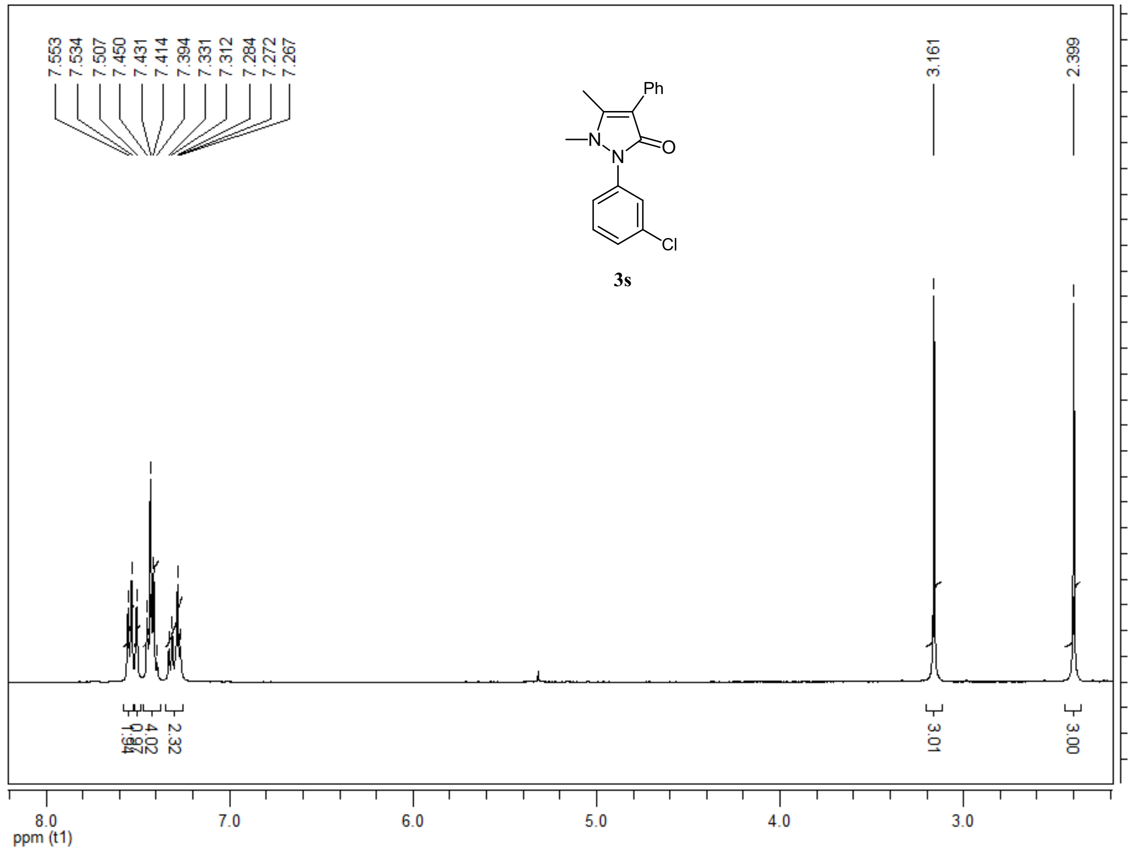
7.553
7.534
7.507
7.450
7.431
7.414
7.394
7.331
7.312
7.284
7.272
7.267
3.161
2.399
Ph
N
N
O
Cl
3s
4.02
2.32
3.01
3.00
8.0
7.0
6.0
5.0
4.0
3.0
ppm (t1)

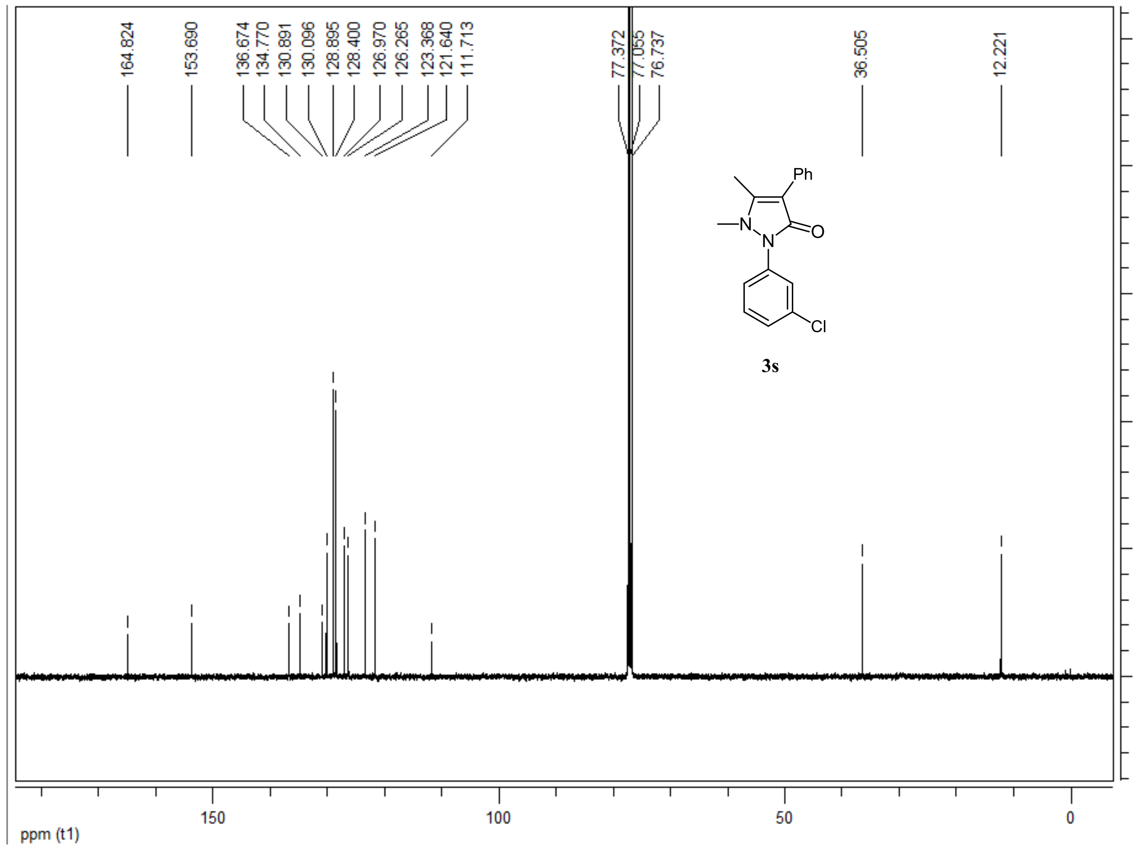
164.824
153.690
136.674
134.770
130.891
130.096
128.895
128.400
126.970
126.265
123.368
121.640
111.713
77.372
77.055
76.737
36.505
12.221
Ph
N
N
O
Cl
3s
150
100
50
0
ppm (t1)

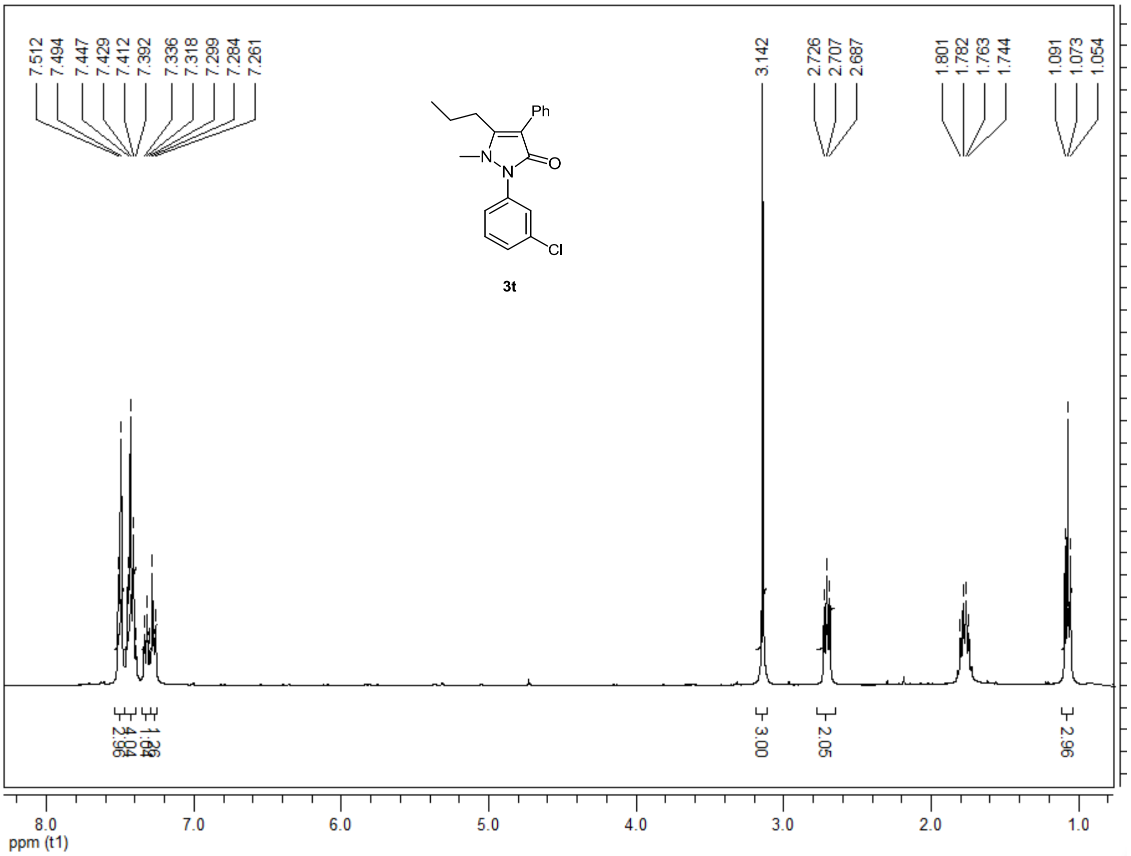
7.512
7.494
7.447
7.429
7.412
7.392
7.336
7.318
7.299
7.284
7.261
3.142
2.726
2.707
2.687
1.801
1.782
1.763
1.744
1.091
1.073
1.054
Ph
N
N
O
Cl
3t
2.96
4.04
1.26
3.00
2.05
2.96
8.0
7.0
6.0
5.0
4.0
3.0
2.0
1.0
ppm (t1)

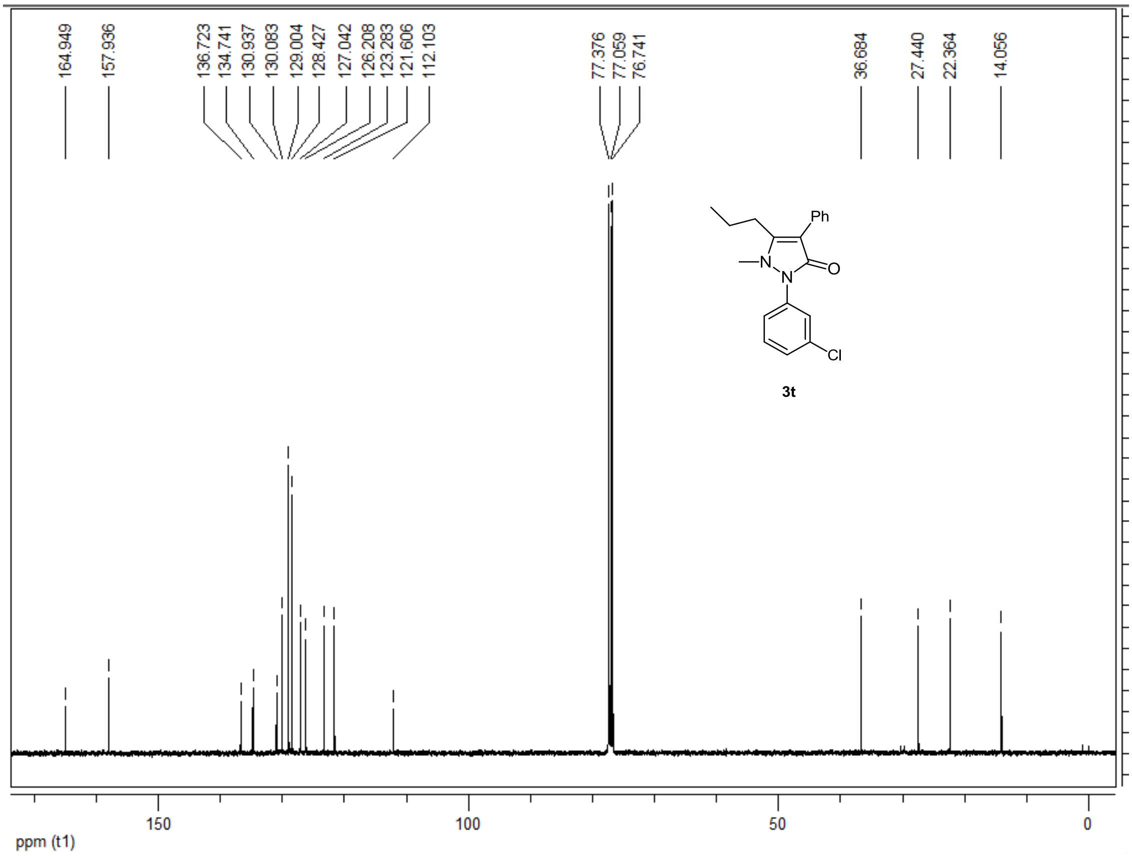
164.949
157.936
136.723
134.741
130.937
130.083
129.004
128.427
127.042
126.208
123.283
121.606
112.103
77.376
77.059
76.741
36.684
27.440
22.364
14.056
Ph
N
N
O
Cl
3t
150
100
50
0
ppm (t1)
